# Characterization and Validation of ceRNA-Mediated Pathway–Pathway Crosstalk Networks Across Eight Major Cardiovascular Diseases

**DOI:** 10.3389/fcell.2022.762129

**Published:** 2022-04-01

**Authors:** Chao Song, Jian Zhang, Yongsheng Liu, Yinling Hu, Chenchen Feng, Pilong Shi, Yuexin Zhang, Lixin Wang, Yawen Xie, Meitian Zhang, Xilong Zhao, Yonggang Cao, Chunquan Li, Hongli Sun

**Affiliations:** ^1^ Department of Pharmacology, Harbin Medical University-Daqing, Daqing, China; ^2^ Department of Medical Informatics, Harbin Medical University-Daqing, Daqing, China; ^3^ Department of Rehabilitation, Beijing Rehabilitation Hospital of Capital Medical University, Beijing, China

**Keywords:** cardiovascular diseases, ceRNA, pathway crosstalk, network analysis, website

## Abstract

Pathway analysis is considered as an important strategy to reveal the underlying mechanisms of diseases. Pathways that are involved in crosstalk can regulate each other and co-regulate downstream biological processes. Furthermore, some genes in the pathways can function with other genes *via* the relationship of the competing endogenous RNA (ceRNA) mechanism, which has also been demonstrated to play key roles in cellular biology. However, the comprehensive analysis of ceRNA-mediated pathway crosstalk is lacking. Here, we constructed the landscape of the ceRNA-mediated pathway–pathway crosstalk of eight major cardiovascular diseases (CVDs) based on sequencing data from ∼2,800 samples. Some common features shared by numerous CVDs were uncovered. A fraction of the pathway–pathway crosstalk was conserved in multiple CVDs and a core pathway–pathway crosstalk network was identified, suggesting the similarity of pathway–pathway crosstalk among CVDs. Experimental evidence also demonstrated that the pathway crosstalk was functioned in CVDs. We split all hub pathways of each pathway–pathway crosstalk network into three categories, namely, common hubs, differential hubs, and specific hubs, which could highlight the common or specific biological mechanisms. Importantly, after a comparison analysis of the hub pathways of networks, ∼480 hub pathway-induced common modules were identified to exert functions in CVDs broadly. Moreover, we performed a random walk algorithm on the hub pathway-induced sub-network and identified 23 potentially novel CVD-related pathways. In summary, our study revealed the potential molecular regulatory mechanisms of ceRNA crosstalk in pathway–pathway crosstalk levels and provided a novel routine to investigate the pathway–pathway crosstalk in cardiology. All CVD pathway–pathway crosstalks are provided in http://www.licpathway.net/cepathway/index.html.

## Introduction

With the increasing scale of high-throughput expression data, pathway analysis has become an important routine for understanding the underlying biological mechanisms of multiple diseases. Recent studies have demonstrated that pathways are involved in a lot of the processes of cardiovascular diseases (CVDs), such as the AMPK signaling pathway (Gelinas et al., 2018), TGF-beta signaling pathway (Xie et al., 2019), and apoptosis (Sun et al., 2021). In addition, emerging evidence is to show that pathways can intersect to regulate each other and co-regulate downstream biological processes (Mohan and Gupta, 2018; Chatterjee and Sil, 2019). Elucidation of the pathway–pathway crosstalk in a large scale contributes to reveal the underlying mechanisms of CVDs. Some studies have proposed the various strategies to identify the crosstalk between pathways based on a statistical distribution model or biological network-based method (Alexeyenko et al., 2012; Glaab et al., 2012; Mccormack et al., 2013; Ogris et al., 2016).

Micro RNAs (miRNAs) are an abundant type of small, non-coding RNAs (∼22 nt long), which can repress the expression of messenger RNAs (mRNAs) *via* the miRNA-induced silencing complex in a post-transcriptional level (Ambros, 2004; Bartel, 2009). Dysregulation of miRNA activity had been demonstrated to play crucial roles in the physiological and pathological processes of CVDs (Raso et al., 2021; Song et al., 2021). Moreover, previous studies had elucidated a novel regulatory relationship between RNAs, named as competing endogenous RNAs (ceRNAs). Numerous studies had found that the RNAs within ceRNAs could act as molecular sponges of the miRNAs by competing for the shared seed regions and then implicate in the complex biological process of diseases (Karreth et al., 2011; Salmena et al., 2011). For instance, the lncRNA CHRF could act as an endogenous sponge of miR-489 to upregulate the expression of Myd88 and promote hypertrophy (Wang et al., 2014). CircRNA HRCR could increase the ARC expression *via* functioning as an endogenous miR-223 sponge to inhibit miR-223 activity in heart failure (Wang, 2016). Our previous studies also predicted the cardiac hypertrophy-associated lncRNAs by biological regulation networks (Song et al., 2016). Pathways encompassed numerous genes; thus, we proposed a novel hypothesis that the ceRNA crosstalk could mediate the crosstalk between pathways.

Cardiovascular diseases have become the leading cause of morbidity and mortality worldwide. Although various types of CVDs showed different symptoms, some common mechanisms had also been demonstrated. For example, both myocardial infarction and cardiac hypertrophy could be regulated by the TGF-beta signaling pathway (Song et al., 2012; Tseliou et al., 2014), and the miR-21 family played important roles in heart failure and pulmonary hypertension (Parikh et al., 2012; Marketou et al., 2013). Moreover, PTEN, a famous tumor suppressor, played crucial roles in the pathology of CVDs and implicated in the PI3K/Akt pathway (Gao et al., 2014). Studies found that ZEB2 mRNA, a bona fide PTEN ceRNA, modulated PTEN protein levels in a microRNA-dependent, protein coding-independent manner (Karreth et al., 2011). Interestingly, Jahan et al. found that the ZEB2 homologous gene Zeb2 was a novel regulator of cardiac fibroblast to myofibroblast transition by repressing the expression of Meox2, which was a new target gene of the TGF-β/Smad pathway (Valcourt et al., 2007; Cunnington et al., 2014). Thus, a potential mechanism appeared that the ceRNA pair PTEN–ZEB2 might mediate a pathway–pathway crosstalk between PI3K/Akt and TGF-β/Smad pathways. In our previous study, we have identified the CVD-related ceRNA crosstalk and investigated some characteristics of the ceRNA-mediated pathway crosstalk (including the types of within pathway and between pathway). Results showed that the ceRNA-mediated pathway crosstalk has strong regulatory potentials (Song et al., 2017). However, a comprehensive analysis that focuses on the ceRNA-mediated pathway–pathway crosstalk in CVDs is absent. These indicated the necessity to identify similar or specific mechanisms among CVDs. The appearance and rapid development of high-throughput techniques that could detect the expression levels of RNA transcripts, such as microarray and RNA-seq, enabled us to investigate the ceRNA crosstalk in transcriptional level. Studies had also found that crosslinking and Argonaute (Ago) immunoprecipitation coupled with high-throughput sequencing (CLIP-seq) could identify the genome-wide interaction of miRNAs and their target-RNAs (Helwak et al., 2013). In addition, the CLIP-seq provided by starBase supported miRNA–mRNA interactions freely (Li et al., 2013). MSigDB, a collection of annotated gene sets for use with GSEA software, provided ∼1,000 known pathways in various pathway databases. By integrating all the CVD-associated ceRNA crosstalk identified from our previous study and pathways collected in MSigDB, these could offer new light to investigate the global pathway–pathway crosstalk in CVDs.

Here, we systematically investigated the global ceRNA-mediated pathway–pathway crosstalk in eight major CVDs by integrating the analysis of ∼2,800 CVD samples and ∼1,200 pathways. First of all, the topological analysis was performed for all pathway–pathway crosstalk networks and some conserved features were identified across CVDs. A common pathway–pathway core network was also highlighted from our research. Next, we split the hubs in each disease network into three types based on the neighbor similarity. Importantly, some common modules were identified by integrating the analysis of common, differential hubs and known CVD-related pathways. Moreover, the algorithm of random walk with restart was performed in the pathway–pathway crosstalk network to optimize novel CVD-related pathways. Summarily, all these results revealed the crucial roles of the ceRNA-mediated pathway–pathway crosstalk in multiple CVDs and proposed a novel molecular mechanism to exert functions in cardiology.

## Materials and Methods

### Cardiovascular Disease-Related Microarray Datasets

All the cardiovascular disease-related expression profiles were downloaded from GEO (https://www.ncbi.nlm.nih.gov/geo/), which was a public functional genomics data repository supporting MIAME-compliant data submissions. Specifically, we investigated eight major CVDs in this study, including coronary artery disease (CAD), hypertrophic cardiomyopathy (HCM), dilated cardiomyopathy (DCM), ischemic cardiomyopathy (ICM), heart failure (HF), myocardial infarction (MI), pulmonary hypertension (PAH), and congenital heart disease (CHD) from 21 gene expression datasets. All these profiles were used to identify ceRNA pairs based on our previous pipeline (Supplementary Material and Supplementary Table S1) (Song et al., 2017).

### mRNA-Related Competing Endogenous RNA Pairs

In this study, we obtained all the mRNA-related ceRNA pairs of each CVD-related expression profile from our previous study (Supplementary Material and Supplementary Table S1) (Song et al., 2017), which were identified by integrating CLIP-seq-supported miRNA–mRNA interactions and candidate co-expressed mRNA–mRNA pairs.

### Acquisition of Pathway Data

All the pathways encompassing curated gene sets were downloaded from the C2 group of MsigDB V5.2 (http://software.broadinstitute.org/gsea/msigdb/index.jsp), which was a collection of annotated gene sets for use with GSEA software. We only reserved the pathways from four popular databases, including KEGG, BIOCARTA, REACTOME, and PID. In total, 1,273 pathways were collected for further research.

### Collection of Cardiovascular Disease-Associated miRNAs

Several bioinformatics databases have provided a comprehensive resource of miRNA-disease relationships. We downloaded CVD-associated miRNAs from HMDD (http://www.cuilab.cn/hmdd), miR2Disease (http://www.mir2disease.org/), and miREnvironment (http://cmbi.bjmu.edu.cn/miren), and mapped these miRNAs to each of the eight CVDs performed in our study manually. As a result, 702 miRNA-CVD pairs were collected totally after combining various databases.

### Construction of Competing Endogenous RNA-Mediated Pathway–Pathway Crosstalk Networks

**FIGURE 1:**
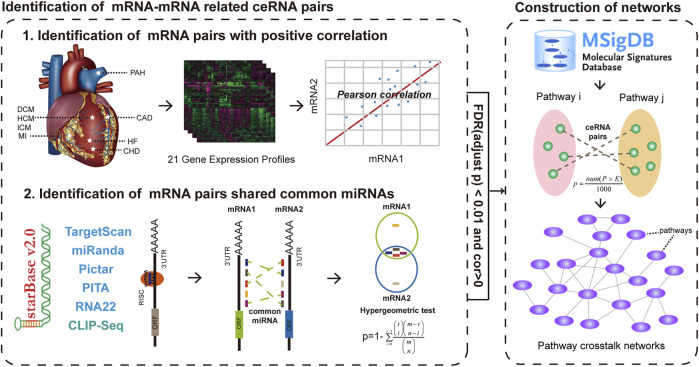
Global workflow for construction of ceRNA-mediated pathway crosstalk networks of eight major CVDs. After identifying ceRNA crosstalk pairs by integrating gene expression and hypergeometric test in each gene expression profiles, we mapped these ceRNAs into the pathways that were downloaded from MSigDB. All candidate pathway crosstalks with a *p* value < 0.01 after 1,000 times permutation were identified as pathway crosstalks. Then, 21 CVD-related pathway crosstalk networks were constructed.

Having got the ceRNA crosstalk pairs of each CVD-related expression profile that was identified from our previous study, we then followed two steps to identify the significant pathway–pathway crosstalk (Figure 1). A principle of our hypothesis was that the activity of the pathway–pathway crosstalk was increased with the number of ceRNA crosstalk pairs between two pathways. For each pathway–pathway crosstalk pair, firstly, we counted the number of ceRNA pairs between pathways and removed the pathway–pathway pairs with less than five ceRNA pairs. Secondly, a permutation test was applied to yield the statistical power of each pathway–pathway crosstalk pair. In detail, the pathway sizes were held constant and genes were randomly selected to perform the first step for 1,000 times, and generating a set of 1,000 permutation numbers (P). An empirical *p* value of one pathway–pathway crosstalk was calculated as follows:

$$\mathrm{p value=}\frac{\mathrm{num}\left(p>E\right)}{1000}.$$

All the candidate pathway pairs with *p* value < 0.01 were considered as pathway–pathway crosstalk pairs.

After identifying all pathway–pathway crosstalk pairs, the ceRNA-mediated pathway–pathway crosstalk networks of each CVD-related profile were constructed and all the networks were visualized by Cytoscape (http://www.cytoscape.org/). The nodes of networks represented pathways and edges represented the significant crosstalk between the pathways.

### Topological Feature Analysis of Pathway–Pathway Crosstalk Networks

In each network, the degree of one node represented the edges connecting to it. Previous studies had found that the hub node with high degrees tended to play crucial roles in the biological networks. Thus, we ranked all the degrees of the nodes and selected the top 20% nodes with higher degrees as the hub nodes. To perform a comprehensive analysis of the features of hub pathways across all CVDs, we split the hubs into three classes by applying the strategy proposed by Xu *et al*. (2015). We defined three classes of hubs as CVD-specific pathway hub nodes, differential pathway hub nodes, and common pathway hub nodes. In brief, the CVD-specific hub nodes are hub pathways in only one pathway–pathway crosstalk network. Hub pathways that occurred in more than one pathway–pathway crosstalk network but their neighbors changed between different networks are defined as differential hubs. Common hub nodes are hub pathways occurring in more than one pathway–pathway crosstalk network with similar neighbors. All the topological features were performed by the packages of “igraph” in R language.

For the first category, the pathways only ranked in the top 20% of the highest degrees in one pathway–pathway crosstalk network. To identify the other two types of hubs, we calculated the similarity of their interacting neighbors between pairs of pathway–pathway crosstalk networks by the method of “Simpson index” (Xu et al., 2015). Briefly, the Simpson index was calculated as follows:

$$\mathrm{Simpson index}\left(A\text{,}B\right)=\frac{\left|\text{A}\cap \text{B}\right|}{\text{min}\left(\left|\text{A}\right|,\left|\text{B}\right|\right)},$$

where A and B are the pathway neighbor sets of the specific hub pathway in two pathway crosstalk networks. If the hub node was with Simpson index >0.8 in at least one pair of pathway–pathway crosstalk networks, we split the hub node into the third category. Otherwise, we sorted the hub nodes into the second category.

### Known Cardiovascular Disease-Related Pathways

Known CVD-related pathways were downloaded from the KEGG pathway database with focus on the category of “cardiovascular diseases” and their one-step associated pathways. Next, we calculated the similarity between all pathways in MSigDB and known CVD-related pathways in KEGG by the method of Simpson index. If the pathways were with Simpon index >0.6, we considered them as the known CVD-related pathways for further analysis.

### Identification of Cardiovascular Disease-Associated Pathway–Pathway Crosstalk Modules

To identify the common CVD-associated pathway–pathway crosstalk modules, firstly, we mapped all known CVD-related pathways to each disease pathway–pathway crosstalk network and all the sub-networks were merged. Next, we extracted the common hub pathway and differential hub pathway-associated component of the merged sub-network. Finally, we performed the CFinder (http://www.cfinder.org/), a fast program for locating and visualizing overlapping, to identify the cliques as conserved modules. Moreover, we only reserved the modules that contained more than five pathways. Similarly, the specific pathway–pathway crosstalk modules were identified by mapping specific hub pathways into the sub-networks of each disease.

### Identification of a Novel Cardiovascular Disease-Related Pathway Based on Pathway–Pathway Crosstalk Network

To identify more CVD-related pathways, we performed a random walk on a hub pathway-induced sub-network, which was constructed by merging all hub pathway–hub pathway pairs of each disease pathway network into a component (Song et al., 2016). The random walk with restart in our study was defined as

$${\text{p}}^{\text{t}+1}=\left(1-\text{r}\right){\text{Wp}}^{\text{t}}+{\text{rp}}^{0},$$

where W represents the column-normalized adjacency matrix of the network, p^t^ is a vector whose size is equivalent to the number of nodes in the network, and the *i*-th element holds the probability of being at node i at time step t.

In this section, the initial probability vector p^0^ was constructed such that one is assigned to the nodes representing known CVD-related pathways, and other nodes with zero. We believe that the role of CVD-related pathways is equivalent in the network. Vector p will be in the steady-state at time step t where t approaches infinity as a limit. The iteration will be finished when the change between p^t^ and p^t+1^ falls below 10^−10^. Moreover, the statistical significance for rejection of the null hypothesis was determined by comparing the scores of the pathways in the network following n iterations of that known CVD-related pathways shuffling. In iterations, the times that the score of every pathway was higher than the real one was recorded as m. The statistical significance *p* value for each pathway was calculated by m/n. In this work, n was set at 5,000 times.

### Animal Models of Cardiac Hypertrophy and Myocardial Infarction

All experimental procedures conformed to the Guide for the Care and Use of Laboratory Animals published by the US NIH (publication, 8th Edition, 2011). Our study conformed to the regulations of the Ethics Committee of Harbin Medical University. Transverse aortic constriction (TAC) models were used to construct cardiac hypertrophy models based on our previous protocol (Song et al., 2021). For myocardial infarction models, mice were anesthetized with pentobarbital sodium (30 mg/kg, i.p.). Acute myocardial infarction was induced by ligation of the left anterior descending coronary artery in mice. In the sham group mice, the suture was placed beneath the left coronary artery without ligating.

### Cell Culture and Transfection

HL-1 cell lines were cultured in the recommended DMEM medium, containing 10% fetal bovine serum (HiClone), 100 units/ml penicillin, and 100 mg/ml streptomycin (Beyotime). The cells were grown at 37°C and a 5% CO_2_ atmosphere. Angiotensin-II (Ang-II, #RAB0010, Sigma-Aldrich, St. Louis, MO, United States) 100 nmol/L was treated to induce cardiomyocyte hypertrophy *in vitro* for 24 h. Small interfere RNA (siRNA) and X-treme GENE siRNA (Roche, Penzberg, Germany) were mixed, which were transferred and co-cultured with cells to knockout target genes. The PI3K siRNA sequence was Forward: GGG​ACC​CAC​UAU​CUG​AAA​UTT, Reverse: AUU​UCA​GAU​AGU​GGG​UCC​CTT. The TNF-α siRNA sequence was Forward: GAC​AAC​CAA​CUA​GUG​GUG​CTT, Reverse: GCA​CCA​CUA​GUU​GGU​UGU​CTT. The negative control sequence was Forward: UUC​UCC​GAA​CGU​GUC​ACG​UTT, Reverse: ACG​UGA​CAC​GUU​CGG​AGA​ATT.

### Western Blotting

Total protein was extracted from fresh tissues and cells based on the previous protocol (Song et al., 2021). Briefly, 50 μg protein samples were separated in 10% SDS-PAGE gel and transferred onto a nitrocellulose membrane. After 5% non-fat milk blocking, the blots were incubated with primary antibodies including BNP (1:200 dilution, sc-271185, Santa Cruz), β-MHC (1:500 dilution, HPA001239, Sigma), PI3K (1:2,000 dilution, A12484, Abclonal), LC3-II (1:2,000 dilution, A11534, Abclonal), Akt (1:2,000 dilution, A16343, Abclonal), and β-actin (1:5,000 dilution, KC5A08, Kangcheng) at 4°C overnight. Imaging System (LI-COR Biosciences) and ImageJ were used to perform a statistical analysis.

### Quantitative RT-PCR (qRT-PCR)

Total RNA from heart tissues and cells was extracted with Trizol (Invitrogen) according to the manufacturer’s protocol. cDNA was synthesized with reverse transcriptase (Takara, Japan). The relative gene expression was normalized to the Gapdh level in each sample. The primer sequences were synthesized by Sangon Biotech (Shanghai) Co., Ltd.

## Results

### Competing Endogenous RNA-Mediated Pathway–Pathway Crosstalk in Cardiovascular Diseases

To systematically investigate the potential roles of the pathway–pathway crosstalk in CVDs, we performed two steps to construct the ceRNA crosstalk-mediated pathway–pathway crosstalk network in 21 sets of CVD-related expression profiles, including coronary artery disease (CAD), hypertrophic cardiomyopathy (HCM), dilated cardiomyopathy (DCM), ischemic cardiomyopathy (ICM), heart failure (HF), myocardial infarction (MI), pulmonary hypertension (PAH), and congenital heart disease (CHD). All the ceRNA pairs of each expression profile identified from our previous study were used for counting the number of ceRNA crosstalk between all candidate pathway–pathway pairs. For a candidate pathway–pathway pair, a 1,000 times permutation test was performed to yield the statistical significance. We only reserved the pathway–pathway pairs that met the threshold of *p* < 0.01. After merging all significant pathway–pathway pairs of each profile into a network, 21 pathway–pathway crosstalk networks were constructed. All these networks can be viewed in Supplementary Figure S1. As a result, the ceRNA-mediated pathway crosstalk in different diseases or datasets were different. For instance, in the dataset GSE3586 of dilated cardiomyopathy, only 623 pathway pairs were implicated in the ceRNA-mediated pathway–pathway crosstalk, which account for 21% of the total pathway–pathway crosstalk. However, in the dataset GSE33463 of pulmonary hypertension, the data was increased to 58,384 pairs and 94.9% (Supplementary Table S1). These results indicated that the ceRNA-mediated pathway–pathway crosstalk have disease-specific characteristics.

### Common Topological Properties of the Pathway–Pathway Crosstalk Networks

**FIGURE 2:**
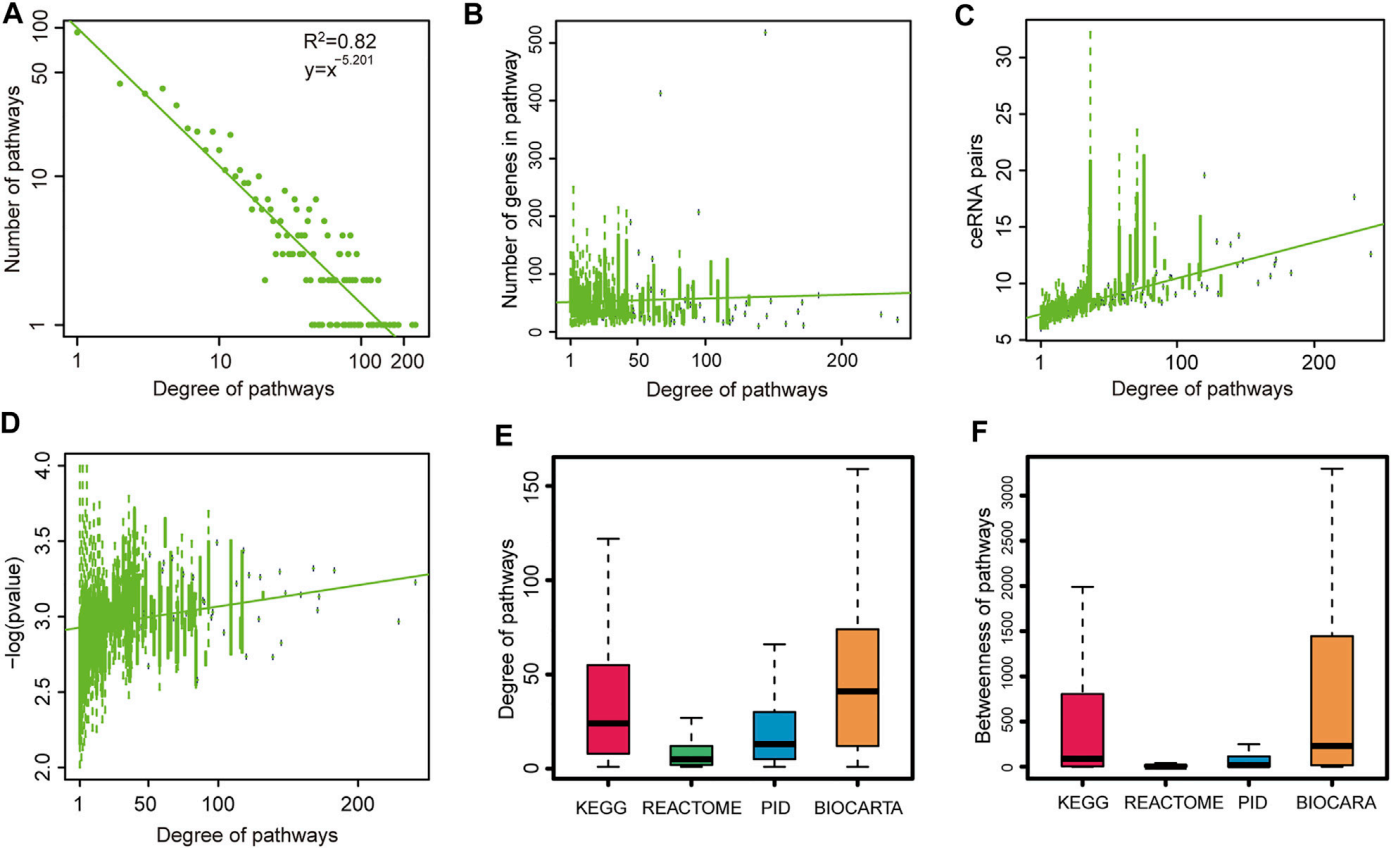
Global topological features of pathway crosstalk networks. Here, we show topological features of one network (GSE1145) and others are shown in Supplementary Figures. **(A)** Degree distributions of network. **(B)** Pathways with high degree encompassed more genes than others. **(C)** Pathways with high degree occupied more ceRNA pairs than others. **(D)** Pathways with high degree had more crosstalk activities than others. We replaced the *p* value = 0 with *p* value = 0.0001. **(E)** Degree distributions of four pathway databases. **(F)** Betweenness distributions of four pathway databases.

To investigate the global properties of pathway–pathway crosstalk in CVDs, the topological features of crosstalk networks across CVDs were analyzed. First of all, degree distributions of all pathway–pathway crosstalk networks were examined. We found that all these networks followed a power law distribution (Figure 2A and Supplementary Figure S2), that was to say all these networks were similar to most biological networks and a small subset of pathway nodes, which defined as hub nodes linked to a majority of pathway nodes in networks. To validate whether the size of the pathways influenced the degrees of pathways in networks or not, we used boxplots to show the trend between degrees of pathways and the capacities of pathways. The results showed that the highly connected pathways tended to have large sizes in most of the networks (Figure 2B and Supplementary Figure S3). In addition, we also found that the pathways with higher degrees occupied more ceRNA pairs (Figure 2C and Supplementary Figure S4) and showed a more significant crosstalk activity (Figure 2D and Supplementary Figure S5). These results indicated that the pathways with more genes were more central in biological networks. That was to say, a large-size pathway might play a more crucial role in the ceRNA-related regulatory mechanisms of CVDs than the small-size pathways. We considered that these results reflected the real biological processes, and the pathways that encompassed more genes had a better chance to inhibit or activate other pathways. For example, all known crucial pathways, such as “KEGG_MAPK,” “KEGG_APOPTOSIS,” and “KEGG_TGF_BETA” pathways all contained more than 80 genes. However, to overcome the bias from the pathway scale, we also used an alternative way to measure the importance of pathways from an average perspective. Here, for the dataset GSE1145, we calculated the average Pearson correlation coefficients of ceRNA pairs in each pathway. Compared with the pathway scale, results showed that the pathways with higher degrees also had the higher ceRNA interactive activity (Supplementary Figure S6).

Moreover, to investigate the differences of connectivity among different origin pathways, we performed the topological feature analysis to the four groups of pathways belonging to four pathway databases. By integrating the analysis results of degree (Figure 2E and Supplementary Figure S7), betweenness (Figure 2F and Supplementary Figure S8), and closeness (Supplementary Figure S8) of the four groups of pathways, we could see that the pathways from the BIOCARTA and KEGG databases covered more essential nodes in the networks than pathways in the other two pathway databases. We presumed that the pathways in the latter two databases were in a low abundance on the ceRNA coverage scale. These results revealed that the pathways from the BIOCARTA and KEGG databases encompassed more crucial pathways and might be involved in more pathology processes of CVDs.

### A Common Core Network Was Highlighted by Network Analysis

**FIGURE 3:**
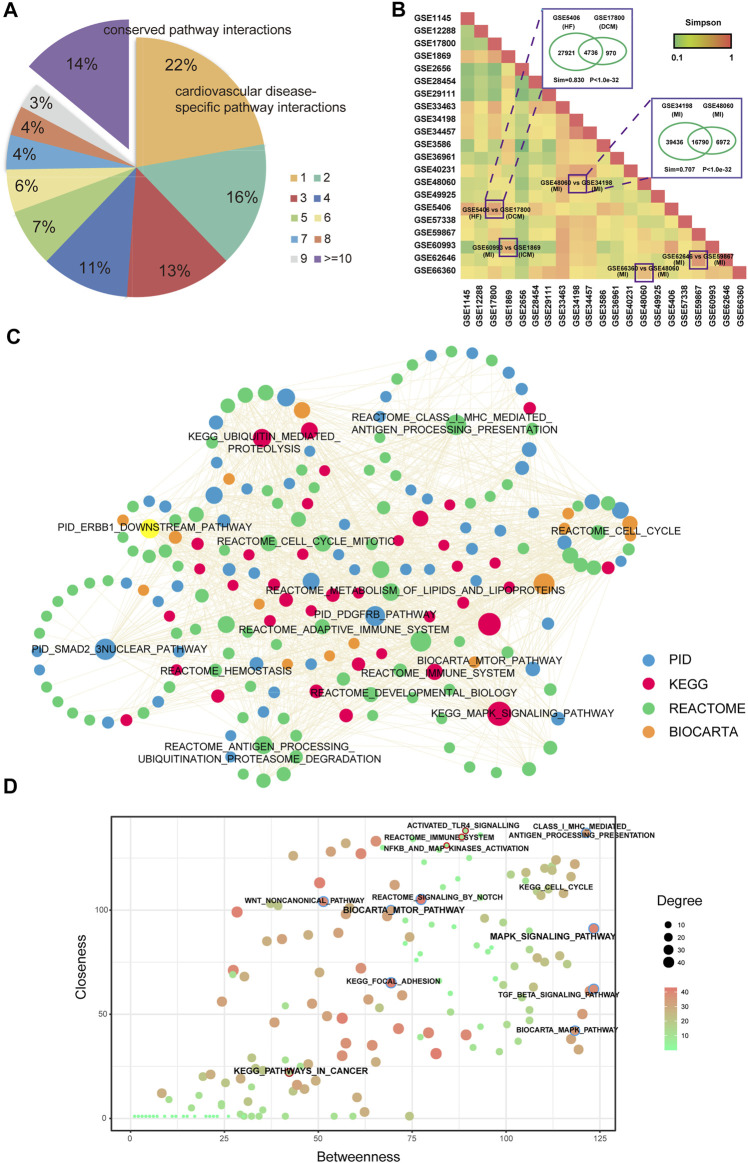
Comparison analysis of pathway crosstalk across multi-CVDs. **(A)** Pie chart of distributions of pathway crosstalk in multi-CVDs. **(B)** Heatmap of the Simpson index represents the similarity between each pair of pathway crosstalk networks. **(C)** A common core pathway crosstalk network. **(D)** A pathway–pathway crosstalk of common core pathway crosstalk network.

Although some common features were identified in multi-CVDs by integrating the analysis of the networks, in the global view to compare all these CVD related pathway–pathway crosstalk networks, we found that ∼22% pathway–pathway crosstalk only occurred in one network. In addition, ∼14% pathway–pathway crosstalk were conserved in more than ten pathway–pathway crosstalk networks (Figure 3A), which indicated that a subset of pathway–pathway crosstalk might form a stable framework to work in all CVDs. To systemically analyze the similarities of pathway–pathway crosstalk between any two networks, we performed an algorithm named Simpson index to measure the similarities between networks (Figure 3B). We found that the pathway–pathway crosstalk networks belonged to the same CVD and the same microarray platforms showed a higher similarity. From the results of the Simpson index, the network of HF (GSE5406) shared a large amount of pathway pairs with the network of DCM (GSE17800) (Sim = 0.830, *p* value < 1e-30). Interestingly, the pathology outcome of dilated cardiomyopathy was heart failure, so we inferred that a lot of pathway–pathway crosstalk should be conserved between DCM and HF, and the results supported our presumption. Furthermore, there existed some similar pathology mechanisms between ischemic cardiomyopathy and myocardial infarction, and in our result, a high Simpson index score (Sim = 0.707, *p* value < 1e-30) was measured between the network of ICM (GSE60993) and the network of MI (GSE 1869). Moreover, the networks from the same disease shared more pathway–pathway pairs. For instance, the network of MI (GSE48060) and the network of MI (GSE34198) were both myocardial infarction-related networks, which got a high Simpson index in our results. Taken together, the pathway–pathway crosstalk networks revealed high similarities of biological processes among CVDs in the pathway level.

To assess whether there was a common core sub-network to maintain the regulatory mechanisms of pathway–pathway crosstalk across multiple CVDs, we extracted pathway–pathway crosstalk that occurred in more than 15 networks and these pathway pairs formed a connected component (Figure 3C), which might play key roles in the pathology of CVDs in pathway levels. In addition, we extracted the topological features for the common core network, and results showed that MAPK pathways exhibited high degree and betweenness, whereas inflammation-related pathways exhibited high closeness. Interestingly, pathways in cancer exhibited non-central topological features (Figure 3D).

### Validation of the Pathway Crosstalk in Cardiovascular Diseases

**FIGURE 4:**
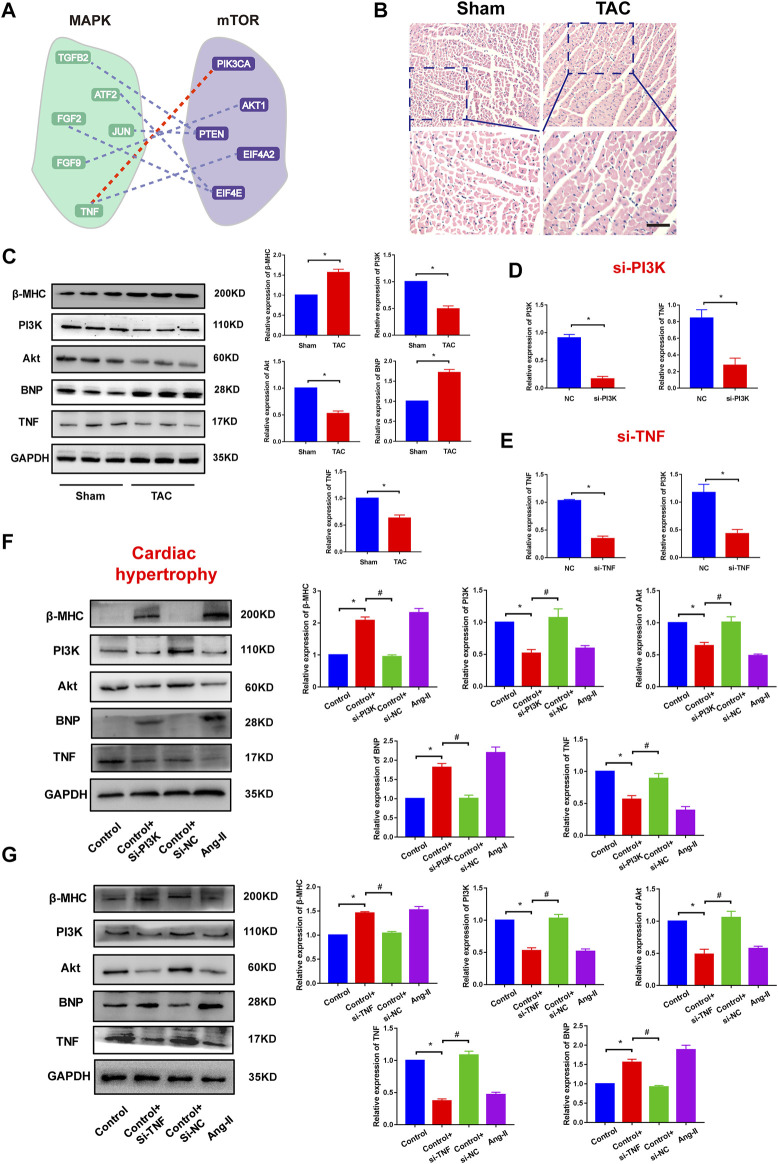
Validation of pathway crosstalk in cardiac hypertrophy. **(A)** A pathway–pathway crosstalk of common core pathway crosstalk network. Red line represents the ceRNA crosstalk that was performed in this study. **(B)** HE staining results of cardiac tissues. Bar: 100 μm. **(C)** Protein expression of hypertrophy markers, PI3K, Akt, and TNF in Sham groups and TAC groups and corresponding statistic results. *n* = 6. *p < 0.05 versus Sham group. **(D)** Real-time PCR results of PI3K and TNF expression in PI3K knockdown experiments. *n* = 6. *p < 0.05 versus. NC group. **(E)** Real-time PCR results of PI3K and TNF expression in TNF knockdown experiments. *n* = 6. *p < 0.05 versus NC group. **(F)** Protein expression of hypertrophy markers, PI3K, Akt, and TNF in PI3K knockdown experiments and corresponding statistic results. *p < 0.05 versus control group, *#p* < 0.05 versus. Control + si-PI3K group. *n* = 6. **(G)** Protein expression of hypertrophy markers, PI3K, Akt, and TNF in TNF knockdown experiments. *p < 0.05 versus control group, *#p* < 0.05 versus control + si-TNF group. *n* = 6.

We also performed biological experiments to validate the crosstalk between pathways from a common core pathway network with high topological features. For instance, the pathway–pathway crosstalk between the KEGG_MAPK_SIGNALING_PATHWAY and the BIOCARTA_MTOR_PATHWAY contained 62 ceRNA crosstalk pairs and was identified in 21 gene profiles (Figure 4A). We found that a famous ceRNA PTEN occurred in the pathway–pathway crosstalk. Many studies had verified that PTEN played pivotal roles in cardiovascular diseases (Sun et al., 2006; Oudit et al., 2008). In addition, some crucial genes were also identified from pathway–pathway crosstalk, such as PIK3CA (Lu et al., 2009) and TNF(Haudek et al., 2007). These results suggested that ceRNA-mediated core pathway–pathway crosstalk might exert similar functions in multiple CVDs. Mechanically, we constructed *in vivo* and *in vitro* cardiac hypertrophy models to investigate the crosstalk between MAPK and MTOR (Figure 4B). The results showed that PI3K and TNF were downregulated in cardiac hypertrophy (Figure 4C). The knockdown of PI3K or TNF could repress the expression of the other ceRNA (Figures 4D,E). Function experiments indicated that PI3K–TNF-mediated pathway crosstalk was the key regulator of cardiac hypertrophy (Figures 4F,G). Moreover, we also validated the PI3K–TNF-mediated pathway crosstalk in myocardial infarction models. The results showed that this pathway crosstalk also occurred in myocardial infarction (Supplementary Figure S10).

### Hub Analysis of Disease Pathway–Pathway Crosstalk Networks

**FIGURE 5:**
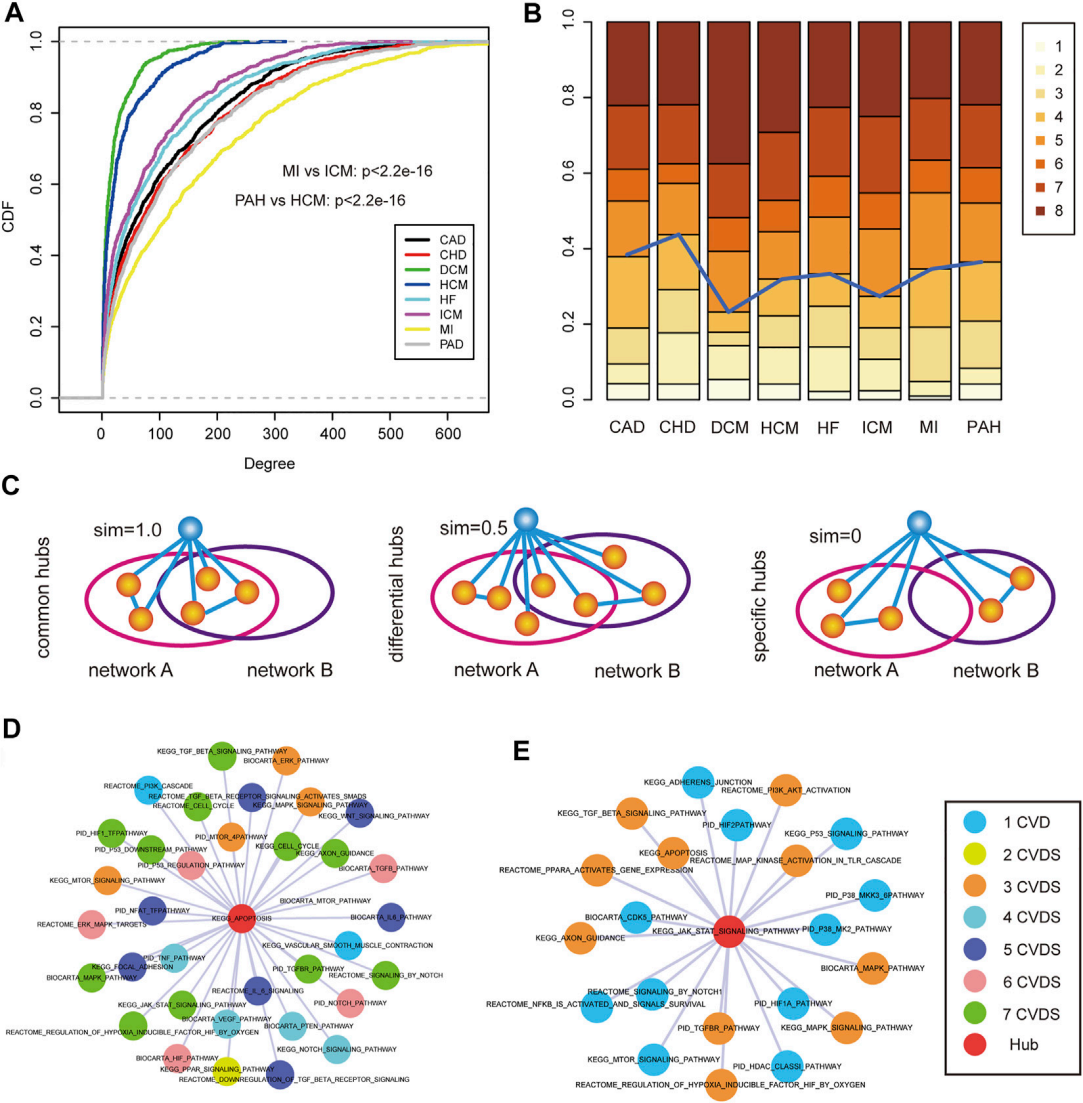
Hub identification and analysis of disease-related pathway crosstalk. **(A)** Cumulative distribution functions of the pathway degree in each CVD. **(B)** The number of hubs was distributed in 1–8 CVDs in which the hubs could occur. **(C)** Hub pathways were grouped into three categories: common hubs, differential hubs, and specific hubs. **(D)** An example of common hub pathway of “KEGG APOPTPSIS.” Colors of nodes represents the number of CVDs. **(E)** An example of differential hub pathway of “KEGG JAK STAT SIGNALING PATHWAY.”

Previous studies found that pathway–pathway crosstalk could participate in the regulation of various diseases. Next, we attempted to investigate the mechanisms of pathway–pathway crosstalk in CVDs by performing a topological analysis to pathway–pathway crosstalk networks. Firstly, 21 pathway–pathway crosstalk networks were merged into eight disease-related pathway–pathway crosstalk networks according the same disease names. Then, we compared degree cumulative distributions across CVDs and the results showed that most networks were characterized by nodes with highly variable degrees, from pathways with few links to pathways with hundreds of connections (Figure 5A). For instance, we found that the crosstalk network of PAH showed a different connectivity compared with the network of HCM (Kolmogorov–Smirnov test, *p* < 2.2e-16). In addition, the network of MI also presented a different degree distribution compared with the network of ICM (Kolmogorov–Smirnov test, *p* < 2.2e-16). These significant differences of topological features in crosstalk networks revealed that pathway–pathway crosstalk strongly impacted the mechanisms of CVDs and played important roles in cardiology.

According to the prior knowledge, hub nodes played crucial roles in biological networks, thus, we identified hub pathways from each CVD pathway–pathway crosstalk network. Interestingly, we found that most hub pathways showed a high connectivity in multiple CVD networks (Figure 5B). In order to systematically investigate the sharing of hub pathways among CVDs, we divided these hub pathways of each network into three categories (see Materials and Methods) (Figure 5C). In total, we identified 231 common hub pathways, 28 differential hub pathways, and 38 specific hub pathways. The common hub pathways were the largest components of hub pathways, suggesting the similar pathway–pathway crosstalk mechanisms across CVDs. For instance, in the common hub pathway “KEGG_APOPTOSIS,” (Figure 5D), apoptosis was a crucial process involved in the development of cardiovascular diseases (Fisher et al., 2000). We listed a part of pathway–pathway crosstalk partners of the common hub pathway, and the results showed that most crosstalk partners were encompassed in the complex regulatory mechanisms of multiple CVDs verified by many researchers, such as the MAPK pathway and the TGF-beta pathway, indicating the same mechanisms of pathway–pathway crosstalk in different CVDs. In addition, we also investigated the extent of differential hub pathway mechanisms in different CVDs. We extracted the pathway–pathway crosstalk partners of the differential hub pathway “KEGG_JAK_STAT_SIGNALING_PATHWAY” (Figure 5E) which was the hub pathway in HCM, ICM, and HF. Firstly, we observed the common partners across three CVDs of the pathway, and some shared mechanisms were identified, such as the “MAPK pathway,” “TGF-beta pathway,” and “apoptosis pathway”. As for the third category, several specific hub pathways were demonstrated to play roles in specific CVDs. The “PID_TRAIL_PATHWAY” was a hub node in the PAH network. Studies found that the anti-TRAIL antibody treatment of rodents with established PAH-reversed pulmonary vascular remodeling by reducing proliferation and inducing apoptosis, improved hemodynamic indices, and significantly increased survival (Hameed et al., 2012). Overall, these results systematically revealed the potential mechanisms of pathway–pathway crosstalk across CVDs or in a specific disease state.

### Identification of Cardiovascular Disease-Associated Pathway–Pathway Crosstalk Modules

**FIGURE 6:**
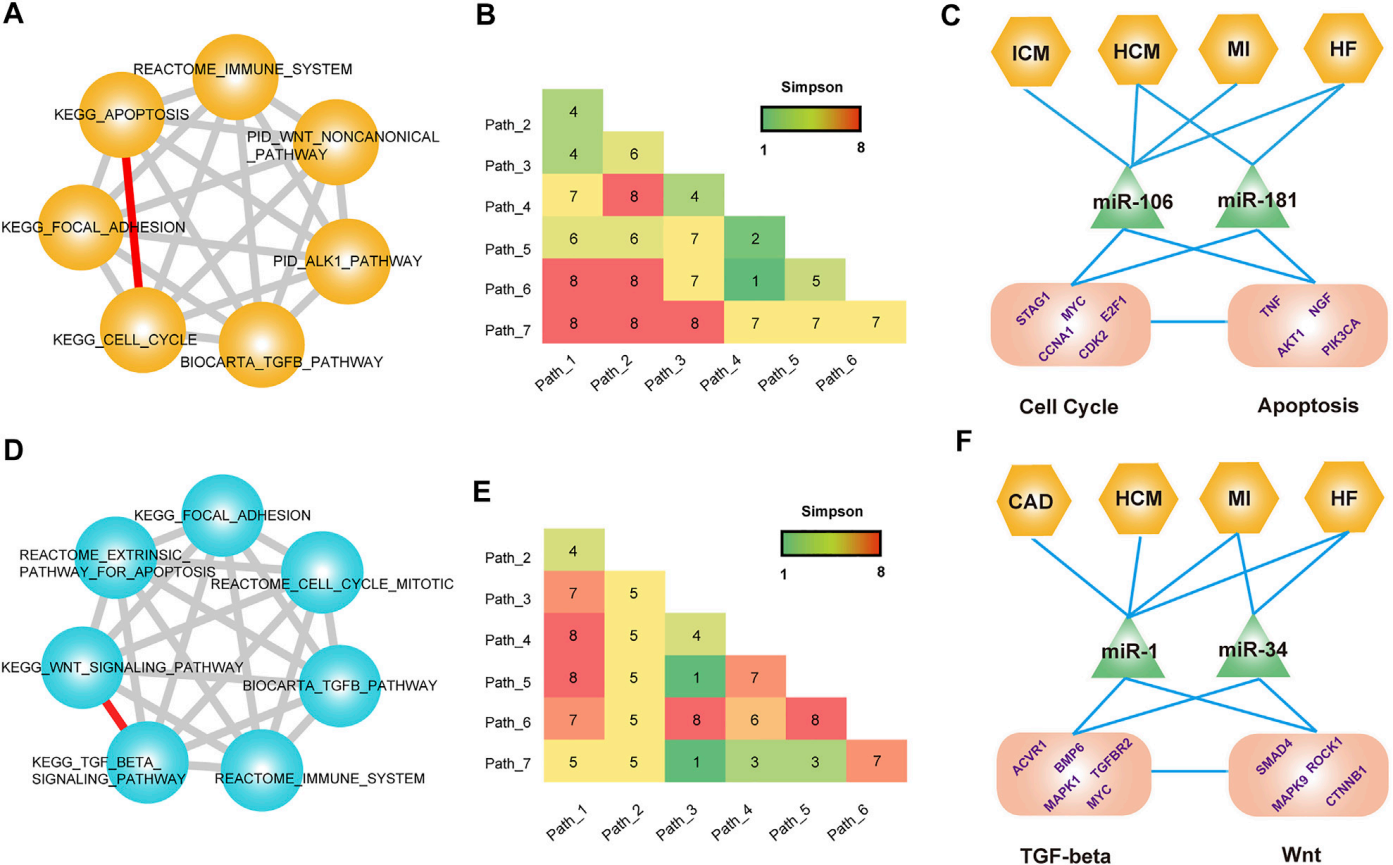
Common modules across CVDs**. (A)** A module that contained seven pathways. **(B)** Distribution matrix represents the number of CVDs occurring in each pathway crosstalk pair. **(C)** Multi-level analysis of the module. **(D–F)** Analysis of another module that contains seven pathways.

To systematically identify the CVD-associated pathway–pathway crosstalk modules, we mapped all known CVD-related pathways to each disease pathway–pathway crosstalk network. And all the sub-networks comprised of known CVD-related pathways of each disease pathway–pathway crosstalk network were merged. For the common pathway crosstalk modules, we extracted the connected component that only contained common hub pathways and differential hub pathways from the merged network. The topological analysis of the connected component showed a cluster coefficient 1,000 times higher than that of random networks (Supplementary Figure S11). These results indicated that known CVD-related pathways showed a higher connectivity in CVD pathway–pathway crosstalk networks. Next, we performed a module-identification algorithm on the connected components to identify the common modules that exerted specific functions in CVDs. As a result, 483 cliques were identified as CVD-associated common pathway crosstalk modules (Supplementary Table S2 **)**. A multi-level analysis of these modules revealed that most pathways were regulated by disease-related genes and miRNAs. For example, a common module encompassed 21 pathway–pathway crosstalk among seven hub pathways (Figures 6A,B). Most of the seven pathways were demonstrated to participate in diverse CVDs, such as cell cycle (Ahuja et al., 2007), apoptosis (Haunstetter and Izumo, 1998), and the TGF-beta pathway (Ikeuchi et al., 2004). Previous studies had found regulatory mechanisms between cell cycle arrest and apoptosis in multiple diseases (Penttinen et al., 2005; Tozluoğlu et al., 2008), thus we suspected the ceRNA crosstalk between pathways induced the pathway interactions. We displayed a mimic diagram of the subset of ceRNA crosstalk pairs between two important CVD-related pathways: KEGG_CELL_CYCLE and KEGG_APOPTOSIS (Figure 6C and Supplementary Figure S12). We extracted all the ceRNA crosstalk pairs that sustained the pathway–pathway crosstalk. Furthermore, the miRNAs that mediated ceRNA crosstalks were also extracted and mapped to CVDs. Interestingly, we found that some ceRNAs played crucial roles in the biological processes of CVDs that had been demonstrated by previous studies. Lee *et al*. found that the induction of Myc in cardiomyocytes was sufficient to cause cardiomyopathy and heart failure, and sustained induction of Myc could lead to cell cycle re-entry in adult cardiomyocytes, representing a maladaptive response for the mature heart (Lee *et al*., 2009). Wang *et al*. identified that E2F1 transcriptionally repressed miR-30 b expression. The knockdown of E2F1 in cardiomyocytes inhibited necrotic cell death, and E2F1 knockout mice showed reduced necrosis and myocardial infarct size (Wang et al., 2015). The TNF also participated in biological processes of numerous CVDs (Kleinbongard et al., 2010). In addition, we also investigated all the miRNAs that mediated ceRNA crosstalk between two pathways. The results showed that some known disease miRNAs had been extracted. Guan *et al*. found that miR-106a was an important factor to promote hypertrophic progress, and suggested miR-106a as a new molecular target for the treatment of pathological hypertrophy (Guan et al., 2016). Moreover, studies found that the miR-181 family could regulate cellular apoptosis, which exerted crucial roles in multi-CVDs (Fisher et al., 2000; Ouyang et al., 2012).

Another example was the module that contained 21 pathway–pathway crosstalk pairs among seven pathways (Figures 6D,E). Similarly, this module also covered multiple known CVD-related pathways. We emphatically analyzed the pathway–pathway crosstalk between KEGG_TGF_BETA_SIGNALING_PATHWAY and KEGG_WNT_SIGNALING_PATHWAY (Figure 5F and Supplementary Figure S13). Some studies had verified the crosstalk activity between the TGF-beta pathway and the WNT signaling pathway (Nishita et al., 2000; Attisano and Labbé, 2004). For the transcriptional-level analysis of the module, we also observed the known CVD-related ceRNA pairs and miRNAs that mediated ceRNA crosstalk. MAPK1 was also named ERK, involved in a broad range of CVD-related biological processes (Darling et al., 2005; Purcell et al., 2007). Researchers found that an upregulation of MEK1–ERK1/2 signaling as a consequence of Smad4 deletion underlies the impaired cardiac function and revealed an important function of Smad4 in cardiac remodeling (Wang et al., 2005). Additionally, miR-1 and miR-34 were both key participants in complex cardiology regulatory processes. Summarily, these common modules indicated the crucial regulatory roles of pathway–pathway crosstalk across CVDs and revealed the potential mechanisms of pathway–pathway crosstalk in systemic biology.

### Random Walk With Restart to Network Revealed Potentially Novel Cardiovascular Disease-Related Pathways

To more effectively identify novel CVD-related pathways from global pathway–pathway crosstalk, we performed a random walk algorithm on a hub-induced sub-network, which had been constructed by merging all hub pathway–hub pathway pairs from each disease pathway network (see in Materials and Methods). We mapped all the known CVD-related pathways into the hub-induced sub-network as the seed nodes. After 5,000 times shuffling, 23 hub pathways were identified as the potentially novel CVD-related pathways (Supplementary Table S3), suggesting that these hub pathways connected seed nodes closely in crosstalk networks and might play a role in the CVD-related biological processes. For instance, the pathway “BIOCARTA_FAS_PATHWAY” that ranks in the top of the results had been demonstrated to play a key role in apoptosis and cell death (Wajant, 2002). Specifically, Lee *et al*. found that the activation of Fas could induce apoptosis in cardiac myocytes, and Fas was a critical mediator of MI because of ischemia–reperfusion (Lee et al., 2003). Moreover, the blockade of apoptosis by interfering with the Fas/Fas ligand interaction might become one of the therapeutic strategies against chronic heart failure after MI (Li et al., 2004). In addition, we also identified the pathway “PID_E2F_PATHWAY.” The E2F family encompassed a subset of crucial transcription factors, and had been verified that they are involved in many CVD-related biological processes (Vara et al., 2003; Wolfram et al., 2011).

### Database of Competing Endogenous RNA-Mediated Pathway–Pathway Crosstalk of Cardiovascular Diseases

**FIGURE 7:**
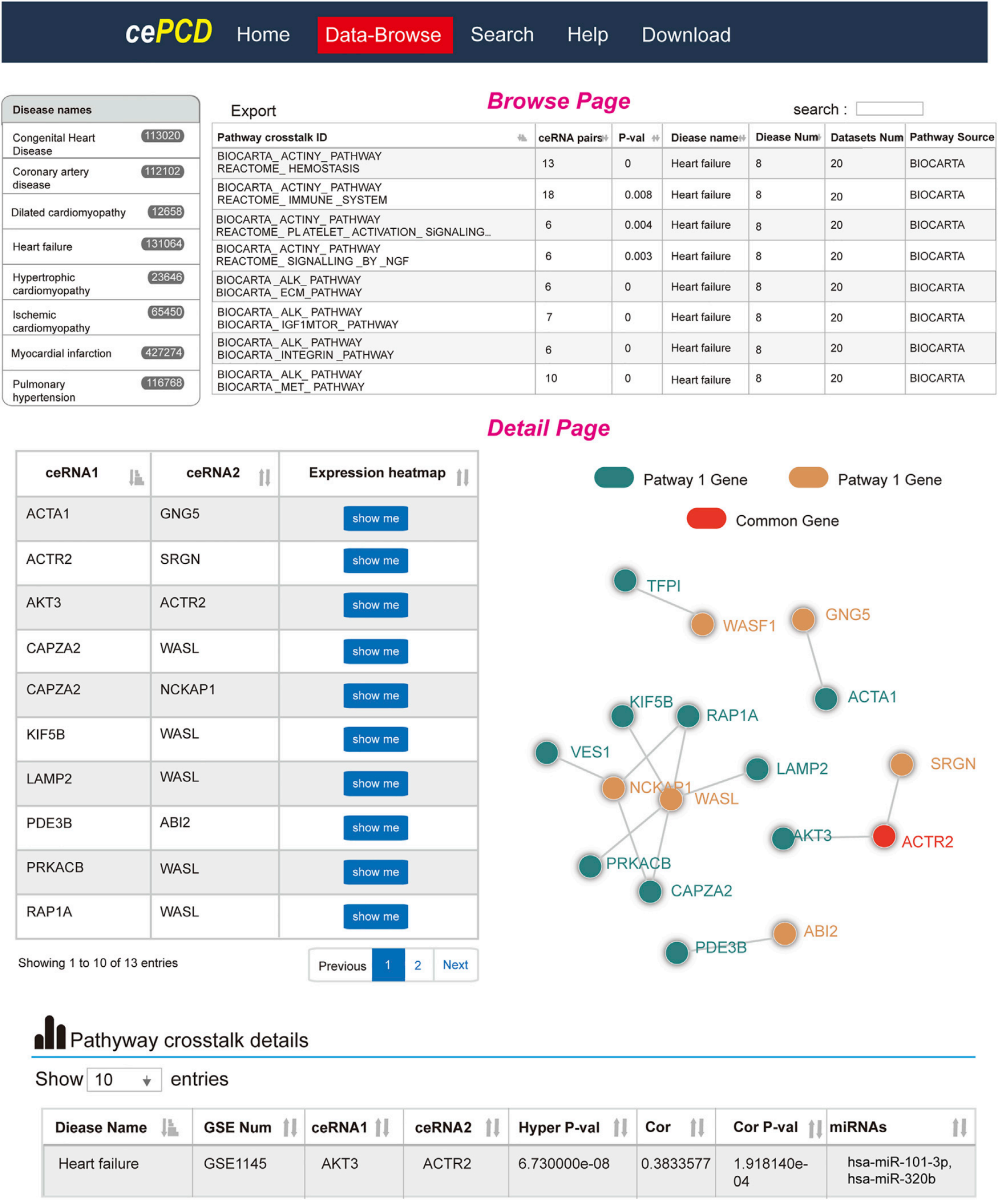
Website design of cePCD, which provides comprehensive annotations for pathway crosstalk in CVDs.

To facilitate the use of ceRNA-mediated pathway–pathway crosstalk of CVDs, we also developed a friendly web source using PHP at http://www.licpathway.net/cepathway/index.html (Figure 7). Users could browse or search pathways of interest from the web tools. We provided comprehensive information for pathway–pathway crosstalk, such as ceRNA pairs, Pearson correlation coefficients, miRNA mediators, and regulatory networks. Briefly, cePCD offers browse, search, and download function for users. In the browse page, users can quickly search pathway crosstalk by “disease names,” “GSE number,” and “data source.” In the interactive result table, users can find the detailed information of each pathway crosstalk. cePCD provides the ceRNA pair numbers, permutation *p* values, disease numbers, dataset numbers, and pathway sources information (Figure 7, top). Users can click the “pathway crosstalk ID” to view the comprehensive annotation information of pathway ID. In the pathway crosstalk detailed page, cePCD provides a detailed ceRNA crosstalk that mediates pathway crosstalk, such as ceRNA expression heatmap and crosstalk network visualization (Figure 7, bottom). Furthermore, cePCD also provides annotation information for ceRNA crosstalk. Users can view the strength (Pearson correlation coefficients and hypergeometric test significances) and regulators (miRNAs) of ceRNA crosstalk in the interactive tables. cePCD provides three types of search for users as follows: “search pathways by disease,” “search pathways by gene,” and “search pathways by data source.” cePCD will respond quickly and display the search contents. In the result table, users can view the detailed information as “browse” page. cePCD provides two types of results for users to download. Users can download pathway–pathway interaction results and ceRNA crosstalk in 21 datasets from this page.

## Discussion 

In this study, we systematically analyzed the landscape of ceRNA-mediated pathway–pathway crosstalk across eight major CVDs *via* construction and analysis of pathway–pathway crosstalk networks. As a result, we found some conserved features across CVDs. For instance, all the degree distributions of pathway–pathway crosstalk networks were scale free and followed a power law distribution. The pathways with a high connectivity in networks showed a high crosstalk activity and contained more genes. Additionally, after the comparison of pathway–pathway interactions of each pathway–pathway crosstalk network, results showed that a fraction of pathway–pathway crosstalk was conserved in multiple CVDs and a core pathway–pathway crosstalk network was identified, indicating that there was a stable framework-encompassed pathway–pathway crosstalk, which might play a basic role in the mechanisms of cardiology. Hub nodes were more essential than other nodes in biological networks, thus we split all the hub pathways of each pathway–pathway crosstalk network into three categories: common hubs, differential hubs, and specific hubs, which could reveal the common or specific biological mechanisms in-depth analysis of networks. Furthermore, we mapped all common hub pathways, differential hub pathways, and known CVD-related pathways into a merged global disease pathway–pathway crosstalk network and extracted the sub-network among them. Importantly, after performing the CFinder algorithm on the sub-network, ∼480 common modules were identified, indicating that these hubs linked closely in the network and might exert their regulatory functions in high coordination. Moreover, we performed a random walk in the pathway–pathway crosstalk network and identified a subset of pathways that were highly related to CVD.

Pathway crosstalk had been proposed for some years, which were demonstrated to play crucial roles in biological regulatory mechanisms of multiple diseases (Attisano and Labbé, 2004; Javelaud and Mauviel, 2005; Guo and Wang, 2009). Previous studies had proposed many strategies to investigate the pathway–pathway crosstalk, such as gene overlap method, gene expression based-method, biological network based-method, and statistical model method (Li et al., 2008; Mccormack et al., 2013; Wang et al., 2013; Ogris et al., 2016). Most of these methods ignored the biological relationships of the genes in different pathways. Here, we firstly proposed a novel pathway crosstalk mechanism, which was supported by ceRNA crosstalk. CeRNA crosstalk had attracted broad attention because of its crucial regulatory roles in multiple diseases, from cancer to CVD. In our previous study, we had investigated the CVD-related ceRNA crosstalk in eight types of cardiovascular diseases. Interestingly, we found that ceRNA crosstalk were high related with pathway crosstalk. Briefly, we defined two types of ceRNA-mediated pathway crosstalk, including ceRNA pairs within pathways (CPWP) and ceRNA pairs between pathways (CPBP), and results showed that CVD-related ceRNA crosstalk were mainly distributed in CPBP. Results showed that ceRNA-mediated pathway crosstalk have the strong regulatory potentials (Song et al., 2017). This result inspired us to comprehensively investigate the ceRNA-mediated pathway crosstalk in CVDs. Hence, the important novelty of the study was the novel pathway crosstalk mechanism, which could fill the gap of the previous pathway crosstalk identification methods and provide a new way to investigate the biological meanings in pathway crosstalk perspective.

Here, we proposed a novel method to identify pathway–pathway crosstalk by measuring the statistical significance of CVD-related ceRNA crosstalk between pathways. Compared with previous biological network-based methods, our method focused on the ceRNA crosstalk and identified more significant pathway–pathway crosstalk that encompassed more ceRNA pairs. Furthermore, from this study, we identified some pathway drivers in CVDs, such as MAPK and mTOR signal pathways. Biological experiments also demonstrated that ceRNA crosstalk between pathways had the effects on the pathway crosstalk and pathological processes.

Pathway crosstalks are considered as key regulatory mechanisms in multiple diseases, from cancer to CVDs (Nishida et al., 2008; Maniati et al., 2011). A systematic analysis of pathway–pathway crosstalk enabled us to elucidate the regulatory processes of ceRNA crosstalk. Importantly, we identified ∼480 common modules by integrating the analysis of non-specific hub pathways in the network and known CVD-related pathways. For instance, we identified the crosstalk between the TGF-beta signaling pathway and WNT pathway in multiple modules. Many studies had revealed the crosstalk activity between the two pathways in multiple biology processes, such as differentiation and tumorigenesis (Attisano and Labbé, 2004). In our study, we extracted the ceRNA pairs between the TGF-beta signaling pathway and the WNTt pathway and some ceRNAs are shown in Figure 5. For the ceRNA pair SMAD4-LEF1, a previous study had verified that SMAD4 could cooperate with LEF1 to activate the famous oncogene c-myc, which was a key regulator of cell proliferation (Lim and Hoffmann, 2006). Additionally, for the ceRNA crosstalk pair MAPK1–CCNNB1, studies found that EGF-induced activation of ERK (MAPK1) could promote β-catenin (CCNB1) transactivation and tumor cell invasion (Ji et al., 2009). In the field of CVDs, studies also demonstrated the crucial roles of the two pathways in cardiology. Thus, we considered that the activity of ceRNA crosstalk had a contribution to the pathway–pathway crosstalk. Moreover, we performed a random walk on the pathway–pathway crosstalk network and 23 pathways were identified as CVD-related pathways after 5,000 times permutation. Interestingly, some pathways had been demonstrated to play a role in cardiology, such as “BIOCARTA_FAS _PATHWAY,” “PID_E2F_PATHWAY,” and “BIOCARTA_MAPK_PATHWAY” (Tanaka et al., 2001; Lee et al., 2003; Wolfram et al., 2011). In summary, all these results showed the important roles of ceRNA-mediated pathway–pathway crosstalk in cardiology and motivate us to develop a ceRNA-based pathway enrichment tools in later studies.

However, there were also some limitations in our study. For instance, we used the pathway data from four unique pathway databases that curated in MSigDB. However, only ∼7,000 genes were annotated in these pathways; with the development of biology, we considered that a scale with more pathway annotations could provide a more stable pathway–pathway crosstalk result. Secondly, in the field of CVDs, there was no established database like TCGA to investigate molecular interactions till now. We downloaded the CVD-related gene expression data from the GEO database, but these data came from multiple platforms and different samples, which might produce false-positives, although we also found some common features in these data. In addition, we used a popular strategy to identify ceRNA crosstalk, if a more accurate method can be proposed to identify ceRNA crosstalk, we believe that our results will be more stable. Finally, in the current version of cePCD, we only focused on displaying the ceRNA-mediated pathway crosstalk and ignored the functional annotation or enrichment for the ceRNAs that were involved in pathway crosstalk. We will update our database to embed more analysis tools in the next version, such as gene set enrichment analysis, gene ontology analysis, and pathway analysis.

In conclusion, we performed a global view to investigate the ceRNA-mediated pathway–pathway crosstalk in eight major CVDs. All results suggested that ceRNA-mediated pathway–pathway crosstalk participated in CVD-related biological progresses broadly. Importantly, we identified a lot of common modules, which were formed by hub pathway nodes in the networks and considered as crucial regulatory structures. Furthermore, some pathways were identified as novel CVD-related pathways by a random walk algorithm. In total, our results revealed the potential molecular regulatory mechanisms of ceRNA crosstalk in pathway–pathway crosstalk levels and provided a novel routine to investigate the pathway–pathway crosstalk of cardiology.

## Data Availability

The original contributions presented in the study are included in the article/Supplementary Material, further inquiries can be directed to the corresponding authors.
