# Supplementary material for: Characterization and Validation of ceRNA-Mediated Pathway–Pathway Crosstalk Networks Across Eight Major Cardiovascular Diseases
Source: Front Cell Dev Biol. 2022 Apr 1;10:762129. doi: 10.3389/fcell.2022.762129 (PMC9010821; doi:10.3389/fcell.2022.762129)
Supplement: Supplementary file 1 [file Table4.DOC]

**Supplementary materials of "The landscape of ceRNA mediated pathway-pathway crosstalk networks across 8 major CVDs"**

**Supplementary materials and methods**

**Protein-coding gene expression profiles of major cardiovascular diseases**

All protein-coding gene expression profiles were downloaded from Gene Expression Omnibus (GEO, http://www.ncbi.nlm.nih.gov/geo/). GEO is a public functional genomics data repository supporting MIAME-compliant data submissions. In total, our study investigated 8 major CVDs including coronary artery disease(CAD), hypertrophic cardiomyopathy (HCM), dilated cardiomyopathy (DCM), ischemic cardiomyopathy (ICM), heart failure (HF), myocardial infarction (MI), pulmonary hypertension (PAH) and congenital heart disease (CHD) within 21 gene expression datasets (detailed description see in Supplementary Table S1). As for the profiles with raw expression values, we performed log2 transformed to the gene expression values for the subsequent analysis.

**CLIP-seq-supported miRNA-mRNA interactions**

Crosslinking and Argonaute (Ago) immunoprecipitation coupled with high-throughput sequencing (CLIP-Seq) could identify the genome-wide interaction of miRNAs and their target-RNAs. In this study, we obtained the CLIP-supported interaction data from starBase V2.0, which designed for decoding interaction network via integrating large-scale CLIP-Seq (HITS-CLIP, PAR-CLIP, iCLIP, CLASH) data. To investigate the human miRNA-mRNA interactions, miRNA targets were predicted by five target predicted algorithms, including TargetScan, miRanda, Pictar, PITA, and RNA22. In total, we had downloaded 423,975 miRNA-mRNA interactions that contained 386 miRNAs and 13,802 mRNAs.

**Construction of CVD related ceRNA network**

We had two principles to identify the ceRNA pairs in each profile. On the one hand, the activity of ceRNA cross-talk increased as the number of shared miRNAs between one pair ceRNA increased. To do it, we listed all candidate mRNA-mRNA pairs from miRNA-mRNA interactions, which were obtained from the CLIP-seq-supported miRNA-mRNA interacted relations obtained from starBase and then computed the number of shared miRNAs of each candidate mRNA-mRNA pairs. We removed the pairs with less than 3 common miRNAs. A hypergeometric test was performed to estimate the significance of each pair mRNA-mRNA relation that shared common miRNAs. The p-value was measured as:

$p-value=1-\sum_{i=0}^{r-1} \frac{\left(\begin{matrix} t \\ i \end{matrix}\right)\left(\begin{matrix} m-t \\ n-i \end{matrix}\right)}{\left(\begin{matrix} m \\ n \end{matrix}\right)}$

where, m represented total number of human genome miRNA, t represented number of miRNA interacting with the mRNA1, n represented number of miRNA interacting with the mRNA2, and r represented number of miRNA shared between mRNA1 and mRNA2. All the p-values that computed by hypergeometric test were adjusted by the method induced by Holm. On the other hand, based on the mechanism of ceRNA, over-expression of one ceRNA could result in the increased expression of the other one ceRNA in one ceRNA cross-talk. This suggested that the expression of ceRNA pairs was positively correlated. Then we computed the Pearson correlation coefficient of the candidate ceRNA pairs in each of 21 CVD related gene expression profile. All the candidate ceRNA pairs with adjusted p-value < 0.01 from hypergeometric test and R > 0, p-value < 0.01 from Pearson correlation coefficient test were identified as ceRNA–ceRNA interactions. The ceRNA network of each cardiovascular disease was generated by merging all ceRNA–ceRNA interactions of each gene expression profile. In the networks, nodes represented mRNAs and edges represented co-expression ceRNA pairs that regulated through miRNAs. All these networks were visualized by Cytoscape.

**
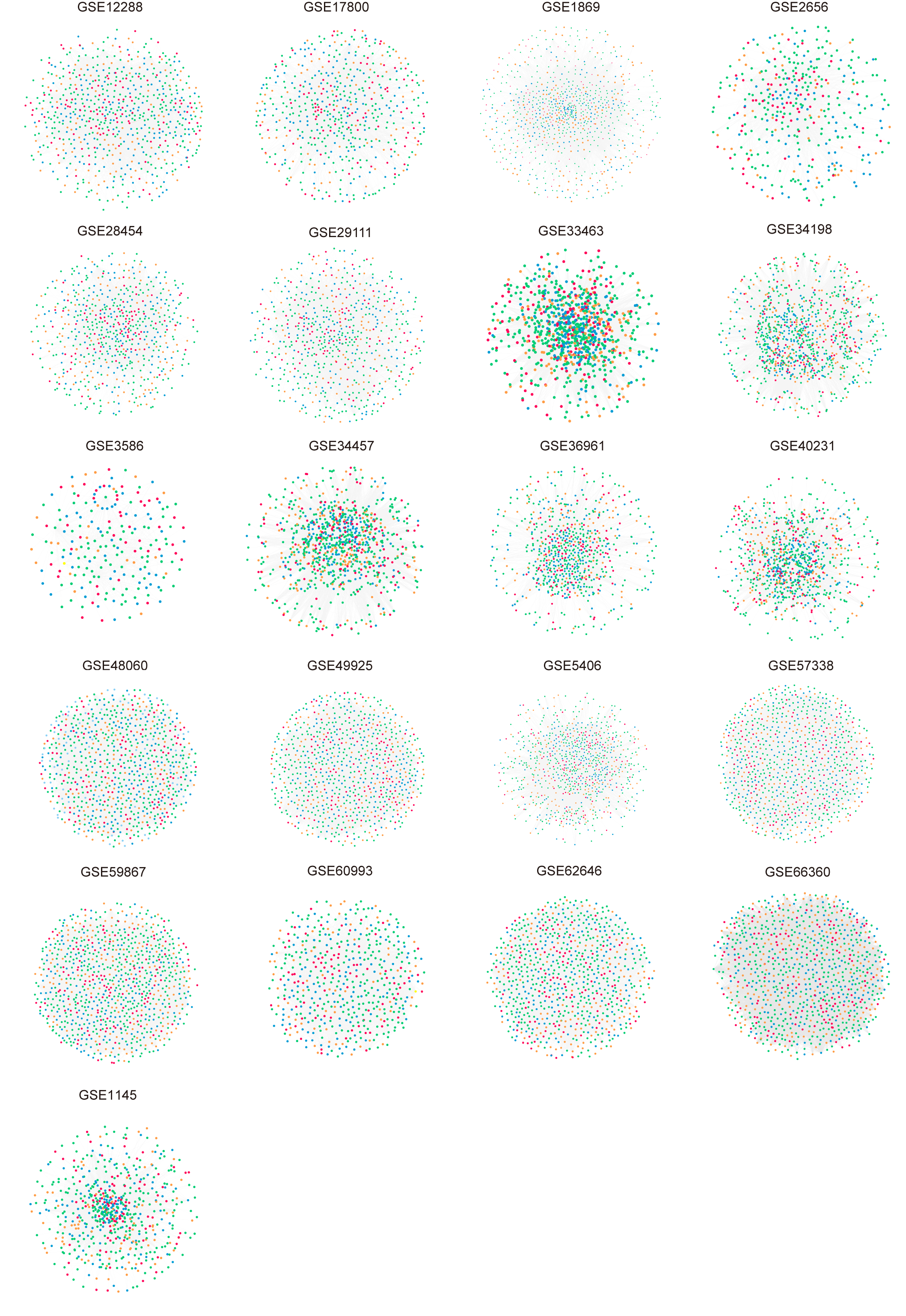
**

**Supplementary Figure S1: The view of ceRNA mediated pathway crosstalk networks**. In this study, ­we constructed 21 pathway crosstalk networks and networks were viewed by Cytoscape.


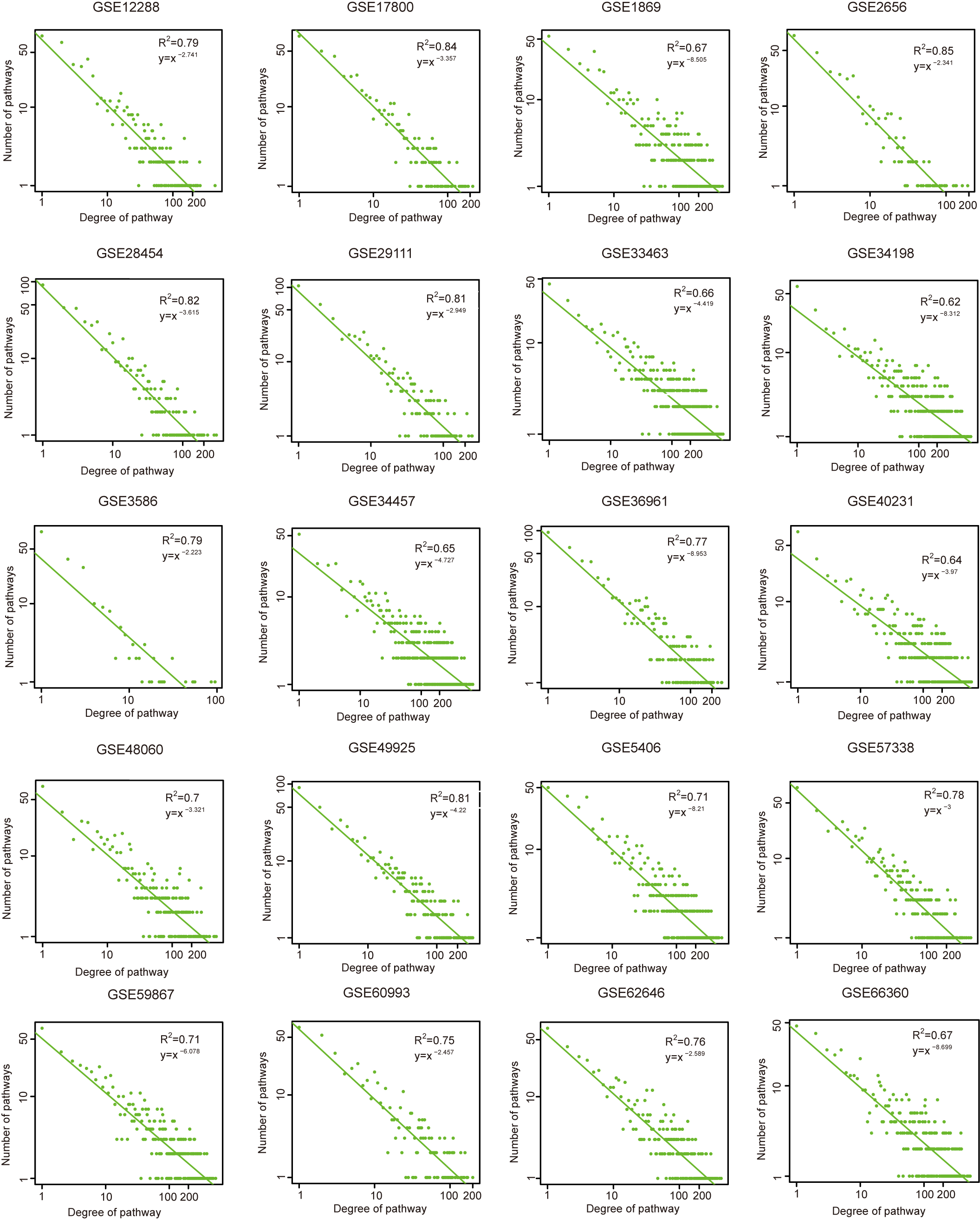


**Supplementary Figure S2: Degree distributions of crosstalk networks**. All networks were followed power-law distributions.

**
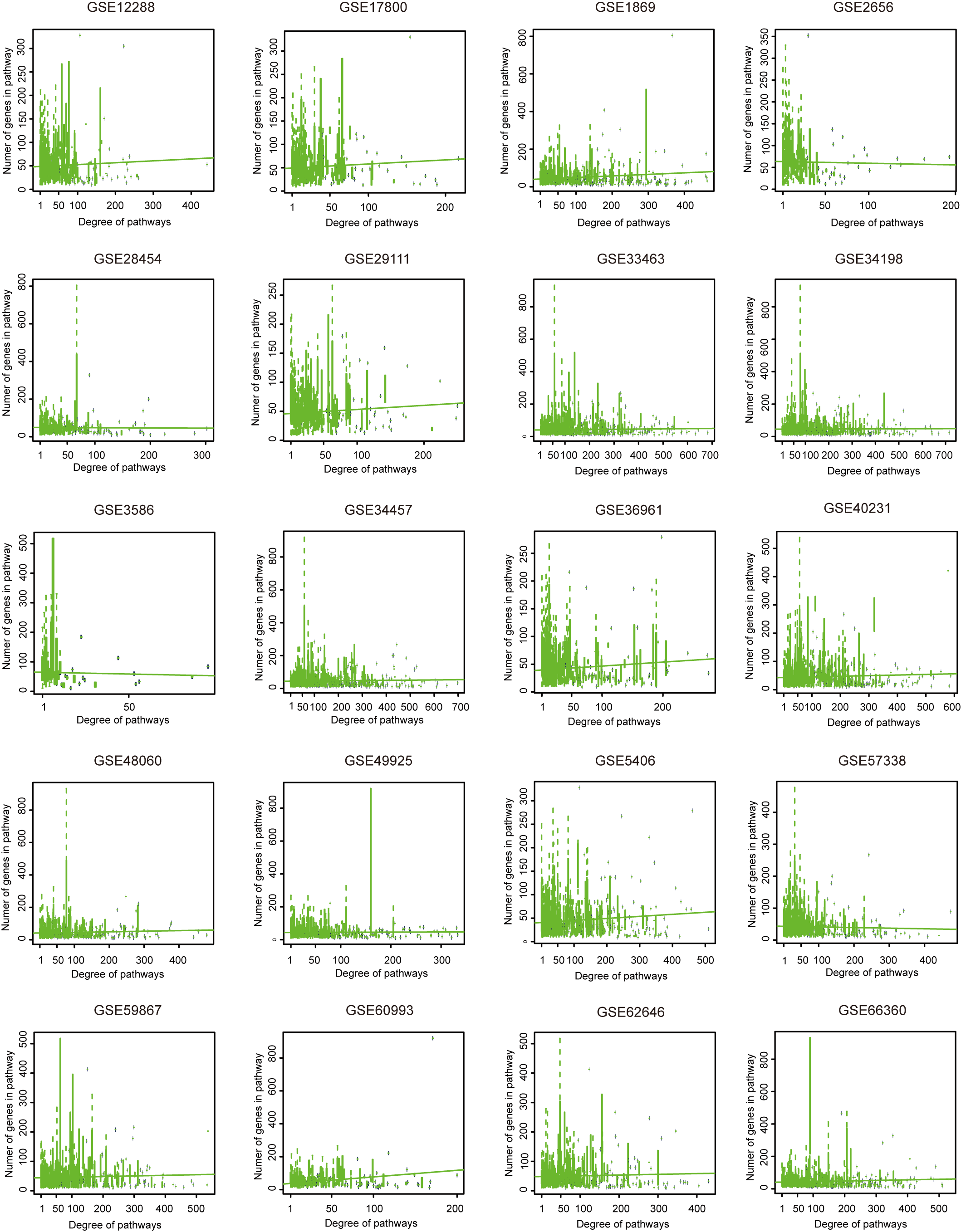
**

**Supplementary Figure S3: Pathways with high degree encompassed more genes than others**.


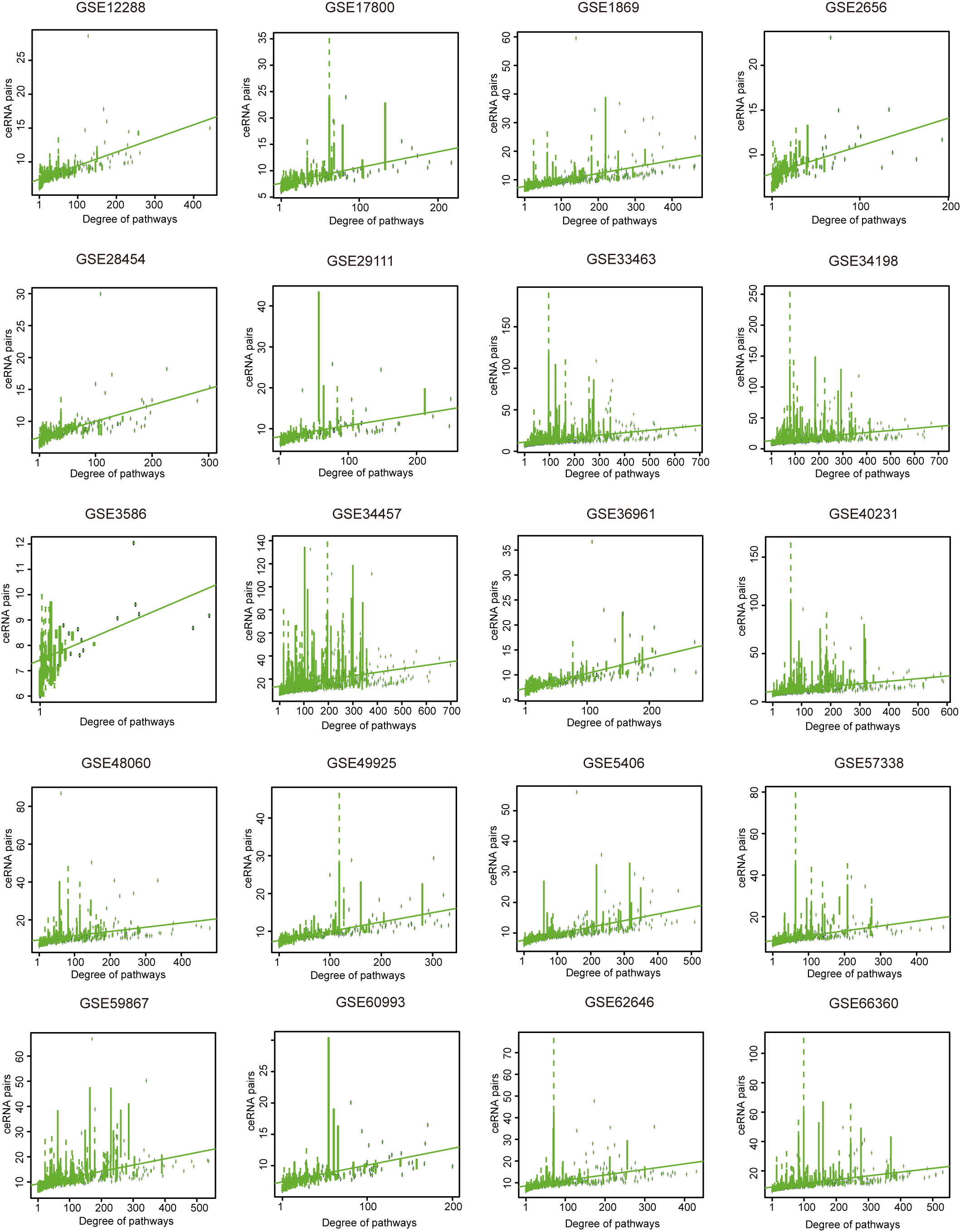


**Supplementary Figure S4: Pathways with high degree occupied more ceRNA pairs than others**.


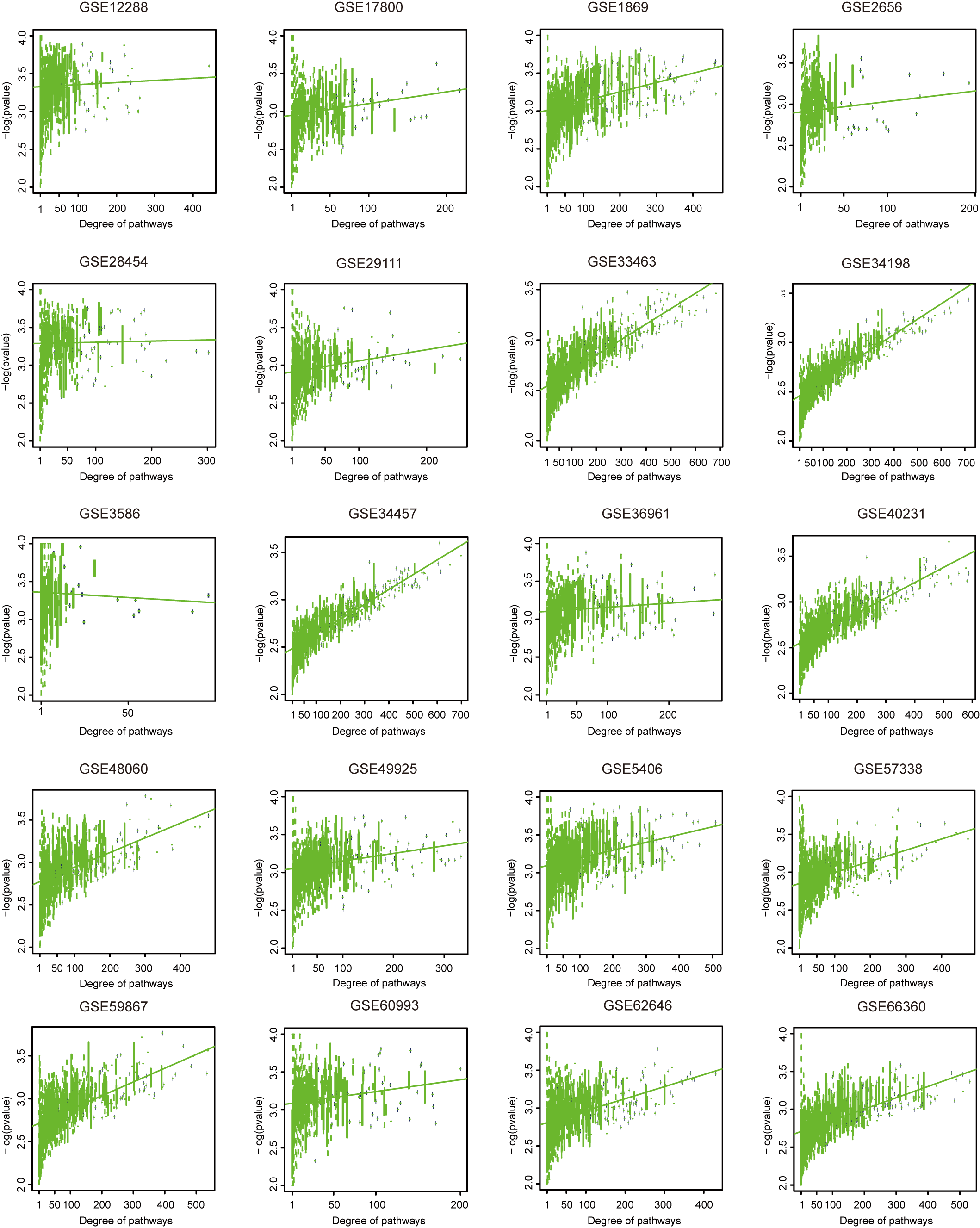


**Supplementary Figure S5: Pathways with high degree have more crosstalk activities than others**.


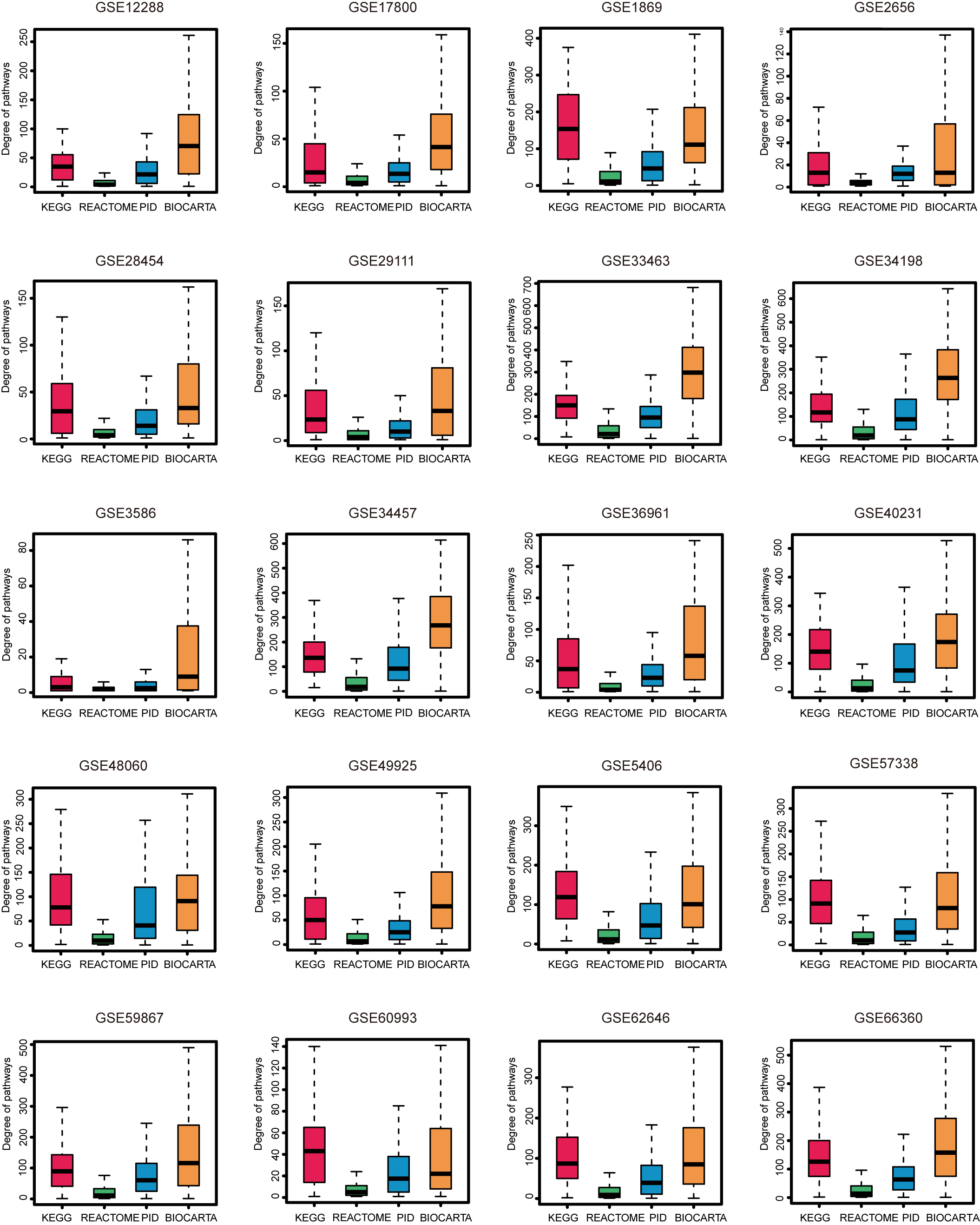


**Supplementary Figure S6: Degree distributions of four pathway databases**.


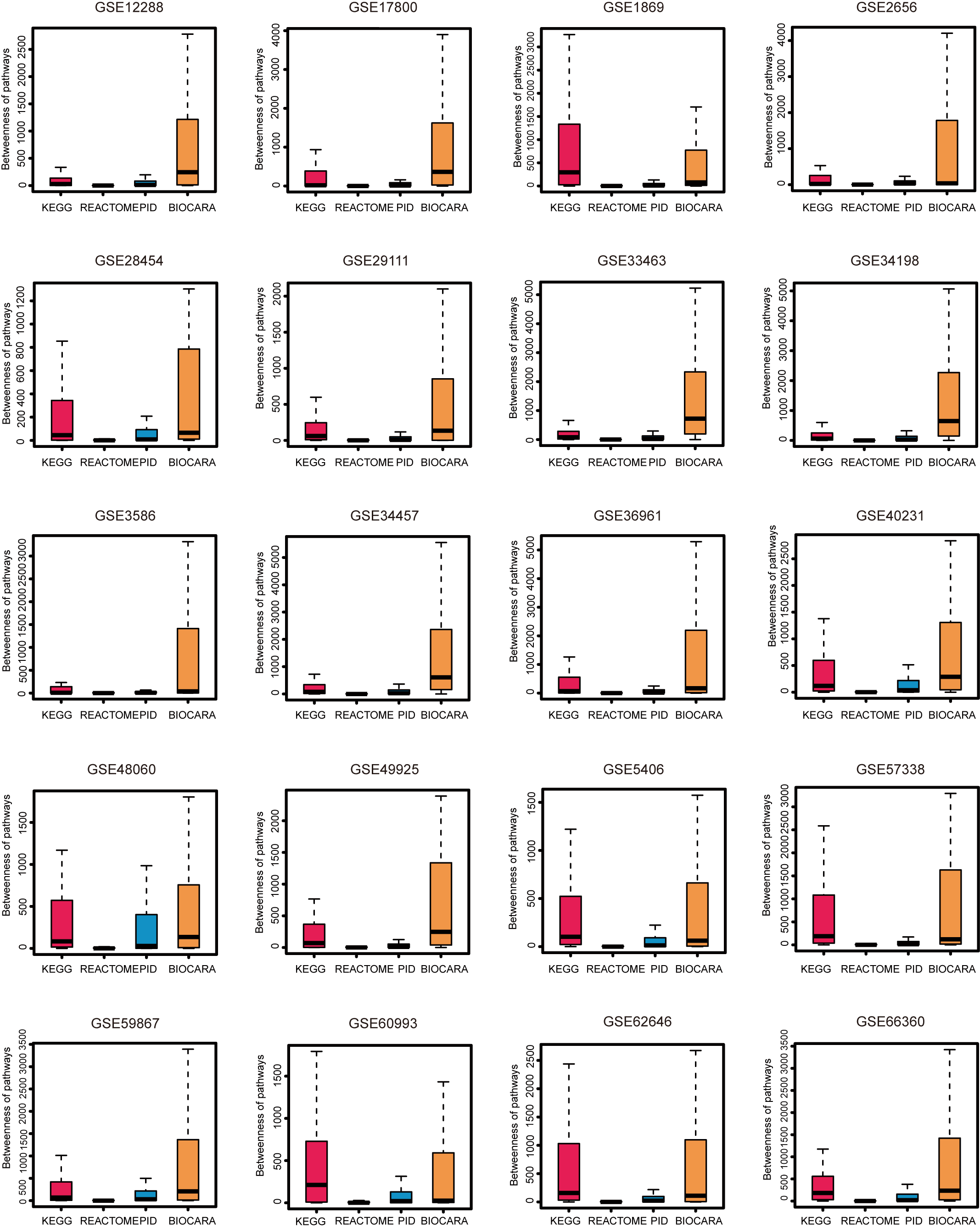


**Supplementary Figure S7: Betweenness distributions of four pathway databases**.


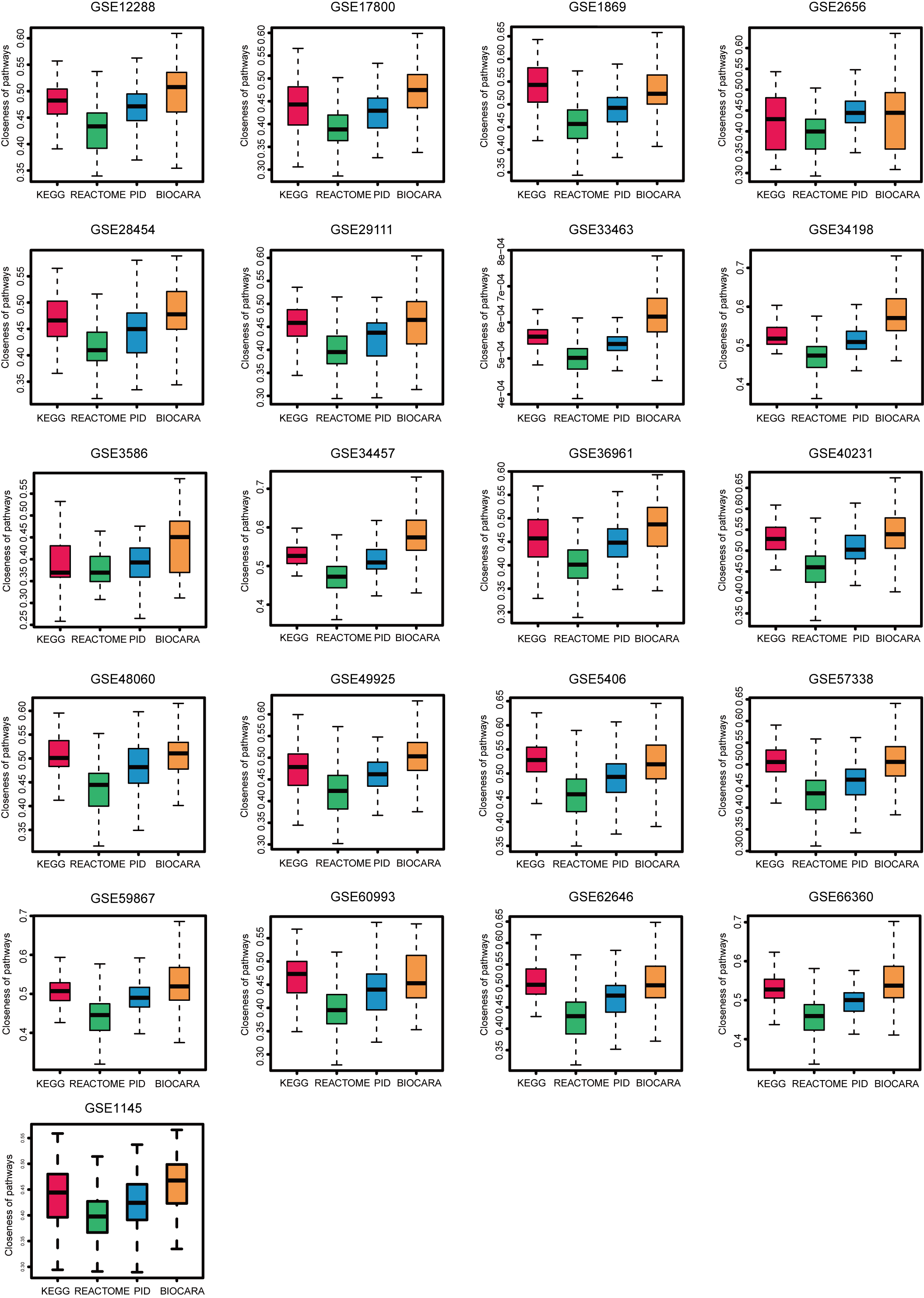


**Supplementary Figure S8: Closeness distributions of four pathway databases**.

**
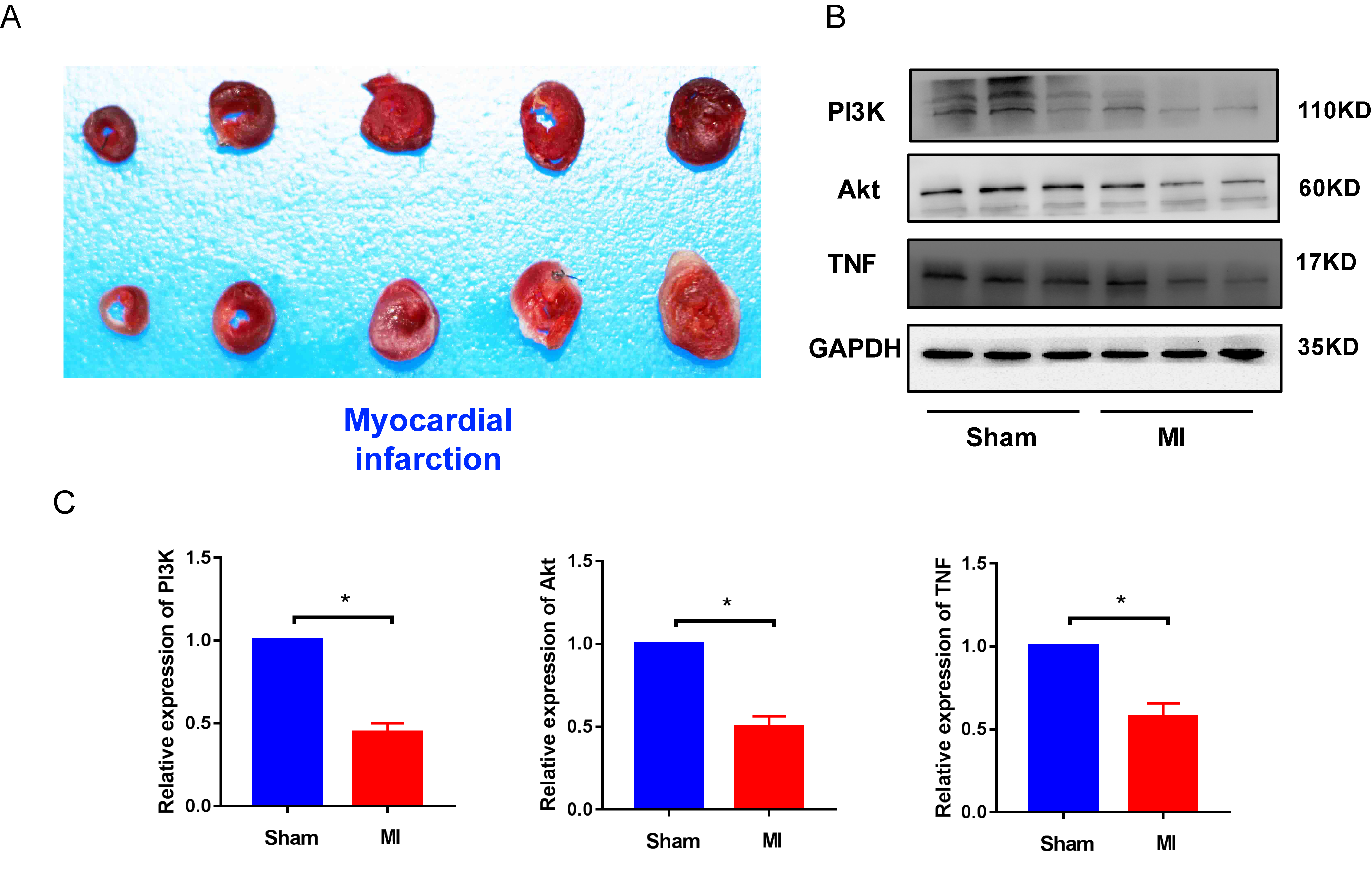
**

**Supplementary Figure S9: Validation of pathway crosstalks in myocardial infarction**.

**
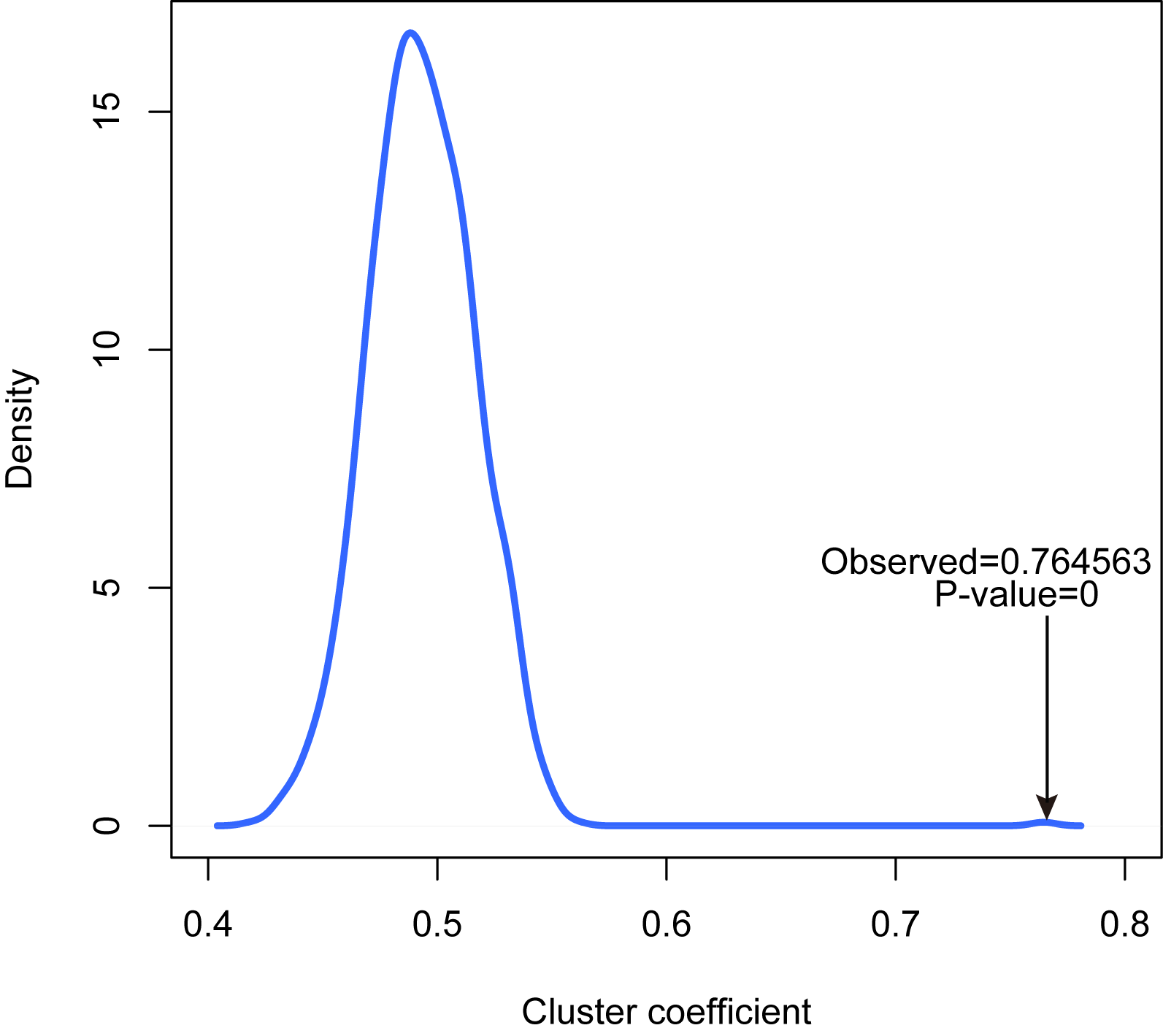
**

**Supplementary Figure S10: The plot of 1000 times network shuffling**.

**
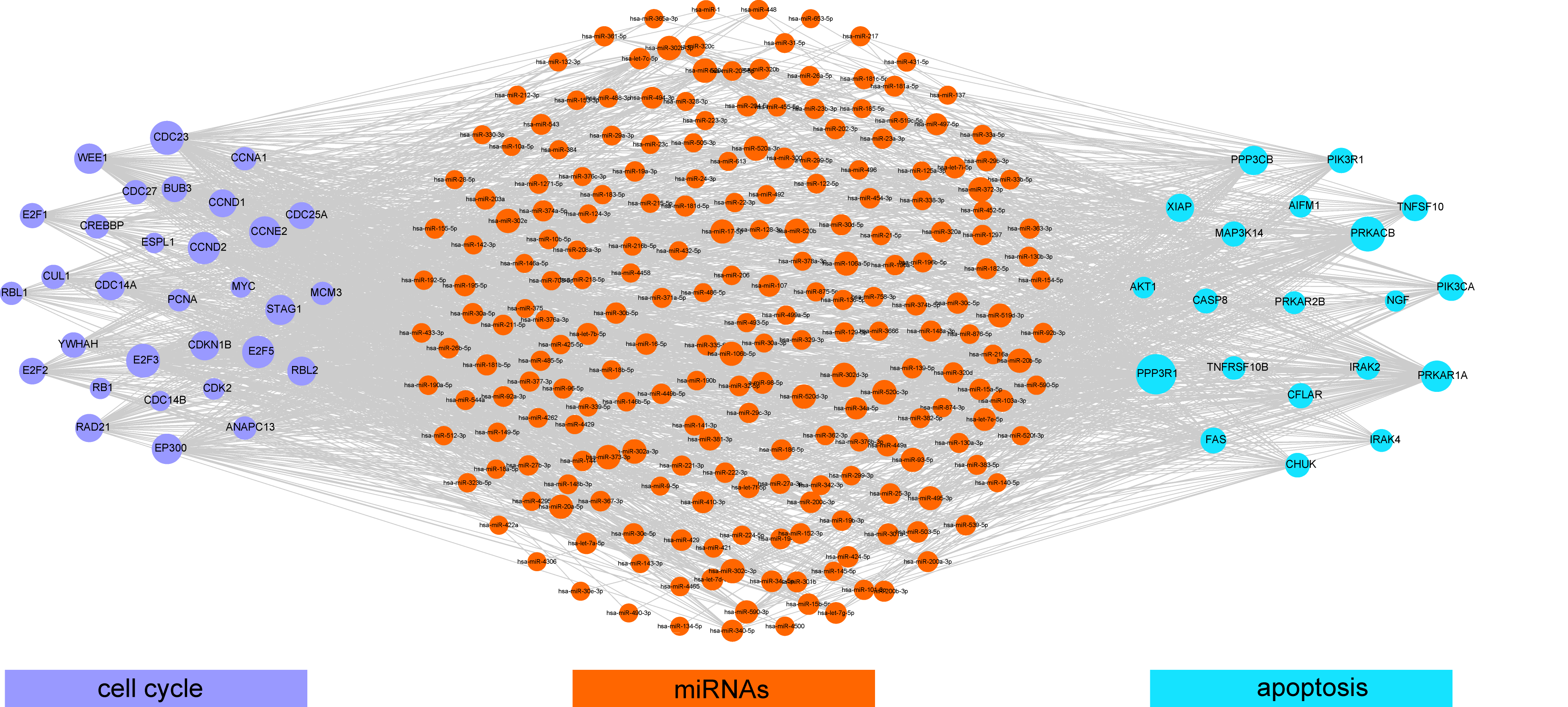
**

**Supplementary Figure S11: The comprehensive ceRNA crosstalks between cell cycle and apoptosis.**

**
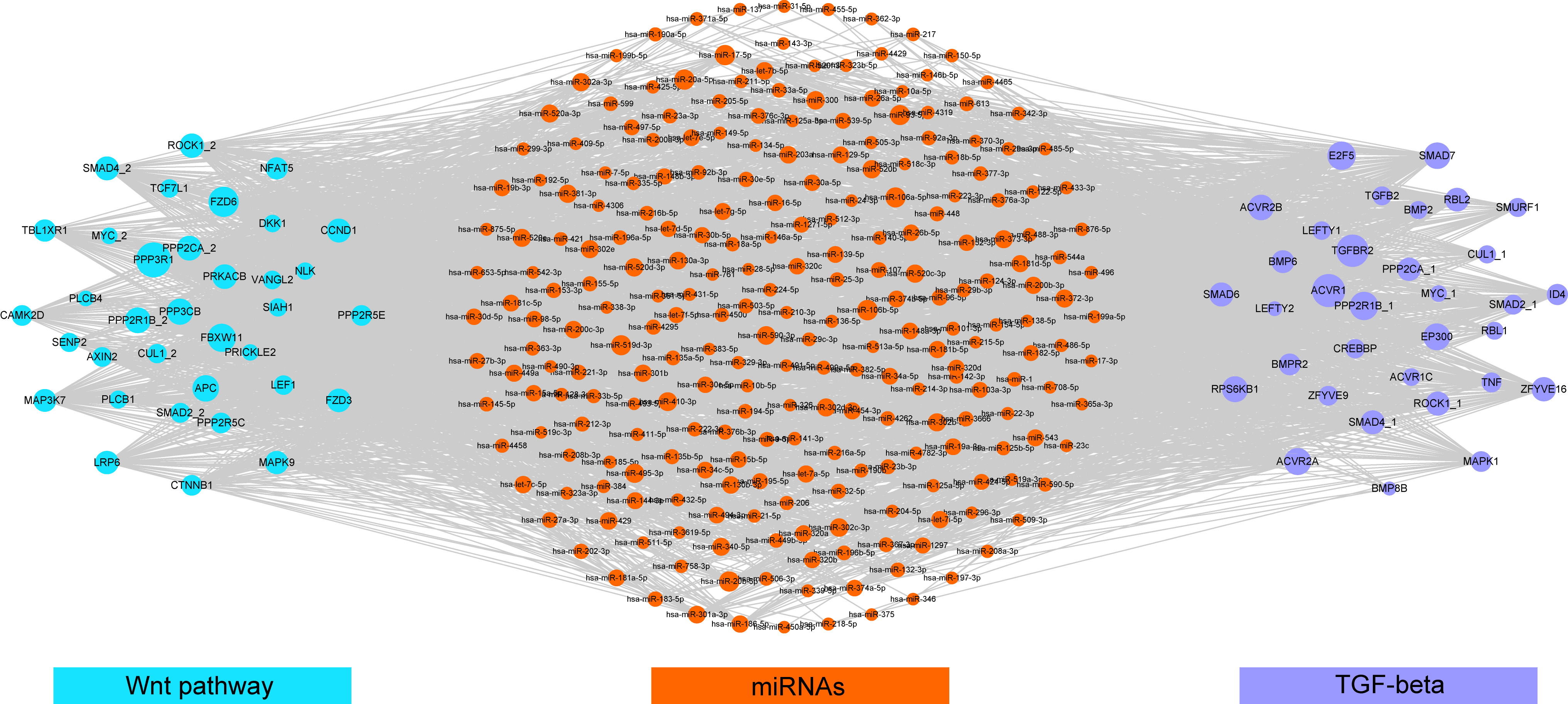
**

**Supplementary Figure S12: The comprehensive ceRNA crosstalks between Wnt pathway and TGF-beta signaling pathway.**

**Supplementary Table legends**

**Supplementary Table S1: The summary of data in our study**. In the present study, we downloaded 21 gene expression profiles of 8 CVDs. We listed the disease name, GSE number, platform, samples, ceRNA pairs, number of edges and number of nodes in each network.

| **Disease** | **Disease name** | **GSE number** | **Platform** | **Samples** | **ceRNA pairs** | **Number of edges** | **Number of nodes** |
| --- | --- | --- | --- | --- | --- | --- | --- |
| **CAD** | Coronary artery disease | GSE40231 | GPL570 | 278 | 236986 | 45276 | 1171 |
|  |  | GSE12288 | GPL96 | 222 | 25491 | 10775 | 782 |
| **HCM** | Hypertrophic cardiomyopathy | GSE36961 | GPL15389 | 145 | 50302 | 11823 | 901 |
| **HF** | Heart failure | GSE1145 | GPL570 | 90 | 52279 | 7600 | 844 |
|  |  | GSE2656 | GPL2041 | 49 | 17940 | 2623 | 491 |
|  |  | GSE5406 | GPL96 | 210 | 57256 | 32657 | 1028 |
|  |  | GSE57338 | GPL11532 | 313 | 95542 | 22652 | 1065 |
| **DCM** | Dilated cardiomyopathy | GSE3586 | GPL3050 | 28 | 8761 | 623 | 267 |
|  |  | GSE17800 | GPL570 | 48 | 40547 | 5706 | 747 |
| **MI** | Myocardial infarction | GSE48060 | GPL570 | 52 | 101896 | 23762 | 1043 |
|  |  | GSE60993 | GPL6884 | 33 | 32806 | 6499 | 695 |
|  |  | GSE62646 | GPL6244 | 98 | 91534 | 23512 | 1029 |
|  |  | GSE66360 | GPL570 | 99 | 134936 | 39066 | 1090 |
|  |  | GSE28454 | GPL6883 | 76 | 27271 | 7893 | 739 |
|  |  | GSE29111 | GPL570 | 52 | 40189 | 7238 | 800 |
|  |  | GSE34198 | GPL6102 | 97 | 356446 | 56226 | 1208 |
|  |  | GSE59867 | GPL6244 | 436 | 136969 | 32379 | 1097 |
|  |  | GSE49925 | GPL10558 | 338 | 52116 | 17062 | 949 |
| **PAH** | Pulmonary hypertension | GSE33463 | GPL6947 | 140 | 236397 | 58384 | 1158 |
| **ICM** | Ischemic cardiomyopathy | GSE1869 | GPL96 | 37 | 57922 | 32725 | 979 |
| **CHD** | Congenital Heart Disease | GSE34457 | GPL6102 | 43 | 332679 | 56510 | 1204 |

**Supplementary Table S2: Common modules of our study**. In total, 483 modules were identified from our study.

| module 1 |
| --- |
| BIOCARTA_NFKB_PATHWAY |
| BIOCARTA_TGFB_PATHWAY |
| PID_ALK1_PATHWAY |
| REACTOME_MITOTIC_M_M_G1_PHASES |
| BIOCARTA_SKP2E2F_PATHWAY |
| KEGG_FOCAL_ADHESION |
| module 2 |
| BIOCARTA_NFKB_PATHWAY |
| BIOCARTA_TGFB_PATHWAY |
| PID_ALK1_PATHWAY |
| REACTOME_DNA_REPLICATION |
| BIOCARTA_SKP2E2F_PATHWAY |
| KEGG_FOCAL_ADHESION |
| module 3 |
| BIOCARTA_NFKB_PATHWAY |
| BIOCARTA_TGFB_PATHWAY |
| REACTOME_MITOTIC_M_M_G1_PHASES |
| BIOCARTA_SKP2E2F_PATHWAY |
| KEGG_FOCAL_ADHESION |
| KEGG_TGF_BETA_SIGNALING_PATHWAY |
| module 4 |
| BIOCARTA_NFKB_PATHWAY |
| BIOCARTA_TGFB_PATHWAY |
| REACTOME_DNA_REPLICATION |
| BIOCARTA_SKP2E2F_PATHWAY |
| KEGG_FOCAL_ADHESION |
| KEGG_TGF_BETA_SIGNALING_PATHWAY |
| module 5 |
| BIOCARTA_TGFB_PATHWAY |
| PID_ALK1_PATHWAY |
| PID_WNT_NONCANONICAL_PATHWAY |
| REACTOME_APOPTOSIS |
| REACTOME_DNA_REPLICATION |
| BIOCARTA_SKP2E2F_PATHWAY |
| KEGG_FOCAL_ADHESION |
| module 6 |
| IOCARTA_TGFB_PATHWAY |
| PID_ALK1_PATHWAY |
| PID_WNT_NONCANONICAL_PATHWAY |
| REACTOME_EXTRINSIC_PATHWAY_FOR_APOPTOSIS |
| REACTOME_MITOTIC_M_M_G1_PHASES |
| BIOCARTA_SKP2E2F_PATHWAY |
| KEGG_FOCAL_ADHESION |
| module 7 |
| IOCARTA_TGFB_PATHWAY |
| PID_ALK1_PATHWAY |
| PID_WNT_NONCANONICAL_PATHWAY |
| REACTOME_EXTRINSIC_PATHWAY_FOR_APOPTOSIS |
| REACTOME_DNA_REPLICATION |
| BIOCARTA_SKP2E2F_PATHWAY |
| KEGG_FOCAL_ADHESION |
| module 8 |
| IOCARTA_TGFB_PATHWAY |
| PID_ALK1_PATHWAY |
| PID_WNT_NONCANONICAL_PATHWAY |
| REACTOME_MITOTIC_M_M_G1_PHASES |
| BIOCARTA_SKP2E2F_PATHWAY |
| KEGG_APOPTOSIS |
| KEGG_FOCAL_ADHESION |
| module 9 |
| IOCARTA_TGFB_PATHWAY |
| PID_ALK1_PATHWAY |
| PID_WNT_NONCANONICAL_PATHWAY |
| REACTOME_DNA_REPLICATION |
| BIOCARTA_SKP2E2F_PATHWAY |
| KEGG_APOPTOSIS |
| KEGG_FOCAL_ADHESION |
| module 10 |
| IOCARTA_TGFB_PATHWAY |
| PID_WNT_NONCANONICAL_PATHWAY |
| REACTOME_APOPTOSIS |
| REACTOME_DNA_REPLICATION |
| BIOCARTA_SKP2E2F_PATHWAY |
| KEGG_FOCAL_ADHESION |
| KEGG_TGF_BETA_SIGNALING_PATHWAY |
| module 11 |
| BIOCARTA_TGFB_PATHWAY |
| PID_WNT_NONCANONICAL_PATHWAY |
| REACTOME_EXTRINSIC_PATHWAY_FOR_APOPTOSIS |
| REACTOME_MITOTIC_M_M_G1_PHASES |
| BIOCARTA_SKP2E2F_PATHWAY |
| KEGG_FOCAL_ADHESION |
| KEGG_TGF_BETA_SIGNALING_PATHWAY |
| module 12 |
| BIOCARTA_TGFB_PATHWAY |
| PID_WNT_NONCANONICAL_PATHWAY |
| REACTOME_EXTRINSIC_PATHWAY_FOR_APOPTOSIS |
| REACTOME_DNA_REPLICATION |
| BIOCARTA_SKP2E2F_PATHWAY |
| KEGG_FOCAL_ADHESION |
| KEGG_TGF_BETA_SIGNALING_PATHWAY |
| module 13 |
| BIOCARTA_TGFB_PATHWAY |
| PID_WNT_NONCANONICAL_PATHWAY |
| REACTOME_MITOTIC_M_M_G1_PHASES |
| BIOCARTA_SKP2E2F_PATHWAY |
| KEGG_APOPTOSIS |
| KEGG_FOCAL_ADHESION |
| KEGG_TGF_BETA_SIGNALING_PATHWAY |
| module 14 |
| BIOCARTA_TGFB_PATHWAY |
| PID_WNT_NONCANONICAL_PATHWAY |
| REACTOME_DNA_REPLICATION |
| BIOCARTA_SKP2E2F_PATHWAY |
| KEGG_APOPTOSIS |
| KEGG_FOCAL_ADHESION |
| KEGG_TGF_BETA_SIGNALING_PATHWAY |
| module 15 |
| BIOCARTA_NFKB_PATHWAY |
| BIOCARTA_TGFB_PATHWAY |
| PID_ALK1_PATHWAY |
| REACTOME_ANTIGEN_PROCESSING_UBIQUITINATION_PROTEASOME_DEGRADATION |
| REACTOME_MITOTIC_G1_G1_S_PHASES |
| KEGG_FOCAL_ADHESION |
| module 16 |
| BIOCARTA_NFKB_PATHWAY |
| BIOCARTA_TGFB_PATHWAY |
| REACTOME_ANTIGEN_PROCESSING_UBIQUITINATION_PROTEASOME_DEGRADATION |
| REACTOME_MITOTIC_G1_G1_S_PHASES |
| REACTOME_SIGNALING_BY_BMP |
| KEGG_FOCAL_ADHESION |
| module 17 |
| BIOCARTA_NFKB_PATHWAY |
| BIOCARTA_TGFB_PATHWAY |
| REACTOME_ANTIGEN_PROCESSING_UBIQUITINATION_PROTEASOME_DEGRADATION |
| REACTOME_MITOTIC_G1_G1_S_PHASES |
| KEGG_FOCAL_ADHESION |
| KEGG_TGF_BETA_SIGNALING_PATHWAY |
| module 18 |
| BIOCARTA_TGFB_PATHWAY |
| PID_ALK1_PATHWAY |
| PID_WNT_NONCANONICAL_PATHWAY |
| REACTOME_ANTIGEN_PROCESSING_UBIQUITINATION_PROTEASOME_DEGRADATION |
| REACTOME_APOPTOSIS |
| REACTOME_MITOTIC_G1_G1_S_PHASES |
| KEGG_FOCAL_ADHESION |
| module 19 |
| BIOCARTA_TGFB_PATHWAY |
| PID_ALK1_PATHWAY |
| PID_WNT_NONCANONICAL_PATHWAY |
| REACTOME_ANTIGEN_PROCESSING_UBIQUITINATION_PROTEASOME_DEGRADATION |
| REACTOME_EXTRINSIC_PATHWAY_FOR_APOPTOSIS |
| REACTOME_MITOTIC_G1_G1_S_PHASES |
| KEGG_FOCAL_ADHESION |
| module 20 |
| BIOCARTA_TGFB_PATHWAY |
| PID_ALK1_PATHWAY |
| PID_WNT_NONCANONICAL_PATHWAY |
| REACTOME_ANTIGEN_PROCESSING_UBIQUITINATION_PROTEASOME_DEGRADATION |
| REACTOME_MITOTIC_G1_G1_S_PHASES |
| KEGG_APOPTOSIS |
| KEGG_FOCAL_ADHESION |
| module 21 |
| BIOCARTA_TGFB_PATHWAY |
| PID_ALK1_PATHWAY |
| REACTOME_ANTIGEN_PROCESSING_UBIQUITINATION_PROTEASOME_DEGRADATION |
| REACTOME_APOPTOSIS |
| REACTOME_MITOTIC_G1_G1_S_PHASES |
| KEGG_FOCAL_ADHESION |
| KEGG_WNT_SIGNALING_PATHWAY |
| module 22 |
| BIOCARTA_TGFB_PATHWAY |
| PID_ALK1_PATHWAY |
| REACTOME_ANTIGEN_PROCESSING_UBIQUITINATION_PROTEASOME_DEGRADATION |
| REACTOME_EXTRINSIC_PATHWAY_FOR_APOPTOSIS |
| REACTOME_MITOTIC_G1_G1_S_PHASES |
| KEGG_FOCAL_ADHESION |
| KEGG_WNT_SIGNALING_PATHWAY |
| module 23 |
| BIOCARTA_TGFB_PATHWAY |
| PID_ALK1_PATHWAY |
| REACTOME_ANTIGEN_PROCESSING_UBIQUITINATION_PROTEASOME_DEGRADATION |
| REACTOME_MITOTIC_G1_G1_S_PHASES |
| KEGG_APOPTOSIS |
| KEGG_FOCAL_ADHESION |
| KEGG_WNT_SIGNALING_PATHWAY |
| module 24 |
| BIOCARTA_TGFB_PATHWAY |
| PID_WNT_NONCANONICAL_PATHWAY |
| REACTOME_ANTIGEN_PROCESSING_UBIQUITINATION_PROTEASOME_DEGRADATION |
| REACTOME_APOPTOSIS |
| REACTOME_MITOTIC_G1_G1_S_PHASES |
| REACTOME_SIGNALING_BY_BMP |
| KEGG_FOCAL_ADHESION |
| module 25 |
| BIOCARTA_TGFB_PATHWAY |
| PID_WNT_NONCANONICAL_PATHWAY |
| REACTOME_ANTIGEN_PROCESSING_UBIQUITINATION_PROTEASOME_DEGRADATION |
| REACTOME_APOPTOSIS |
| REACTOME_MITOTIC_G1_G1_S_PHASES |
| PID_ALK2_PATHWAY |
| KEGG_FOCAL_ADHESION |
| module 26 |
| BIOCARTA_TGFB_PATHWAY |
| PID_WNT_NONCANONICAL_PATHWAY |
| REACTOME_ANTIGEN_PROCESSING_UBIQUITINATION_PROTEASOME_DEGRADATION |
| REACTOME_APOPTOSIS |
| REACTOME_MITOTIC_G1_G1_S_PHASES |
| KEGG_FOCAL_ADHESION |
| KEGG_TGF_BETA_SIGNALING_PATHWAY |
| module 27 |
| BIOCARTA_TGFB_PATHWAY |
| PID_WNT_NONCANONICAL_PATHWAY |
| REACTOME_ANTIGEN_PROCESSING_UBIQUITINATION_PROTEASOME_DEGRADATION |
| REACTOME_EXTRINSIC_PATHWAY_FOR_APOPTOSIS |
| REACTOME_MITOTIC_G1_G1_S_PHASES |
| REACTOME_SIGNALING_BY_BMP |
| KEGG_FOCAL_ADHESION |
| module 28 |
| BIOCARTA_TGFB_PATHWAY |
| PID_WNT_NONCANONICAL_PATHWAY |
| REACTOME_ANTIGEN_PROCESSING_UBIQUITINATION_PROTEASOME_DEGRADATION |
| REACTOME_EXTRINSIC_PATHWAY_FOR_APOPTOSIS |
| REACTOME_MITOTIC_G1_G1_S_PHASES |
| KEGG_FOCAL_ADHESION |
| KEGG_TGF_BETA_SIGNALING_PATHWAY |
| module 29 |
| BIOCARTA_TGFB_PATHWAY |
| PID_WNT_NONCANONICAL_PATHWAY |
| REACTOME_ANTIGEN_PROCESSING_UBIQUITINATION_PROTEASOME_DEGRADATION |
| REACTOME_MITOTIC_G1_G1_S_PHASES |
| REACTOME_SIGNALING_BY_BMP |
| KEGG_APOPTOSIS |
| KEGG_FOCAL_ADHESION |
| module 30 |
| BIOCARTA_TGFB_PATHWAY |
| PID_WNT_NONCANONICAL_PATHWAY |
| REACTOME_ANTIGEN_PROCESSING_UBIQUITINATION_PROTEASOME_DEGRADATION |
| REACTOME_MITOTIC_G1_G1_S_PHASES |
| PID_ALK2_PATHWAY |
| KEGG_APOPTOSIS |
| KEGG_FOCAL_ADHESION |
| module 31 |
| BIOCARTA_TGFB_PATHWAY |
| PID_WNT_NONCANONICAL_PATHWAY |
| REACTOME_ANTIGEN_PROCESSING_UBIQUITINATION_PROTEASOME_DEGRADATION |
| REACTOME_MITOTIC_G1_G1_S_PHASES |
| KEGG_APOPTOSIS |
| KEGG_FOCAL_ADHESION |
| KEGG_TGF_BETA_SIGNALING_PATHWAY |
| module 32 |
| BIOCARTA_TGFB_PATHWAY |
| REACTOME_ANTIGEN_PROCESSING_UBIQUITINATION_PROTEASOME_DEGRADATION |
| REACTOME_APOPTOSIS |
| REACTOME_MITOTIC_G1_G1_S_PHASES |
| REACTOME_SIGNALING_BY_BMP |
| KEGG_FOCAL_ADHESION |
| KEGG_WNT_SIGNALING_PATHWAY |
| module 33 |
| BIOCARTA_TGFB_PATHWAY |
| REACTOME_ANTIGEN_PROCESSING_UBIQUITINATION_PROTEASOME_DEGRADATION |
| REACTOME_APOPTOSIS |
| REACTOME_MITOTIC_G1_G1_S_PHASES |
| PID_ALK2_PATHWAY |
| KEGG_FOCAL_ADHESION |
| KEGG_WNT_SIGNALING_PATHWAY |
| module 34 |
| BIOCARTA_TGFB_PATHWAY |
| REACTOME_ANTIGEN_PROCESSING_UBIQUITINATION_PROTEASOME_DEGRADATION |
| REACTOME_APOPTOSIS |
| REACTOME_MITOTIC_G1_G1_S_PHASES |
| KEGG_FOCAL_ADHESION |
| KEGG_TGF_BETA_SIGNALING_PATHWAY |
| KEGG_WNT_SIGNALING_PATHWAY |
| module 35 |
| BIOCARTA_TGFB_PATHWAY |
| REACTOME_ANTIGEN_PROCESSING_UBIQUITINATION_PROTEASOME_DEGRADATION |
| REACTOME_EXTRINSIC_PATHWAY_FOR_APOPTOSIS |
| REACTOME_MITOTIC_G1_G1_S_PHASES |
| REACTOME_SIGNALING_BY_BMP |
| KEGG_FOCAL_ADHESION |
| KEGG_WNT_SIGNALING_PATHWAY |
| module 36 |
| BIOCARTA_TGFB_PATHWAY |
| REACTOME_ANTIGEN_PROCESSING_UBIQUITINATION_PROTEASOME_DEGRADATION |
| REACTOME_EXTRINSIC_PATHWAY_FOR_APOPTOSIS |
| REACTOME_MITOTIC_G1_G1_S_PHASES |
| KEGG_FOCAL_ADHESION |
| KEGG_TGF_BETA_SIGNALING_PATHWAY |
| KEGG_WNT_SIGNALING_PATHWAY |
| module 37 |
| BIOCARTA_TGFB_PATHWAY |
| REACTOME_ANTIGEN_PROCESSING_UBIQUITINATION_PROTEASOME_DEGRADATION |
| REACTOME_MITOTIC_G1_G1_S_PHASES |
| REACTOME_SIGNALING_BY_BMP |
| KEGG_APOPTOSIS |
| KEGG_FOCAL_ADHESION |
| KEGG_WNT_SIGNALING_PATHWAY |
| module 38 |
| BIOCARTA_TGFB_PATHWAY |
| REACTOME_ANTIGEN_PROCESSING_UBIQUITINATION_PROTEASOME_DEGRADATION |
| REACTOME_MITOTIC_G1_G1_S_PHASES |
| PID_ALK2_PATHWAY |
| KEGG_APOPTOSIS |
| KEGG_FOCAL_ADHESION |
| KEGG_WNT_SIGNALING_PATHWAY |
| module 39 |
| BIOCARTA_TGFB_PATHWAY |
| REACTOME_ANTIGEN_PROCESSING_UBIQUITINATION_PROTEASOME_DEGRADATION |
| REACTOME_MITOTIC_G1_G1_S_PHASES |
| KEGG_APOPTOSIS |
| KEGG_FOCAL_ADHESION |
| KEGG_TGF_BETA_SIGNALING_PATHWAY |
| KEGG_WNT_SIGNALING_PATHWAY |
| module 40 |
| BIOCARTA_CELLCYCLE_PATHWAY |
| BIOCARTA_NFKB_PATHWAY |
| BIOCARTA_TGFB_PATHWAY |
| PID_ALK1_PATHWAY |
| REACTOME_ANTIGEN_PROCESSING_UBIQUITINATION_PROTEASOME_DEGRADATION |
| REACTOME_G1_PHASE |
| REACTOME_MITOTIC_M_M_G1_PHASES |
| KEGG_FOCAL_ADHESION |
| module 41 |
| BIOCARTA_CELLCYCLE_PATHWAY |
| BIOCARTA_NFKB_PATHWAY |
| BIOCARTA_TGFB_PATHWAY |
| PID_ALK1_PATHWAY |
| REACTOME_CLASS_I_MHC_MEDIATED_ANTIGEN_PROCESSING_PRESENTATION |
| REACTOME_G1_PHASE |
| REACTOME_MITOTIC_M_M_G1_PHASES |
| KEGG_FOCAL_ADHESION |
| module 42 |
| BIOCARTA_CELLCYCLE_PATHWAY |
| BIOCARTA_NFKB_PATHWAY |
| BIOCARTA_TGFB_PATHWAY |
| REACTOME_ANTIGEN_PROCESSING_UBIQUITINATION_PROTEASOME_DEGRADATION |
| REACTOME_G1_PHASE |
| REACTOME_MITOTIC_M_M_G1_PHASES |
| KEGG_FOCAL_ADHESION |
| KEGG_TGF_BETA_SIGNALING_PATHWAY |
| module 43 |
| BIOCARTA_CELLCYCLE_PATHWAY |
| BIOCARTA_NFKB_PATHWAY |
| BIOCARTA_TGFB_PATHWAY |
| REACTOME_CLASS_I_MHC_MEDIATED_ANTIGEN_PROCESSING_PRESENTATION |
| REACTOME_G1_PHASE |
| REACTOME_MITOTIC_M_M_G1_PHASES |
| KEGG_FOCAL_ADHESION |
| KEGG_TGF_BETA_SIGNALING_PATHWAY |
| module 44 |
| BIOCARTA_CELLCYCLE_PATHWAY |
| BIOCARTA_TGFB_PATHWAY |
| PID_ALK1_PATHWAY |
| PID_WNT_NONCANONICAL_PATHWAY |
| REACTOME_ADAPTIVE_IMMUNE_SYSTEM |
| REACTOME_APOPTOSIS |
| REACTOME_G1_PHASE |
| KEGG_FOCAL_ADHESION |
| module 45 |
| BIOCARTA_CELLCYCLE_PATHWAY |
| BIOCARTA_TGFB_PATHWAY |
| PID_ALK1_PATHWAY |
| PID_WNT_NONCANONICAL_PATHWAY |
| REACTOME_ADAPTIVE_IMMUNE_SYSTEM |
| REACTOME_EXTRINSIC_PATHWAY_FOR_APOPTOSIS |
| REACTOME_G1_PHASE |
| KEGG_FOCAL_ADHESION |
| module 46 |
| BIOCARTA_CELLCYCLE_PATHWAY |
| BIOCARTA_TGFB_PATHWAY |
| PID_ALK1_PATHWAY |
| PID_WNT_NONCANONICAL_PATHWAY |
| REACTOME_ADAPTIVE_IMMUNE_SYSTEM |
| REACTOME_G1_PHASE |
| KEGG_APOPTOSIS |
| KEGG_FOCAL_ADHESION |
| module 47 |
| BIOCARTA_CELLCYCLE_PATHWAY |
| BIOCARTA_TGFB_PATHWAY |
| PID_ALK1_PATHWAY |
| PID_WNT_NONCANONICAL_PATHWAY |
| REACTOME_ANTIGEN_PROCESSING_UBIQUITINATION_PROTEASOME_DEGRADATION |
| REACTOME_APOPTOSIS |
| REACTOME_G1_PHASE |
| KEGG_FOCAL_ADHESION |
| module 48 |
| BIOCARTA_CELLCYCLE_PATHWAY |
| BIOCARTA_TGFB_PATHWAY |
| PID_ALK1_PATHWAY |
| PID_WNT_NONCANONICAL_PATHWAY |
| REACTOME_ANTIGEN_PROCESSING_UBIQUITINATION_PROTEASOME_DEGRADATION |
| REACTOME_EXTRINSIC_PATHWAY_FOR_APOPTOSIS |
| REACTOME_G1_PHASE |
| REACTOME_MITOTIC_M_M_G1_PHASES |
| KEGG_FOCAL_ADHESION |
| module 49 |
| BIOCARTA_CELLCYCLE_PATHWAY |
| BIOCARTA_TGFB_PATHWAY |
| PID_ALK1_PATHWAY |
| PID_WNT_NONCANONICAL_PATHWAY |
| REACTOME_ANTIGEN_PROCESSING_UBIQUITINATION_PROTEASOME_DEGRADATION |
| REACTOME_G1_PHASE |
| REACTOME_MITOTIC_M_M_G1_PHASES |
| KEGG_APOPTOSIS |
| KEGG_FOCAL_ADHESION |
| module 50 |
| BIOCARTA_CELLCYCLE_PATHWAY |
| BIOCARTA_TGFB_PATHWAY |
| PID_ALK1_PATHWAY |
| PID_WNT_NONCANONICAL_PATHWAY |
| REACTOME_APOPTOSIS |
| REACTOME_CLASS_I_MHC_MEDIATED_ANTIGEN_PROCESSING_PRESENTATION |
| REACTOME_G1_PHASE |
| KEGG_FOCAL_ADHESION |
| module 51 |
| BIOCARTA_CELLCYCLE_PATHWAY |
| BIOCARTA_TGFB_PATHWAY |
| PID_ALK1_PATHWAY |
| PID_WNT_NONCANONICAL_PATHWAY |
| REACTOME_APOPTOSIS |
| REACTOME_G1_PHASE |
| REACTOME_IMMUNE_SYSTEM |
| KEGG_FOCAL_ADHESION |
| module 52 |
| BIOCARTA_CELLCYCLE_PATHWAY |
| BIOCARTA_TGFB_PATHWAY |
| PID_ALK1_PATHWAY |
| PID_WNT_NONCANONICAL_PATHWAY |
| REACTOME_CLASS_I_MHC_MEDIATED_ANTIGEN_PROCESSING_PRESENTATION |
| REACTOME_EXTRINSIC_PATHWAY_FOR_APOPTOSIS |
| REACTOME_G1_PHASE |
| REACTOME_MITOTIC_M_M_G1_PHASES |
| KEGG_FOCAL_ADHESION |
| module 53 |
| BIOCARTA_CELLCYCLE_PATHWAY |
| BIOCARTA_TGFB_PATHWAY |
| PID_ALK1_PATHWAY |
| PID_WNT_NONCANONICAL_PATHWAY |
| REACTOME_CLASS_I_MHC_MEDIATED_ANTIGEN_PROCESSING_PRESENTATION |
| REACTOME_G1_PHASE |
| REACTOME_MITOTIC_M_M_G1_PHASES |
| KEGG_APOPTOSIS |
| KEGG_FOCAL_ADHESION |
| module 54 |
| BIOCARTA_CELLCYCLE_PATHWAY |
| BIOCARTA_TGFB_PATHWAY |
| PID_ALK1_PATHWAY |
| PID_WNT_NONCANONICAL_PATHWAY |
| REACTOME_EXTRINSIC_PATHWAY_FOR_APOPTOSIS |
| REACTOME_G1_PHASE |
| REACTOME_IMMUNE_SYSTEM |
| KEGG_FOCAL_ADHESION |
| module 55 |
| BIOCARTA_CELLCYCLE_PATHWAY |
| BIOCARTA_TGFB_PATHWAY |
| PID_ALK1_PATHWAY |
| PID_WNT_NONCANONICAL_PATHWAY |
| REACTOME_G1_PHASE |
| REACTOME_IMMUNE_SYSTEM |
| KEGG_APOPTOSIS |
| KEGG_FOCAL_ADHESION |
| module 56 |
| BIOCARTA_CELLCYCLE_PATHWAY |
| BIOCARTA_TGFB_PATHWAY |
| PID_ALK1_PATHWAY |
| REACTOME_ADAPTIVE_IMMUNE_SYSTEM |
| REACTOME_APOPTOSIS |
| REACTOME_G1_PHASE |
| KEGG_FOCAL_ADHESION |
| KEGG_WNT_SIGNALING_PATHWAY |
| module 57 |
| BIOCARTA_CELLCYCLE_PATHWAY |
| BIOCARTA_TGFB_PATHWAY |
| PID_ALK1_PATHWAY |
| REACTOME_ADAPTIVE_IMMUNE_SYSTEM |
| REACTOME_EXTRINSIC_PATHWAY_FOR_APOPTOSIS |
| REACTOME_G1_PHASE |
| KEGG_FOCAL_ADHESION |
| KEGG_WNT_SIGNALING_PATHWAY |
| module 58 |
| BIOCARTA_CELLCYCLE_PATHWAY |
| BIOCARTA_TGFB_PATHWAY |
| PID_ALK1_PATHWAY |
| REACTOME_ADAPTIVE_IMMUNE_SYSTEM |
| REACTOME_G1_PHASE |
| KEGG_APOPTOSIS |
| KEGG_FOCAL_ADHESION |
| KEGG_WNT_SIGNALING_PATHWAY |
| module 59 |
| BIOCARTA_CELLCYCLE_PATHWAY |
| BIOCARTA_TGFB_PATHWAY |
| PID_ALK1_PATHWAY |
| REACTOME_ANTIGEN_PROCESSING_UBIQUITINATION_PROTEASOME_DEGRADATION |
| REACTOME_APOPTOSIS |
| REACTOME_G1_PHASE |
| KEGG_FOCAL_ADHESION |
| KEGG_WNT_SIGNALING_PATHWAY |
| module 60 |
| BIOCARTA_CELLCYCLE_PATHWAY |
| BIOCARTA_TGFB_PATHWAY |
| PID_ALK1_PATHWAY |
| REACTOME_ANTIGEN_PROCESSING_UBIQUITINATION_PROTEASOME_DEGRADATION |
| REACTOME_EXTRINSIC_PATHWAY_FOR_APOPTOSIS |
| REACTOME_G1_PHASE |
| REACTOME_MITOTIC_M_M_G1_PHASES |
| KEGG_FOCAL_ADHESION |
| KEGG_WNT_SIGNALING_PATHWAY |
| module 61 |
| BIOCARTA_CELLCYCLE_PATHWAY |
| BIOCARTA_TGFB_PATHWAY |
| PID_ALK1_PATHWAY |
| REACTOME_ANTIGEN_PROCESSING_UBIQUITINATION_PROTEASOME_DEGRADATION |
| REACTOME_G1_PHASE |
| REACTOME_MITOTIC_M_M_G1_PHASES |
| KEGG_APOPTOSIS |
| KEGG_FOCAL_ADHESION |
| KEGG_WNT_SIGNALING_PATHWAY |
| module 62 |
| BIOCARTA_CELLCYCLE_PATHWAY |
| BIOCARTA_TGFB_PATHWAY |
| PID_ALK1_PATHWAY |
| REACTOME_APOPTOSIS |
| REACTOME_CLASS_I_MHC_MEDIATED_ANTIGEN_PROCESSING_PRESENTATION |
| REACTOME_G1_PHASE |
| KEGG_FOCAL_ADHESION |
| KEGG_WNT_SIGNALING_PATHWAY |
| module 63 |
| BIOCARTA_CELLCYCLE_PATHWAY |
| BIOCARTA_TGFB_PATHWAY |
| PID_ALK1_PATHWAY |
| REACTOME_APOPTOSIS |
| REACTOME_G1_PHASE |
| REACTOME_IMMUNE_SYSTEM |
| KEGG_FOCAL_ADHESION |
| KEGG_WNT_SIGNALING_PATHWAY |
| module 64 |
| BIOCARTA_CELLCYCLE_PATHWAY |
| BIOCARTA_TGFB_PATHWAY |
| PID_ALK1_PATHWAY |
| REACTOME_CLASS_I_MHC_MEDIATED_ANTIGEN_PROCESSING_PRESENTATION |
| REACTOME_EXTRINSIC_PATHWAY_FOR_APOPTOSIS |
| REACTOME_G1_PHASE |
| REACTOME_MITOTIC_M_M_G1_PHASES |
| KEGG_FOCAL_ADHESION |
| KEGG_WNT_SIGNALING_PATHWAY |
| module 65 |
| BIOCARTA_CELLCYCLE_PATHWAY |
| BIOCARTA_TGFB_PATHWAY |
| PID_ALK1_PATHWAY |
| REACTOME_CLASS_I_MHC_MEDIATED_ANTIGEN_PROCESSING_PRESENTATION |
| REACTOME_G1_PHASE |
| REACTOME_MITOTIC_M_M_G1_PHASES |
| KEGG_APOPTOSIS |
| KEGG_FOCAL_ADHESION |
| KEGG_WNT_SIGNALING_PATHWAY |
| module 66 |
| BIOCARTA_CELLCYCLE_PATHWAY |
| BIOCARTA_TGFB_PATHWAY |
| PID_ALK1_PATHWAY |
| REACTOME_EXTRINSIC_PATHWAY_FOR_APOPTOSIS |
| REACTOME_G1_PHASE |
| REACTOME_IMMUNE_SYSTEM |
| KEGG_FOCAL_ADHESION |
| KEGG_WNT_SIGNALING_PATHWAY |
| module 67 |
| BIOCARTA_CELLCYCLE_PATHWAY |
| BIOCARTA_TGFB_PATHWAY |
| PID_ALK1_PATHWAY |
| REACTOME_G1_PHASE |
| REACTOME_IMMUNE_SYSTEM |
| KEGG_APOPTOSIS |
| KEGG_FOCAL_ADHESION |
| KEGG_WNT_SIGNALING_PATHWAY |
| module 68 |
| BIOCARTA_CELLCYCLE_PATHWAY |
| BIOCARTA_TGFB_PATHWAY |
| PID_WNT_NONCANONICAL_PATHWAY |
| REACTOME_ADAPTIVE_IMMUNE_SYSTEM |
| REACTOME_APOPTOSIS |
| REACTOME_G1_PHASE |
| KEGG_FOCAL_ADHESION |
| KEGG_TGF_BETA_SIGNALING_PATHWAY |
| module 69 |
| BIOCARTA_CELLCYCLE_PATHWAY |
| BIOCARTA_TGFB_PATHWAY |
| PID_WNT_NONCANONICAL_PATHWAY |
| REACTOME_ADAPTIVE_IMMUNE_SYSTEM |
| REACTOME_EXTRINSIC_PATHWAY_FOR_APOPTOSIS |
| REACTOME_G1_PHASE |
| KEGG_FOCAL_ADHESION |
| KEGG_TGF_BETA_SIGNALING_PATHWAY |
| module 70 |
| BIOCARTA_CELLCYCLE_PATHWAY |
| BIOCARTA_TGFB_PATHWAY |
| PID_WNT_NONCANONICAL_PATHWAY |
| REACTOME_ADAPTIVE_IMMUNE_SYSTEM |
| REACTOME_G1_PHASE |
| KEGG_APOPTOSIS |
| KEGG_FOCAL_ADHESION |
| KEGG_TGF_BETA_SIGNALING_PATHWAY |
| module 71 |
| BIOCARTA_CELLCYCLE_PATHWAY |
| BIOCARTA_TGFB_PATHWAY |
| PID_WNT_NONCANONICAL_PATHWAY |
| REACTOME_ANTIGEN_PROCESSING_UBIQUITINATION_PROTEASOME_DEGRADATION |
| REACTOME_APOPTOSIS |
| REACTOME_G1_PHASE |
| KEGG_FOCAL_ADHESION |
| KEGG_TGF_BETA_SIGNALING_PATHWAY |
| module 72 |
| BIOCARTA_CELLCYCLE_PATHWAY |
| BIOCARTA_TGFB_PATHWAY |
| PID_WNT_NONCANONICAL_PATHWAY |
| REACTOME_ANTIGEN_PROCESSING_UBIQUITINATION_PROTEASOME_DEGRADATION |
| REACTOME_EXTRINSIC_PATHWAY_FOR_APOPTOSIS |
| REACTOME_G1_PHASE |
| REACTOME_MITOTIC_M_M_G1_PHASES |
| KEGG_FOCAL_ADHESION |
| KEGG_TGF_BETA_SIGNALING_PATHWAY |
| module 73 |
| BIOCARTA_CELLCYCLE_PATHWAY |
| BIOCARTA_TGFB_PATHWAY |
| PID_WNT_NONCANONICAL_PATHWAY |
| REACTOME_ANTIGEN_PROCESSING_UBIQUITINATION_PROTEASOME_DEGRADATION |
| REACTOME_G1_PHASE |
| REACTOME_MITOTIC_M_M_G1_PHASES |
| KEGG_APOPTOSIS |
| KEGG_FOCAL_ADHESION |
| KEGG_TGF_BETA_SIGNALING_PATHWAY |
| module 74 |
| BIOCARTA_CELLCYCLE_PATHWAY |
| BIOCARTA_TGFB_PATHWAY |
| PID_WNT_NONCANONICAL_PATHWAY |
| REACTOME_APOPTOSIS |
| REACTOME_CLASS_I_MHC_MEDIATED_ANTIGEN_PROCESSING_PRESENTATION |
| REACTOME_G1_PHASE |
| KEGG_FOCAL_ADHESION |
| KEGG_TGF_BETA_SIGNALING_PATHWAY |
| module 75 |
| BIOCARTA_CELLCYCLE_PATHWAY |
| BIOCARTA_TGFB_PATHWAY |
| PID_WNT_NONCANONICAL_PATHWAY |
| REACTOME_APOPTOSIS |
| REACTOME_G1_PHASE |
| REACTOME_IMMUNE_SYSTEM |
| KEGG_FOCAL_ADHESION |
| KEGG_TGF_BETA_SIGNALING_PATHWAY |
| module 76 |
| BIOCARTA_CELLCYCLE_PATHWAY |
| BIOCARTA_TGFB_PATHWAY |
| PID_WNT_NONCANONICAL_PATHWAY |
| REACTOME_CLASS_I_MHC_MEDIATED_ANTIGEN_PROCESSING_PRESENTATION |
| REACTOME_EXTRINSIC_PATHWAY_FOR_APOPTOSIS |
| REACTOME_G1_PHASE |
| REACTOME_MITOTIC_M_M_G1_PHASES |
| KEGG_FOCAL_ADHESION |
| KEGG_TGF_BETA_SIGNALING_PATHWAY |
| module 77 |
| BIOCARTA_CELLCYCLE_PATHWAY |
| BIOCARTA_TGFB_PATHWAY |
| PID_WNT_NONCANONICAL_PATHWAY |
| REACTOME_CLASS_I_MHC_MEDIATED_ANTIGEN_PROCESSING_PRESENTATION |
| REACTOME_G1_PHASE |
| REACTOME_MITOTIC_M_M_G1_PHASES |
| KEGG_APOPTOSIS |
| KEGG_FOCAL_ADHESION |
| KEGG_TGF_BETA_SIGNALING_PATHWAY |
| module 78 |
| BIOCARTA_CELLCYCLE_PATHWAY |
| BIOCARTA_TGFB_PATHWAY |
| PID_WNT_NONCANONICAL_PATHWAY |
| REACTOME_EXTRINSIC_PATHWAY_FOR_APOPTOSIS |
| REACTOME_G1_PHASE |
| REACTOME_IMMUNE_SYSTEM |
| KEGG_FOCAL_ADHESION |
| KEGG_TGF_BETA_SIGNALING_PATHWAY |
| module 79 |
| BIOCARTA_CELLCYCLE_PATHWAY |
| BIOCARTA_TGFB_PATHWAY |
| PID_WNT_NONCANONICAL_PATHWAY |
| REACTOME_G1_PHASE |
| REACTOME_IMMUNE_SYSTEM |
| KEGG_APOPTOSIS |
| KEGG_FOCAL_ADHESION |
| KEGG_TGF_BETA_SIGNALING_PATHWAY |
| module 80 |
| BIOCARTA_CELLCYCLE_PATHWAY |
| BIOCARTA_TGFB_PATHWAY |
| REACTOME_ADAPTIVE_IMMUNE_SYSTEM |
| REACTOME_APOPTOSIS |
| REACTOME_G1_PHASE |
| KEGG_FOCAL_ADHESION |
| KEGG_TGF_BETA_SIGNALING_PATHWAY |
| KEGG_WNT_SIGNALING_PATHWAY |
| module 81 |
| BIOCARTA_CELLCYCLE_PATHWAY |
| BIOCARTA_TGFB_PATHWAY |
| REACTOME_ADAPTIVE_IMMUNE_SYSTEM |
| REACTOME_EXTRINSIC_PATHWAY_FOR_APOPTOSIS |
| REACTOME_G1_PHASE |
| KEGG_FOCAL_ADHESION |
| KEGG_TGF_BETA_SIGNALING_PATHWAY |
| KEGG_WNT_SIGNALING_PATHWAY |
| module 82 |
| BIOCARTA_CELLCYCLE_PATHWAY |
| BIOCARTA_TGFB_PATHWAY |
| REACTOME_ADAPTIVE_IMMUNE_SYSTEM |
| REACTOME_G1_PHASE |
| KEGG_APOPTOSIS |
| KEGG_FOCAL_ADHESION |
| KEGG_TGF_BETA_SIGNALING_PATHWAY |
| KEGG_WNT_SIGNALING_PATHWAY |
| module 83 |
| BIOCARTA_CELLCYCLE_PATHWAY |
| BIOCARTA_TGFB_PATHWAY |
| REACTOME_ANTIGEN_PROCESSING_UBIQUITINATION_PROTEASOME_DEGRADATION |
| REACTOME_APOPTOSIS |
| REACTOME_G1_PHASE |
| KEGG_FOCAL_ADHESION |
| KEGG_TGF_BETA_SIGNALING_PATHWAY |
| KEGG_WNT_SIGNALING_PATHWAY |
| module 84 |
| BIOCARTA_CELLCYCLE_PATHWAY |
| BIOCARTA_TGFB_PATHWAY |
| REACTOME_ANTIGEN_PROCESSING_UBIQUITINATION_PROTEASOME_DEGRADATION |
| REACTOME_EXTRINSIC_PATHWAY_FOR_APOPTOSIS |
| REACTOME_G1_PHASE |
| REACTOME_MITOTIC_M_M_G1_PHASES |
| KEGG_FOCAL_ADHESION |
| KEGG_TGF_BETA_SIGNALING_PATHWAY |
| KEGG_WNT_SIGNALING_PATHWAY |
| module 85 |
| BIOCARTA_CELLCYCLE_PATHWAY |
| BIOCARTA_TGFB_PATHWAY |
| REACTOME_ANTIGEN_PROCESSING_UBIQUITINATION_PROTEASOME_DEGRADATION |
| REACTOME_G1_PHASE |
| REACTOME_MITOTIC_M_M_G1_PHASES |
| KEGG_APOPTOSIS |
| KEGG_FOCAL_ADHESION |
| KEGG_TGF_BETA_SIGNALING_PATHWAY |
| KEGG_WNT_SIGNALING_PATHWAY |
| module 86 |
| BIOCARTA_CELLCYCLE_PATHWAY |
| BIOCARTA_TGFB_PATHWAY |
| REACTOME_APOPTOSIS |
| REACTOME_CLASS_I_MHC_MEDIATED_ANTIGEN_PROCESSING_PRESENTATION |
| REACTOME_G1_PHASE |
| KEGG_FOCAL_ADHESION |
| KEGG_TGF_BETA_SIGNALING_PATHWAY |
| KEGG_WNT_SIGNALING_PATHWAY |
| module 87 |
| BIOCARTA_CELLCYCLE_PATHWAY |
| BIOCARTA_TGFB_PATHWAY |
| REACTOME_APOPTOSIS |
| REACTOME_G1_PHASE |
| REACTOME_IMMUNE_SYSTEM |
| KEGG_FOCAL_ADHESION |
| KEGG_TGF_BETA_SIGNALING_PATHWAY |
| KEGG_WNT_SIGNALING_PATHWAY |
| module 88 |
| BIOCARTA_CELLCYCLE_PATHWAY |
| BIOCARTA_TGFB_PATHWAY |
| REACTOME_CLASS_I_MHC_MEDIATED_ANTIGEN_PROCESSING_PRESENTATION |
| REACTOME_EXTRINSIC_PATHWAY_FOR_APOPTOSIS |
| REACTOME_G1_PHASE |
| REACTOME_MITOTIC_M_M_G1_PHASES |
| KEGG_FOCAL_ADHESION |
| KEGG_TGF_BETA_SIGNALING_PATHWAY |
| KEGG_WNT_SIGNALING_PATHWAY |
| module 89 |
| BIOCARTA_CELLCYCLE_PATHWAY |
| BIOCARTA_TGFB_PATHWAY |
| REACTOME_CLASS_I_MHC_MEDIATED_ANTIGEN_PROCESSING_PRESENTATION |
| REACTOME_G1_PHASE |
| REACTOME_MITOTIC_M_M_G1_PHASES |
| KEGG_APOPTOSIS |
| KEGG_FOCAL_ADHESION |
| KEGG_TGF_BETA_SIGNALING_PATHWAY |
| KEGG_WNT_SIGNALING_PATHWAY |
| module 90 |
| BIOCARTA_CELLCYCLE_PATHWAY |
| BIOCARTA_TGFB_PATHWAY |
| REACTOME_EXTRINSIC_PATHWAY_FOR_APOPTOSIS |
| REACTOME_G1_PHASE |
| REACTOME_IMMUNE_SYSTEM |
| KEGG_FOCAL_ADHESION |
| KEGG_TGF_BETA_SIGNALING_PATHWAY |
| KEGG_WNT_SIGNALING_PATHWAY |
| module 91 |
| BIOCARTA_CELLCYCLE_PATHWAY |
| BIOCARTA_TGFB_PATHWAY |
| REACTOME_G1_PHASE |
| REACTOME_IMMUNE_SYSTEM |
| KEGG_APOPTOSIS |
| KEGG_FOCAL_ADHESION |
| KEGG_TGF_BETA_SIGNALING_PATHWAY |
| KEGG_WNT_SIGNALING_PATHWAY |
| module 92 |
| BIOCARTA_NFKB_PATHWAY |
| BIOCARTA_TGFB_PATHWAY |
| PID_ALK1_PATHWAY |
| REACTOME_ANTIGEN_PROCESSING_UBIQUITINATION_PROTEASOME_DEGRADATION |
| REACTOME_G1_PHASE |
| REACTOME_DNA_REPLICATION |
| KEGG_FOCAL_ADHESION |
| module 93 |
| BIOCARTA_NFKB_PATHWAY |
| BIOCARTA_TGFB_PATHWAY |
| PID_ALK1_PATHWAY |
| REACTOME_ANTIGEN_PROCESSING_UBIQUITINATION_PROTEASOME_DEGRADATION |
| REACTOME_MITOTIC_M_M_G1_PHASES |
| REACTOME_G0_AND_EARLY_G1 |
| KEGG_FOCAL_ADHESION |
| module 94 |
| BIOCARTA_NFKB_PATHWAY |
| BIOCARTA_TGFB_PATHWAY |
| PID_ALK1_PATHWAY |
| REACTOME_ANTIGEN_PROCESSING_UBIQUITINATION_PROTEASOME_DEGRADATION |
| REACTOME_CELL_CYCLE_MITOTIC |
| KEGG_FOCAL_ADHESION |
| module 95 |
| BIOCARTA_NFKB_PATHWAY |
| BIOCARTA_TGFB_PATHWAY |
| PID_ALK1_PATHWAY |
| REACTOME_ANTIGEN_PROCESSING_UBIQUITINATION_PROTEASOME_DEGRADATION |
| REACTOME_DNA_REPLICATION |
| REACTOME_G0_AND_EARLY_G1 |
| KEGG_FOCAL_ADHESION |
| module 96 |
| IOCARTA_NFKB_PATHWAY |
| BIOCARTA_TGFB_PATHWAY |
| PID_ALK1_PATHWAY |
| REACTOME_ANTIGEN_PROCESSING_UBIQUITINATION_PROTEASOME_DEGRADATION |
| KEGG_FOCAL_ADHESION |
| KEGG_CELL_CYCLE |
| module 97 |
| IOCARTA_NFKB_PATHWAY |
| BIOCARTA_TGFB_PATHWAY |
| PID_ALK1_PATHWAY |
| REACTOME_CLASS_I_MHC_MEDIATED_ANTIGEN_PROCESSING_PRESENTATION |
| REACTOME_G1_PHASE |
| REACTOME_DNA_REPLICATION |
| KEGG_FOCAL_ADHESION |
| module 98 |
| IOCARTA_NFKB_PATHWAY |
| BIOCARTA_TGFB_PATHWAY |
| PID_ALK1_PATHWAY |
| REACTOME_CLASS_I_MHC_MEDIATED_ANTIGEN_PROCESSING_PRESENTATION |
| REACTOME_MITOTIC_M_M_G1_PHASES |
| REACTOME_G0_AND_EARLY_G1 |
| KEGG_FOCAL_ADHESION |
| module 99 |
| IOCARTA_NFKB_PATHWAY |
| BIOCARTA_TGFB_PATHWAY |
| PID_ALK1_PATHWAY |
| REACTOME_CLASS_I_MHC_MEDIATED_ANTIGEN_PROCESSING_PRESENTATION |
| REACTOME_CELL_CYCLE_MITOTIC |
| KEGG_FOCAL_ADHESION |
| module 100 |
| IOCARTA_NFKB_PATHWAY |
| BIOCARTA_TGFB_PATHWAY |
| PID_ALK1_PATHWAY |
| REACTOME_CLASS_I_MHC_MEDIATED_ANTIGEN_PROCESSING_PRESENTATION |
| REACTOME_DNA_REPLICATION |
| REACTOME_G0_AND_EARLY_G1 |
| KEGG_FOCAL_ADHESION |
| module 101 |
| BIOCARTA_NFKB_PATHWAY |
| BIOCARTA_TGFB_PATHWAY |
| PID_ALK1_PATHWAY |
| REACTOME_CLASS_I_MHC_MEDIATED_ANTIGEN_PROCESSING_PRESENTATION |
| KEGG_FOCAL_ADHESION |
| KEGG_CELL_CYCLE |
| module 102 |
| BIOCARTA_NFKB_PATHWAY |
| BIOCARTA_TGFB_PATHWAY |
| REACTOME_ANTIGEN_PROCESSING_UBIQUITINATION_PROTEASOME_DEGRADATION |
| REACTOME_G1_PHASE |
| REACTOME_MITOTIC_M_M_G1_PHASES |
| REACTOME_SIGNALING_BY_BMP |
| KEGG_FOCAL_ADHESION |
| module 103 |
| BIOCARTA_NFKB_PATHWAY |
| BIOCARTA_TGFB_PATHWAY |
| REACTOME_ANTIGEN_PROCESSING_UBIQUITINATION_PROTEASOME_DEGRADATION |
| REACTOME_G1_PHASE |
| REACTOME_DNA_REPLICATION |
| REACTOME_SIGNALING_BY_BMP |
| KEGG_FOCAL_ADHESION |
| module 104 |
| BIOCARTA_NFKB_PATHWAY |
| BIOCARTA_TGFB_PATHWAY |
| REACTOME_ANTIGEN_PROCESSING_UBIQUITINATION_PROTEASOME_DEGRADATION |
| REACTOME_G1_PHASE |
| REACTOME_DNA_REPLICATION |
| KEGG_FOCAL_ADHESION |
| KEGG_TGF_BETA_SIGNALING_PATHWAY |
| module 105 |
| BIOCARTA_NFKB_PATHWAY |
| BIOCARTA_TGFB_PATHWAY |
| REACTOME_ANTIGEN_PROCESSING_UBIQUITINATION_PROTEASOME_DEGRADATION |
| REACTOME_MITOTIC_M_M_G1_PHASES |
| REACTOME_G0_AND_EARLY_G1 |
| REACTOME_SIGNALING_BY_BMP |
| KEGG_FOCAL_ADHESION |
| module 106 |
| BIOCARTA_NFKB_PATHWAY |
| BIOCARTA_TGFB_PATHWAY |
| REACTOME_ANTIGEN_PROCESSING_UBIQUITINATION_PROTEASOME_DEGRADATION |
| REACTOME_MITOTIC_M_M_G1_PHASES |
| REACTOME_G0_AND_EARLY_G1 |
| KEGG_FOCAL_ADHESION |
| KEGG_TGF_BETA_SIGNALING_PATHWAY |
| module 107 |
| BIOCARTA_NFKB_PATHWAY |
| BIOCARTA_TGFB_PATHWAY |
| REACTOME_ANTIGEN_PROCESSING_UBIQUITINATION_PROTEASOME_DEGRADATION |
| REACTOME_CELL_CYCLE_MITOTIC |
| REACTOME_SIGNALING_BY_BMP |
| KEGG_FOCAL_ADHESION |
| module 108 |
| BIOCARTA_NFKB_PATHWAY |
| BIOCARTA_TGFB_PATHWAY |
| REACTOME_ANTIGEN_PROCESSING_UBIQUITINATION_PROTEASOME_DEGRADATION |
| REACTOME_CELL_CYCLE_MITOTIC |
| KEGG_FOCAL_ADHESION |
| KEGG_TGF_BETA_SIGNALING_PATHWAY |
| module 109 |
| BIOCARTA_NFKB_PATHWAY |
| BIOCARTA_TGFB_PATHWAY |
| REACTOME_ANTIGEN_PROCESSING_UBIQUITINATION_PROTEASOME_DEGRADATION |
| REACTOME_DNA_REPLICATION |
| REACTOME_G0_AND_EARLY_G1 |
| REACTOME_SIGNALING_BY_BMP |
| KEGG_FOCAL_ADHESION |
| module 110 |
| BIOCARTA_NFKB_PATHWAY |
| BIOCARTA_TGFB_PATHWAY |
| REACTOME_ANTIGEN_PROCESSING_UBIQUITINATION_PROTEASOME_DEGRADATION |
| REACTOME_DNA_REPLICATION |
| REACTOME_G0_AND_EARLY_G1 |
| KEGG_FOCAL_ADHESION |
| KEGG_TGF_BETA_SIGNALING_PATHWAY |
| module 111 |
| BIOCARTA_NFKB_PATHWAY |
| BIOCARTA_TGFB_PATHWAY |
| REACTOME_ANTIGEN_PROCESSING_UBIQUITINATION_PROTEASOME_DEGRADATION |
| REACTOME_SIGNALING_BY_BMP |
| KEGG_FOCAL_ADHESION |
| KEGG_CELL_CYCLE |
| module 112 |
| BIOCARTA_NFKB_PATHWAY |
| BIOCARTA_TGFB_PATHWAY |
| REACTOME_ANTIGEN_PROCESSING_UBIQUITINATION_PROTEASOME_DEGRADATION |
| KEGG_FOCAL_ADHESION |
| KEGG_TGF_BETA_SIGNALING_PATHWAY |
| KEGG_CELL_CYCLE |
| module 113 |
| BIOCARTA_NFKB_PATHWAY |
| BIOCARTA_TGFB_PATHWAY |
| REACTOME_CLASS_I_MHC_MEDIATED_ANTIGEN_PROCESSING_PRESENTATION |
| REACTOME_G1_PHASE |
| REACTOME_MITOTIC_M_M_G1_PHASES |
| REACTOME_SIGNALING_BY_BMP |
| KEGG_FOCAL_ADHESION |
| module 114 |
| BIOCARTA_NFKB_PATHWAY |
| BIOCARTA_TGFB_PATHWAY |
| REACTOME_CLASS_I_MHC_MEDIATED_ANTIGEN_PROCESSING_PRESENTATION |
| REACTOME_G1_PHASE |
| REACTOME_DNA_REPLICATION |
| REACTOME_SIGNALING_BY_BMP |
| KEGG_FOCAL_ADHESION |
| module 115 |
| BIOCARTA_NFKB_PATHWAY |
| BIOCARTA_TGFB_PATHWAY |
| REACTOME_CLASS_I_MHC_MEDIATED_ANTIGEN_PROCESSING_PRESENTATION |
| REACTOME_G1_PHASE |
| REACTOME_DNA_REPLICATION |
| KEGG_FOCAL_ADHESION |
| KEGG_TGF_BETA_SIGNALING_PATHWAY |
| module 116 |
| BIOCARTA_NFKB_PATHWAY |
| BIOCARTA_TGFB_PATHWAY |
| REACTOME_CLASS_I_MHC_MEDIATED_ANTIGEN_PROCESSING_PRESENTATION |
| REACTOME_MITOTIC_M_M_G1_PHASES |
| REACTOME_G0_AND_EARLY_G1 |
| REACTOME_SIGNALING_BY_BMP |
| KEGG_FOCAL_ADHESION |
| module 117 |
| BIOCARTA_NFKB_PATHWAY |
| BIOCARTA_TGFB_PATHWAY |
| REACTOME_CLASS_I_MHC_MEDIATED_ANTIGEN_PROCESSING_PRESENTATION |
| REACTOME_MITOTIC_M_M_G1_PHASES |
| REACTOME_G0_AND_EARLY_G1 |
| KEGG_FOCAL_ADHESION |
| KEGG_TGF_BETA_SIGNALING_PATHWAY |
| module 118 |
| BIOCARTA_NFKB_PATHWAY |
| BIOCARTA_TGFB_PATHWAY |
| REACTOME_CLASS_I_MHC_MEDIATED_ANTIGEN_PROCESSING_PRESENTATION |
| REACTOME_CELL_CYCLE_MITOTIC |
| REACTOME_SIGNALING_BY_BMP |
| KEGG_FOCAL_ADHESION |
| module 119 |
| BIOCARTA_NFKB_PATHWAY |
| BIOCARTA_TGFB_PATHWAY |
| REACTOME_CLASS_I_MHC_MEDIATED_ANTIGEN_PROCESSING_PRESENTATION |
| REACTOME_CELL_CYCLE_MITOTIC |
| KEGG_FOCAL_ADHESION |
| KEGG_TGF_BETA_SIGNALING_PATHWAY |
| module 120 |
| BIOCARTA_NFKB_PATHWAY |
| BIOCARTA_TGFB_PATHWAY |
| REACTOME_CLASS_I_MHC_MEDIATED_ANTIGEN_PROCESSING_PRESENTATION |
| REACTOME_DNA_REPLICATION |
| REACTOME_G0_AND_EARLY_G1 |
| REACTOME_SIGNALING_BY_BMP |
| KEGG_FOCAL_ADHESION |
| module 121 |
| BIOCARTA_NFKB_PATHWAY |
| BIOCARTA_TGFB_PATHWAY |
| REACTOME_CLASS_I_MHC_MEDIATED_ANTIGEN_PROCESSING_PRESENTATION |
| REACTOME_DNA_REPLICATION |
| REACTOME_G0_AND_EARLY_G1 |
| KEGG_FOCAL_ADHESION |
| KEGG_TGF_BETA_SIGNALING_PATHWAY |
| module 122 |
| BIOCARTA_NFKB_PATHWAY |
| BIOCARTA_TGFB_PATHWAY |
| REACTOME_CLASS_I_MHC_MEDIATED_ANTIGEN_PROCESSING_PRESENTATION |
| REACTOME_SIGNALING_BY_BMP |
| KEGG_FOCAL_ADHESION |
| KEGG_CELL_CYCLE |
| module 123 |
| BIOCARTA_NFKB_PATHWAY |
| BIOCARTA_TGFB_PATHWAY |
| REACTOME_CLASS_I_MHC_MEDIATED_ANTIGEN_PROCESSING_PRESENTATION |
| KEGG_FOCAL_ADHESION |
| KEGG_TGF_BETA_SIGNALING_PATHWAY |
| KEGG_CELL_CYCLE |
| module 124 |
| BIOCARTA_TGFB_PATHWAY |
| PID_ALK1_PATHWAY |
| PID_WNT_NONCANONICAL_PATHWAY |
| REACTOME_APOPTOSIS |
| REACTOME_IMMUNE_SYSTEM |
| REACTOME_CELL_CYCLE_MITOTIC |
| KEGG_FOCAL_ADHESION |
| module 125 |
| BIOCARTA_TGFB_PATHWAY |
| PID_ALK1_PATHWAY |
| PID_WNT_NONCANONICAL_PATHWAY |
| REACTOME_APOPTOSIS |
| REACTOME_IMMUNE_SYSTEM |
| REACTOME_G0_AND_EARLY_G1 |
| KEGG_FOCAL_ADHESION |
| module 126 |
| BIOCARTA_TGFB_PATHWAY |
| PID_ALK1_PATHWAY |
| PID_WNT_NONCANONICAL_PATHWAY |
| REACTOME_APOPTOSIS |
| REACTOME_IMMUNE_SYSTEM |
| KEGG_FOCAL_ADHESION |
| KEGG_CELL_CYCLE |
| module 127 |
| BIOCARTA_TGFB_PATHWAY |
| PID_ALK1_PATHWAY |
| PID_WNT_NONCANONICAL_PATHWAY |
| REACTOME_EXTRINSIC_PATHWAY_FOR_APOPTOSIS |
| REACTOME_IMMUNE_SYSTEM |
| REACTOME_CELL_CYCLE_MITOTIC |
| KEGG_FOCAL_ADHESION |
| module 128 |
| BIOCARTA_TGFB_PATHWAY |
| PID_ALK1_PATHWAY |
| PID_WNT_NONCANONICAL_PATHWAY |
| REACTOME_EXTRINSIC_PATHWAY_FOR_APOPTOSIS |
| REACTOME_IMMUNE_SYSTEM |
| REACTOME_G0_AND_EARLY_G1 |
| KEGG_FOCAL_ADHESION |
| module 129 |
| BIOCARTA_TGFB_PATHWAY |
| PID_ALK1_PATHWAY |
| PID_WNT_NONCANONICAL_PATHWAY |
| REACTOME_EXTRINSIC_PATHWAY_FOR_APOPTOSIS |
| REACTOME_IMMUNE_SYSTEM |
| KEGG_FOCAL_ADHESION |
| KEGG_CELL_CYCLE |
| module 130 |
| BIOCARTA_TGFB_PATHWAY |
| PID_ALK1_PATHWAY |
| PID_WNT_NONCANONICAL_PATHWAY |
| REACTOME_IMMUNE_SYSTEM |
| REACTOME_CELL_CYCLE_MITOTIC |
| KEGG_APOPTOSIS |
| KEGG_FOCAL_ADHESION |
| module 131 |
| BIOCARTA_TGFB_PATHWAY |
| PID_ALK1_PATHWAY |
| PID_WNT_NONCANONICAL_PATHWAY |
| REACTOME_IMMUNE_SYSTEM |
| REACTOME_G0_AND_EARLY_G1 |
| KEGG_APOPTOSIS |
| KEGG_FOCAL_ADHESION |
| module 132 |
| BIOCARTA_TGFB_PATHWAY |
| PID_ALK1_PATHWAY |
| PID_WNT_NONCANONICAL_PATHWAY |
| REACTOME_IMMUNE_SYSTEM |
| KEGG_APOPTOSIS |
| KEGG_FOCAL_ADHESION |
| KEGG_CELL_CYCLE |
| module 133 |
| BIOCARTA_TGFB_PATHWAY |
| PID_ALK1_PATHWAY |
| REACTOME_APOPTOSIS |
| REACTOME_IMMUNE_SYSTEM |
| REACTOME_CELL_CYCLE_MITOTIC |
| KEGG_FOCAL_ADHESION |
| KEGG_WNT_SIGNALING_PATHWAY |
| module 134 |
| BIOCARTA_TGFB_PATHWAY |
| PID_ALK1_PATHWAY |
| REACTOME_APOPTOSIS |
| REACTOME_IMMUNE_SYSTEM |
| REACTOME_G0_AND_EARLY_G1 |
| KEGG_FOCAL_ADHESION |
| KEGG_WNT_SIGNALING_PATHWAY |
| module 135 |
| BIOCARTA_TGFB_PATHWAY |
| PID_ALK1_PATHWAY |
| REACTOME_APOPTOSIS |
| REACTOME_IMMUNE_SYSTEM |
| KEGG_FOCAL_ADHESION |
| KEGG_WNT_SIGNALING_PATHWAY |
| KEGG_CELL_CYCLE |
| module 136 |
| BIOCARTA_TGFB_PATHWAY |
| PID_ALK1_PATHWAY |
| REACTOME_EXTRINSIC_PATHWAY_FOR_APOPTOSIS |
| REACTOME_IMMUNE_SYSTEM |
| REACTOME_CELL_CYCLE_MITOTIC |
| KEGG_FOCAL_ADHESION |
| KEGG_WNT_SIGNALING_PATHWAY |
| module 137 |
| BIOCARTA_TGFB_PATHWAY |
| PID_ALK1_PATHWAY |
| REACTOME_EXTRINSIC_PATHWAY_FOR_APOPTOSIS |
| REACTOME_IMMUNE_SYSTEM |
| REACTOME_G0_AND_EARLY_G1 |
| KEGG_FOCAL_ADHESION |
| KEGG_WNT_SIGNALING_PATHWAY |
| module 138 |
| BIOCARTA_TGFB_PATHWAY |
| PID_ALK1_PATHWAY |
| REACTOME_EXTRINSIC_PATHWAY_FOR_APOPTOSIS |
| REACTOME_IMMUNE_SYSTEM |
| KEGG_FOCAL_ADHESION |
| KEGG_WNT_SIGNALING_PATHWAY |
| KEGG_CELL_CYCLE |
| module 139 |
| BIOCARTA_TGFB_PATHWAY |
| PID_ALK1_PATHWAY |
| REACTOME_IMMUNE_SYSTEM |
| REACTOME_CELL_CYCLE_MITOTIC |
| KEGG_APOPTOSIS |
| KEGG_FOCAL_ADHESION |
| KEGG_WNT_SIGNALING_PATHWAY |
| module 140 |
| BIOCARTA_TGFB_PATHWAY |
| PID_ALK1_PATHWAY |
| REACTOME_IMMUNE_SYSTEM |
| REACTOME_G0_AND_EARLY_G1 |
| KEGG_APOPTOSIS |
| KEGG_FOCAL_ADHESION |
| KEGG_WNT_SIGNALING_PATHWAY |
| module 141 |
| BIOCARTA_TGFB_PATHWAY |
| PID_ALK1_PATHWAY |
| REACTOME_IMMUNE_SYSTEM |
| KEGG_APOPTOSIS |
| KEGG_FOCAL_ADHESION |
| KEGG_WNT_SIGNALING_PATHWAY |
| KEGG_CELL_CYCLE |
| module 142 |
| BIOCARTA_TGFB_PATHWAY |
| PID_WNT_NONCANONICAL_PATHWAY |
| REACTOME_APOPTOSIS |
| REACTOME_G1_PHASE |
| REACTOME_IMMUNE_SYSTEM |
| REACTOME_SIGNALING_BY_BMP |
| KEGG_FOCAL_ADHESION |
| module 143 |
| BIOCARTA_TGFB_PATHWAY |
| PID_WNT_NONCANONICAL_PATHWAY |
| REACTOME_APOPTOSIS |
| REACTOME_G1_PHASE |
| REACTOME_IMMUNE_SYSTEM |
| PID_ALK2_PATHWAY |
| KEGG_FOCAL_ADHESION |
| module 144 |
| BIOCARTA_TGFB_PATHWAY |
| PID_WNT_NONCANONICAL_PATHWAY |
| REACTOME_APOPTOSIS |
| REACTOME_IMMUNE_SYSTEM |
| REACTOME_CELL_CYCLE_MITOTIC |
| REACTOME_SIGNALING_BY_BMP |
| KEGG_FOCAL_ADHESION |
| module 145 |
| BIOCARTA_TGFB_PATHWAY |
| PID_WNT_NONCANONICAL_PATHWAY |
| REACTOME_APOPTOSIS |
| REACTOME_IMMUNE_SYSTEM |
| REACTOME_CELL_CYCLE_MITOTIC |
| PID_ALK2_PATHWAY |
| KEGG_FOCAL_ADHESION |
| module 146 |
| BIOCARTA_TGFB_PATHWAY |
| PID_WNT_NONCANONICAL_PATHWAY |
| REACTOME_APOPTOSIS |
| REACTOME_IMMUNE_SYSTEM |
| REACTOME_CELL_CYCLE_MITOTIC |
| KEGG_FOCAL_ADHESION |
| KEGG_TGF_BETA_SIGNALING_PATHWAY |
| module 147 |
| BIOCARTA_TGFB_PATHWAY |
| PID_WNT_NONCANONICAL_PATHWAY |
| REACTOME_APOPTOSIS |
| REACTOME_IMMUNE_SYSTEM |
| REACTOME_G0_AND_EARLY_G1 |
| REACTOME_SIGNALING_BY_BMP |
| KEGG_FOCAL_ADHESION |
| module 148 |
| BIOCARTA_TGFB_PATHWAY |
| PID_WNT_NONCANONICAL_PATHWAY |
| REACTOME_APOPTOSIS |
| REACTOME_IMMUNE_SYSTEM |
| REACTOME_G0_AND_EARLY_G1 |
| KEGG_FOCAL_ADHESION |
| KEGG_TGF_BETA_SIGNALING_PATHWAY |
| module 149 |
| BIOCARTA_TGFB_PATHWAY |
| PID_WNT_NONCANONICAL_PATHWAY |
| REACTOME_APOPTOSIS |
| REACTOME_IMMUNE_SYSTEM |
| REACTOME_SIGNALING_BY_BMP |
| KEGG_FOCAL_ADHESION |
| KEGG_CELL_CYCLE |
| module 150 |
| BIOCARTA_TGFB_PATHWAY |
| PID_WNT_NONCANONICAL_PATHWAY |
| REACTOME_APOPTOSIS |
| REACTOME_IMMUNE_SYSTEM |
| PID_ALK2_PATHWAY |
| KEGG_FOCAL_ADHESION |
| KEGG_CELL_CYCLE |
| module 151 |
| BIOCARTA_TGFB_PATHWAY |
| PID_WNT_NONCANONICAL_PATHWAY |
| REACTOME_APOPTOSIS |
| REACTOME_IMMUNE_SYSTEM |
| KEGG_FOCAL_ADHESION |
| KEGG_TGF_BETA_SIGNALING_PATHWAY |
| KEGG_CELL_CYCLE |
| module 152 |
| BIOCARTA_TGFB_PATHWAY |
| PID_WNT_NONCANONICAL_PATHWAY |
| REACTOME_EXTRINSIC_PATHWAY_FOR_APOPTOSIS |
| REACTOME_G1_PHASE |
| REACTOME_IMMUNE_SYSTEM |
| REACTOME_SIGNALING_BY_BMP |
| KEGG_FOCAL_ADHESION |
| module 153 |
| BIOCARTA_TGFB_PATHWAY |
| PID_WNT_NONCANONICAL_PATHWAY |
| REACTOME_EXTRINSIC_PATHWAY_FOR_APOPTOSIS |
| REACTOME_IMMUNE_SYSTEM |
| REACTOME_CELL_CYCLE_MITOTIC |
| REACTOME_SIGNALING_BY_BMP |
| KEGG_FOCAL_ADHESION |
| module 154 |
| BIOCARTA_TGFB_PATHWAY |
| PID_WNT_NONCANONICAL_PATHWAY |
| REACTOME_EXTRINSIC_PATHWAY_FOR_APOPTOSIS |
| REACTOME_IMMUNE_SYSTEM |
| REACTOME_CELL_CYCLE_MITOTIC |
| KEGG_FOCAL_ADHESION |
| KEGG_TGF_BETA_SIGNALING_PATHWAY |
| module 155 |
| BIOCARTA_TGFB_PATHWAY |
| PID_WNT_NONCANONICAL_PATHWAY |
| REACTOME_EXTRINSIC_PATHWAY_FOR_APOPTOSIS |
| REACTOME_IMMUNE_SYSTEM |
| REACTOME_G0_AND_EARLY_G1 |
| REACTOME_SIGNALING_BY_BMP |
| KEGG_FOCAL_ADHESION |
| module 156 |
| BIOCARTA_TGFB_PATHWAY |
| PID_WNT_NONCANONICAL_PATHWAY |
| REACTOME_EXTRINSIC_PATHWAY_FOR_APOPTOSIS |
| REACTOME_IMMUNE_SYSTEM |
| REACTOME_G0_AND_EARLY_G1 |
| KEGG_FOCAL_ADHESION |
| KEGG_TGF_BETA_SIGNALING_PATHWAY |
| module 157 |
| BIOCARTA_TGFB_PATHWAY |
| PID_WNT_NONCANONICAL_PATHWAY |
| REACTOME_EXTRINSIC_PATHWAY_FOR_APOPTOSIS |
| REACTOME_IMMUNE_SYSTEM |
| REACTOME_SIGNALING_BY_BMP |
| KEGG_FOCAL_ADHESION |
| KEGG_CELL_CYCLE |
| module 158 |
| BIOCARTA_TGFB_PATHWAY |
| PID_WNT_NONCANONICAL_PATHWAY |
| REACTOME_EXTRINSIC_PATHWAY_FOR_APOPTOSIS |
| REACTOME_IMMUNE_SYSTEM |
| KEGG_FOCAL_ADHESION |
| KEGG_TGF_BETA_SIGNALING_PATHWAY |
| KEGG_CELL_CYCLE |
| module 159 |
| BIOCARTA_TGFB_PATHWAY |
| PID_WNT_NONCANONICAL_PATHWAY |
| REACTOME_G1_PHASE |
| REACTOME_IMMUNE_SYSTEM |
| REACTOME_SIGNALING_BY_BMP |
| KEGG_APOPTOSIS |
| KEGG_FOCAL_ADHESION |
| module 160 |
| BIOCARTA_TGFB_PATHWAY |
| PID_WNT_NONCANONICAL_PATHWAY |
| REACTOME_G1_PHASE |
| REACTOME_IMMUNE_SYSTEM |
| PID_ALK2_PATHWAY |
| KEGG_APOPTOSIS |
| KEGG_FOCAL_ADHESION |
| module 161 |
| BIOCARTA_TGFB_PATHWAY |
| PID_WNT_NONCANONICAL_PATHWAY |
| REACTOME_IMMUNE_SYSTEM |
| REACTOME_CELL_CYCLE_MITOTIC |
| REACTOME_SIGNALING_BY_BMP |
| KEGG_APOPTOSIS |
| KEGG_FOCAL_ADHESION |
| module 162 |
| BIOCARTA_TGFB_PATHWAY |
| PID_WNT_NONCANONICAL_PATHWAY |
| REACTOME_IMMUNE_SYSTEM |
| REACTOME_CELL_CYCLE_MITOTIC |
| PID_ALK2_PATHWAY |
| KEGG_APOPTOSIS |
| KEGG_FOCAL_ADHESION |
| module 163 |
| BIOCARTA_TGFB_PATHWAY |
| PID_WNT_NONCANONICAL_PATHWAY |
| REACTOME_IMMUNE_SYSTEM |
| REACTOME_CELL_CYCLE_MITOTIC |
| KEGG_APOPTOSIS |
| KEGG_FOCAL_ADHESION |
| KEGG_TGF_BETA_SIGNALING_PATHWAY |
| module 164 |
| BIOCARTA_TGFB_PATHWAY |
| PID_WNT_NONCANONICAL_PATHWAY |
| REACTOME_IMMUNE_SYSTEM |
| REACTOME_G0_AND_EARLY_G1 |
| REACTOME_SIGNALING_BY_BMP |
| KEGG_APOPTOSIS |
| KEGG_FOCAL_ADHESION |
| module 165 |
| BIOCARTA_TGFB_PATHWAY |
| PID_WNT_NONCANONICAL_PATHWAY |
| REACTOME_IMMUNE_SYSTEM |
| REACTOME_G0_AND_EARLY_G1 |
| KEGG_APOPTOSIS |
| KEGG_FOCAL_ADHESION |
| KEGG_TGF_BETA_SIGNALING_PATHWAY |
| module 166 |
| BIOCARTA_TGFB_PATHWAY |
| PID_WNT_NONCANONICAL_PATHWAY |
| REACTOME_IMMUNE_SYSTEM |
| REACTOME_SIGNALING_BY_BMP |
| KEGG_APOPTOSIS |
| KEGG_FOCAL_ADHESION |
| KEGG_CELL_CYCLE |
| module 167 |
| BIOCARTA_TGFB_PATHWAY |
| PID_WNT_NONCANONICAL_PATHWAY |
| REACTOME_IMMUNE_SYSTEM |
| PID_ALK2_PATHWAY |
| KEGG_APOPTOSIS |
| KEGG_FOCAL_ADHESION |
| KEGG_CELL_CYCLE |
| module 168 |
| BIOCARTA_TGFB_PATHWAY |
| PID_WNT_NONCANONICAL_PATHWAY |
| REACTOME_IMMUNE_SYSTEM |
| KEGG_APOPTOSIS |
| KEGG_FOCAL_ADHESION |
| KEGG_TGF_BETA_SIGNALING_PATHWAY |
| KEGG_CELL_CYCLE |
| module 169 |
| BIOCARTA_TGFB_PATHWAY |
| REACTOME_APOPTOSIS |
| REACTOME_G1_PHASE |
| REACTOME_IMMUNE_SYSTEM |
| REACTOME_SIGNALING_BY_BMP |
| KEGG_FOCAL_ADHESION |
| KEGG_WNT_SIGNALING_PATHWAY |
| module 170 |
| BIOCARTA_TGFB_PATHWAY |
| REACTOME_APOPTOSIS |
| REACTOME_G1_PHASE |
| REACTOME_IMMUNE_SYSTEM |
| PID_ALK2_PATHWAY |
| KEGG_FOCAL_ADHESION |
| KEGG_WNT_SIGNALING_PATHWAY |
| module 171 |
| BIOCARTA_TGFB_PATHWAY |
| REACTOME_APOPTOSIS |
| REACTOME_IMMUNE_SYSTEM |
| REACTOME_CELL_CYCLE_MITOTIC |
| REACTOME_SIGNALING_BY_BMP |
| KEGG_FOCAL_ADHESION |
| KEGG_WNT_SIGNALING_PATHWAY |
| module 172 |
| BIOCARTA_TGFB_PATHWAY |
| REACTOME_APOPTOSIS |
| REACTOME_IMMUNE_SYSTEM |
| REACTOME_CELL_CYCLE_MITOTIC |
| PID_ALK2_PATHWAY |
| KEGG_FOCAL_ADHESION |
| KEGG_WNT_SIGNALING_PATHWAY |
| module 173 |
| BIOCARTA_TGFB_PATHWAY |
| REACTOME_APOPTOSIS |
| REACTOME_IMMUNE_SYSTEM |
| REACTOME_CELL_CYCLE_MITOTIC |
| KEGG_FOCAL_ADHESION |
| KEGG_TGF_BETA_SIGNALING_PATHWAY |
| KEGG_WNT_SIGNALING_PATHWAY |
| module 174 |
| BIOCARTA_TGFB_PATHWAY |
| REACTOME_APOPTOSIS |
| REACTOME_IMMUNE_SYSTEM |
| REACTOME_G0_AND_EARLY_G1 |
| REACTOME_SIGNALING_BY_BMP |
| KEGG_FOCAL_ADHESION |
| KEGG_WNT_SIGNALING_PATHWAY |
| module 175 |
| BIOCARTA_TGFB_PATHWAY |
| REACTOME_APOPTOSIS |
| REACTOME_IMMUNE_SYSTEM |
| REACTOME_G0_AND_EARLY_G1 |
| KEGG_FOCAL_ADHESION |
| KEGG_TGF_BETA_SIGNALING_PATHWAY |
| KEGG_WNT_SIGNALING_PATHWAY |
| module 176 |
| BIOCARTA_TGFB_PATHWAY |
| REACTOME_APOPTOSIS |
| REACTOME_IMMUNE_SYSTEM |
| REACTOME_SIGNALING_BY_BMP |
| KEGG_FOCAL_ADHESION |
| KEGG_WNT_SIGNALING_PATHWAY |
| KEGG_CELL_CYCLE |
| module 177 |
| BIOCARTA_TGFB_PATHWAY |
| REACTOME_APOPTOSIS |
| REACTOME_IMMUNE_SYSTEM |
| PID_ALK2_PATHWAY |
| KEGG_FOCAL_ADHESION |
| KEGG_WNT_SIGNALING_PATHWAY |
| KEGG_CELL_CYCLE |
| module 178 |
| BIOCARTA_TGFB_PATHWAY |
| REACTOME_APOPTOSIS |
| REACTOME_IMMUNE_SYSTEM |
| KEGG_FOCAL_ADHESION |
| KEGG_TGF_BETA_SIGNALING_PATHWAY |
| KEGG_WNT_SIGNALING_PATHWAY |
| KEGG_CELL_CYCLE |
| module 179 |
| BIOCARTA_TGFB_PATHWAY |
| REACTOME_EXTRINSIC_PATHWAY_FOR_APOPTOSIS |
| REACTOME_G1_PHASE |
| REACTOME_IMMUNE_SYSTEM |
| REACTOME_SIGNALING_BY_BMP |
| KEGG_FOCAL_ADHESION |
| KEGG_WNT_SIGNALING_PATHWAY |
| module 180 |
| BIOCARTA_TGFB_PATHWAY |
| REACTOME_EXTRINSIC_PATHWAY_FOR_APOPTOSIS |
| REACTOME_IMMUNE_SYSTEM |
| REACTOME_CELL_CYCLE_MITOTIC |
| REACTOME_SIGNALING_BY_BMP |
| KEGG_FOCAL_ADHESION |
| KEGG_WNT_SIGNALING_PATHWAY |
| module 181 |
| BIOCARTA_TGFB_PATHWAY |
| REACTOME_EXTRINSIC_PATHWAY_FOR_APOPTOSIS |
| REACTOME_IMMUNE_SYSTEM |
| REACTOME_CELL_CYCLE_MITOTIC |
| KEGG_FOCAL_ADHESION |
| KEGG_TGF_BETA_SIGNALING_PATHWAY |
| KEGG_WNT_SIGNALING_PATHWAY |
| module 182 |
| BIOCARTA_TGFB_PATHWAY |
| REACTOME_EXTRINSIC_PATHWAY_FOR_APOPTOSIS |
| REACTOME_IMMUNE_SYSTEM |
| REACTOME_G0_AND_EARLY_G1 |
| REACTOME_SIGNALING_BY_BMP |
| KEGG_FOCAL_ADHESION |
| KEGG_WNT_SIGNALING_PATHWAY |
| module 183 |
| BIOCARTA_TGFB_PATHWAY |
| REACTOME_EXTRINSIC_PATHWAY_FOR_APOPTOSIS |
| REACTOME_IMMUNE_SYSTEM |
| REACTOME_G0_AND_EARLY_G1 |
| KEGG_FOCAL_ADHESION |
| KEGG_TGF_BETA_SIGNALING_PATHWAY |
| KEGG_WNT_SIGNALING_PATHWAY |
| module 184 |
| BIOCARTA_TGFB_PATHWAY |
| REACTOME_EXTRINSIC_PATHWAY_FOR_APOPTOSIS |
| REACTOME_IMMUNE_SYSTEM |
| REACTOME_SIGNALING_BY_BMP |
| KEGG_FOCAL_ADHESION |
| KEGG_WNT_SIGNALING_PATHWAY |
| KEGG_CELL_CYCLE |
| module 185 |
| BIOCARTA_TGFB_PATHWAY |
| REACTOME_EXTRINSIC_PATHWAY_FOR_APOPTOSIS |
| REACTOME_IMMUNE_SYSTEM |
| KEGG_FOCAL_ADHESION |
| KEGG_TGF_BETA_SIGNALING_PATHWAY |
| KEGG_WNT_SIGNALING_PATHWAY |
| KEGG_CELL_CYCLE |
| module 186 |
| BIOCARTA_TGFB_PATHWAY |
| REACTOME_G1_PHASE |
| REACTOME_IMMUNE_SYSTEM |
| REACTOME_SIGNALING_BY_BMP |
| KEGG_APOPTOSIS |
| KEGG_FOCAL_ADHESION |
| KEGG_WNT_SIGNALING_PATHWAY |
| module 187 |
| BIOCARTA_TGFB_PATHWAY |
| REACTOME_G1_PHASE |
| REACTOME_IMMUNE_SYSTEM |
| PID_ALK2_PATHWAY |
| KEGG_APOPTOSIS |
| KEGG_FOCAL_ADHESION |
| KEGG_WNT_SIGNALING_PATHWAY |
| module 188 |
| BIOCARTA_TGFB_PATHWAY |
| REACTOME_IMMUNE_SYSTEM |
| REACTOME_CELL_CYCLE_MITOTIC |
| REACTOME_SIGNALING_BY_BMP |
| KEGG_APOPTOSIS |
| KEGG_FOCAL_ADHESION |
| KEGG_WNT_SIGNALING_PATHWAY |
| module 189 |
| BIOCARTA_TGFB_PATHWAY |
| REACTOME_IMMUNE_SYSTEM |
| REACTOME_CELL_CYCLE_MITOTIC |
| PID_ALK2_PATHWAY |
| KEGG_APOPTOSIS |
| KEGG_FOCAL_ADHESION |
| KEGG_WNT_SIGNALING_PATHWAY |
| module 190 |
| BIOCARTA_TGFB_PATHWAY |
| REACTOME_IMMUNE_SYSTEM |
| REACTOME_CELL_CYCLE_MITOTIC |
| KEGG_APOPTOSIS |
| KEGG_FOCAL_ADHESION |
| KEGG_TGF_BETA_SIGNALING_PATHWAY |
| KEGG_WNT_SIGNALING_PATHWAY |
| module 191 |
| BIOCARTA_TGFB_PATHWAY |
| REACTOME_IMMUNE_SYSTEM |
| REACTOME_G0_AND_EARLY_G1 |
| REACTOME_SIGNALING_BY_BMP |
| KEGG_APOPTOSIS |
| KEGG_FOCAL_ADHESION |
| KEGG_WNT_SIGNALING_PATHWAY |
| module 192 |
| BIOCARTA_TGFB_PATHWAY |
| REACTOME_IMMUNE_SYSTEM |
| REACTOME_G0_AND_EARLY_G1 |
| KEGG_APOPTOSIS |
| KEGG_FOCAL_ADHESION |
| KEGG_TGF_BETA_SIGNALING_PATHWAY |
| KEGG_WNT_SIGNALING_PATHWAY |
| module 193 |
| BIOCARTA_TGFB_PATHWAY |
| REACTOME_IMMUNE_SYSTEM |
| REACTOME_SIGNALING_BY_BMP |
| KEGG_APOPTOSIS |
| KEGG_FOCAL_ADHESION |
| KEGG_WNT_SIGNALING_PATHWAY |
| KEGG_CELL_CYCLE |
| module 194 |
| BIOCARTA_TGFB_PATHWAY |
| REACTOME_IMMUNE_SYSTEM |
| PID_ALK2_PATHWAY |
| KEGG_APOPTOSIS |
| KEGG_FOCAL_ADHESION |
| KEGG_WNT_SIGNALING_PATHWAY |
| KEGG_CELL_CYCLE |
| module 195 |
| BIOCARTA_TGFB_PATHWAY |
| REACTOME_IMMUNE_SYSTEM |
| KEGG_APOPTOSIS |
| KEGG_FOCAL_ADHESION |
| KEGG_TGF_BETA_SIGNALING_PATHWAY |
| KEGG_WNT_SIGNALING_PATHWAY |
| KEGG_CELL_CYCLE |
| module 196 |
| BIOCARTA_TGFB_PATHWAY |
| PID_ALK1_PATHWAY |
| PID_WNT_NONCANONICAL_PATHWAY |
| REACTOME_ADAPTIVE_IMMUNE_SYSTEM |
| REACTOME_APOPTOSIS |
| REACTOME_CELL_CYCLE_MITOTIC |
| KEGG_FOCAL_ADHESION |
| module 197 |
| BIOCARTA_TGFB_PATHWAY |
| PID_ALK1_PATHWAY |
| PID_WNT_NONCANONICAL_PATHWAY |
| REACTOME_ADAPTIVE_IMMUNE_SYSTEM |
| REACTOME_EXTRINSIC_PATHWAY_FOR_APOPTOSIS |
| REACTOME_CELL_CYCLE_MITOTIC |
| KEGG_FOCAL_ADHESION |
| module 198 |
| BIOCARTA_TGFB_PATHWAY |
| PID_ALK1_PATHWAY |
| PID_WNT_NONCANONICAL_PATHWAY |
| REACTOME_ADAPTIVE_IMMUNE_SYSTEM |
| REACTOME_CELL_CYCLE_MITOTIC |
| KEGG_APOPTOSIS |
| KEGG_FOCAL_ADHESION |
| module 199 |
| BIOCARTA_TGFB_PATHWAY |
| PID_ALK1_PATHWAY |
| PID_WNT_NONCANONICAL_PATHWAY |
| REACTOME_ANTIGEN_PROCESSING_UBIQUITINATION_PROTEASOME_DEGRADATION |
| REACTOME_APOPTOSIS |
| REACTOME_CELL_CYCLE_MITOTIC |
| KEGG_FOCAL_ADHESION |
| module 200 |
| BIOCARTA_TGFB_PATHWAY |
| PID_ALK1_PATHWAY |
| PID_WNT_NONCANONICAL_PATHWAY |
| REACTOME_ANTIGEN_PROCESSING_UBIQUITINATION_PROTEASOME_DEGRADATION |
| REACTOME_EXTRINSIC_PATHWAY_FOR_APOPTOSIS |
| REACTOME_CELL_CYCLE_MITOTIC |
| KEGG_FOCAL_ADHESION |
| module 201 |
| BIOCARTA_TGFB_PATHWAY |
| PID_ALK1_PATHWAY |
| PID_WNT_NONCANONICAL_PATHWAY |
| REACTOME_ANTIGEN_PROCESSING_UBIQUITINATION_PROTEASOME_DEGRADATION |
| REACTOME_CELL_CYCLE_MITOTIC |
| KEGG_APOPTOSIS |
| KEGG_FOCAL_ADHESION |
| module 202 |
| BIOCARTA_TGFB_PATHWAY |
| PID_ALK1_PATHWAY |
| PID_WNT_NONCANONICAL_PATHWAY |
| REACTOME_APOPTOSIS |
| REACTOME_CLASS_I_MHC_MEDIATED_ANTIGEN_PROCESSING_PRESENTATION |
| REACTOME_CELL_CYCLE_MITOTIC |
| KEGG_FOCAL_ADHESION |
| module 203 |
| BIOCARTA_TGFB_PATHWAY |
| PID_ALK1_PATHWAY |
| PID_WNT_NONCANONICAL_PATHWAY |
| REACTOME_CLASS_I_MHC_MEDIATED_ANTIGEN_PROCESSING_PRESENTATION |
| REACTOME_EXTRINSIC_PATHWAY_FOR_APOPTOSIS |
| REACTOME_CELL_CYCLE_MITOTIC |
| KEGG_FOCAL_ADHESION |
| module 204 |
| BIOCARTA_TGFB_PATHWAY |
| PID_ALK1_PATHWAY |
| PID_WNT_NONCANONICAL_PATHWAY |
| REACTOME_CLASS_I_MHC_MEDIATED_ANTIGEN_PROCESSING_PRESENTATION |
| REACTOME_CELL_CYCLE_MITOTIC |
| KEGG_APOPTOSIS |
| KEGG_FOCAL_ADHESION |
| module 205 |
| BIOCARTA_TGFB_PATHWAY |
| PID_ALK1_PATHWAY |
| REACTOME_ADAPTIVE_IMMUNE_SYSTEM |
| REACTOME_APOPTOSIS |
| REACTOME_CELL_CYCLE_MITOTIC |
| KEGG_FOCAL_ADHESION |
| KEGG_WNT_SIGNALING_PATHWAY |
| module 206 |
| BIOCARTA_TGFB_PATHWAY |
| PID_ALK1_PATHWAY |
| REACTOME_ADAPTIVE_IMMUNE_SYSTEM |
| REACTOME_EXTRINSIC_PATHWAY_FOR_APOPTOSIS |
| REACTOME_CELL_CYCLE_MITOTIC |
| KEGG_FOCAL_ADHESION |
| KEGG_WNT_SIGNALING_PATHWAY |
| module 207 |
| BIOCARTA_TGFB_PATHWAY |
| PID_ALK1_PATHWAY |
| REACTOME_ADAPTIVE_IMMUNE_SYSTEM |
| REACTOME_CELL_CYCLE_MITOTIC |
| KEGG_APOPTOSIS |
| KEGG_FOCAL_ADHESION |
| KEGG_WNT_SIGNALING_PATHWAY |
| module 208 |
| BIOCARTA_TGFB_PATHWAY |
| PID_ALK1_PATHWAY |
| REACTOME_ANTIGEN_PROCESSING_UBIQUITINATION_PROTEASOME_DEGRADATION |
| REACTOME_APOPTOSIS |
| REACTOME_CELL_CYCLE_MITOTIC |
| KEGG_FOCAL_ADHESION |
| KEGG_WNT_SIGNALING_PATHWAY |
| module 209 |
| BIOCARTA_TGFB_PATHWAY |
| PID_ALK1_PATHWAY |
| REACTOME_ANTIGEN_PROCESSING_UBIQUITINATION_PROTEASOME_DEGRADATION |
| REACTOME_EXTRINSIC_PATHWAY_FOR_APOPTOSIS |
| REACTOME_CELL_CYCLE_MITOTIC |
| KEGG_FOCAL_ADHESION |
| KEGG_WNT_SIGNALING_PATHWAY |
| module 210 |
| BIOCARTA_TGFB_PATHWAY |
| PID_ALK1_PATHWAY |
| REACTOME_ANTIGEN_PROCESSING_UBIQUITINATION_PROTEASOME_DEGRADATION |
| REACTOME_CELL_CYCLE_MITOTIC |
| KEGG_APOPTOSIS |
| KEGG_FOCAL_ADHESION |
| KEGG_WNT_SIGNALING_PATHWAY |
| module 211 |
| BIOCARTA_TGFB_PATHWAY |
| PID_ALK1_PATHWAY |
| REACTOME_APOPTOSIS |
| REACTOME_CLASS_I_MHC_MEDIATED_ANTIGEN_PROCESSING_PRESENTATION |
| REACTOME_CELL_CYCLE_MITOTIC |
| KEGG_FOCAL_ADHESION |
| KEGG_WNT_SIGNALING_PATHWAY |
| module 212 |
| BIOCARTA_TGFB_PATHWAY |
| PID_ALK1_PATHWAY |
| REACTOME_CLASS_I_MHC_MEDIATED_ANTIGEN_PROCESSING_PRESENTATION |
| REACTOME_EXTRINSIC_PATHWAY_FOR_APOPTOSIS |
| REACTOME_CELL_CYCLE_MITOTIC |
| KEGG_FOCAL_ADHESION |
| KEGG_WNT_SIGNALING_PATHWAY |
| module 213 |
| BIOCARTA_TGFB_PATHWAY |
| PID_ALK1_PATHWAY |
| REACTOME_CLASS_I_MHC_MEDIATED_ANTIGEN_PROCESSING_PRESENTATION |
| REACTOME_CELL_CYCLE_MITOTIC |
| KEGG_APOPTOSIS |
| KEGG_FOCAL_ADHESION |
| KEGG_WNT_SIGNALING_PATHWAY |
| module 214 |
| BIOCARTA_TGFB_PATHWAY |
| PID_WNT_NONCANONICAL_PATHWAY |
| REACTOME_ADAPTIVE_IMMUNE_SYSTEM |
| REACTOME_APOPTOSIS |
| REACTOME_CELL_CYCLE_MITOTIC |
| REACTOME_SIGNALING_BY_BMP |
| KEGG_FOCAL_ADHESION |
| module 215 |
| BIOCARTA_TGFB_PATHWAY |
| PID_WNT_NONCANONICAL_PATHWAY |
| REACTOME_ADAPTIVE_IMMUNE_SYSTEM |
| REACTOME_APOPTOSIS |
| REACTOME_CELL_CYCLE_MITOTIC |
| PID_ALK2_PATHWAY |
| KEGG_FOCAL_ADHESION |
| module 216 |
| BIOCARTA_TGFB_PATHWAY |
| PID_WNT_NONCANONICAL_PATHWAY |
| REACTOME_ADAPTIVE_IMMUNE_SYSTEM |
| REACTOME_APOPTOSIS |
| REACTOME_CELL_CYCLE_MITOTIC |
| KEGG_FOCAL_ADHESION |
| KEGG_TGF_BETA_SIGNALING_PATHWAY |
| module 217 |
| BIOCARTA_TGFB_PATHWAY |
| PID_WNT_NONCANONICAL_PATHWAY |
| REACTOME_ADAPTIVE_IMMUNE_SYSTEM |
| REACTOME_EXTRINSIC_PATHWAY_FOR_APOPTOSIS |
| REACTOME_CELL_CYCLE_MITOTIC |
| REACTOME_SIGNALING_BY_BMP |
| KEGG_FOCAL_ADHESION |
| module 218 |
| BIOCARTA_TGFB_PATHWAY |
| PID_WNT_NONCANONICAL_PATHWAY |
| REACTOME_ADAPTIVE_IMMUNE_SYSTEM |
| REACTOME_EXTRINSIC_PATHWAY_FOR_APOPTOSIS |
| REACTOME_CELL_CYCLE_MITOTIC |
| KEGG_FOCAL_ADHESION |
| KEGG_TGF_BETA_SIGNALING_PATHWAY |
| module 219 |
| BIOCARTA_TGFB_PATHWAY |
| PID_WNT_NONCANONICAL_PATHWAY |
| REACTOME_ADAPTIVE_IMMUNE_SYSTEM |
| REACTOME_CELL_CYCLE_MITOTIC |
| REACTOME_SIGNALING_BY_BMP |
| KEGG_APOPTOSIS |
| KEGG_FOCAL_ADHESION |
| module 220 |
| BIOCARTA_TGFB_PATHWAY |
| PID_WNT_NONCANONICAL_PATHWAY |
| REACTOME_ADAPTIVE_IMMUNE_SYSTEM |
| REACTOME_CELL_CYCLE_MITOTIC |
| PID_ALK2_PATHWAY |
| KEGG_APOPTOSIS |
| KEGG_FOCAL_ADHESION |
| module 221 |
| BIOCARTA_TGFB_PATHWAY |
| PID_WNT_NONCANONICAL_PATHWAY |
| REACTOME_ADAPTIVE_IMMUNE_SYSTEM |
| REACTOME_CELL_CYCLE_MITOTIC |
| KEGG_APOPTOSIS |
| KEGG_FOCAL_ADHESION |
| KEGG_TGF_BETA_SIGNALING_PATHWAY |
| module 222 |
| BIOCARTA_TGFB_PATHWAY |
| PID_WNT_NONCANONICAL_PATHWAY |
| REACTOME_ANTIGEN_PROCESSING_UBIQUITINATION_PROTEASOME_DEGRADATION |
| REACTOME_APOPTOSIS |
| REACTOME_CELL_CYCLE_MITOTIC |
| REACTOME_SIGNALING_BY_BMP |
| KEGG_FOCAL_ADHESION |
| module 223 |
| BIOCARTA_TGFB_PATHWAY |
| PID_WNT_NONCANONICAL_PATHWAY |
| REACTOME_ANTIGEN_PROCESSING_UBIQUITINATION_PROTEASOME_DEGRADATION |
| REACTOME_APOPTOSIS |
| REACTOME_CELL_CYCLE_MITOTIC |
| PID_ALK2_PATHWAY |
| KEGG_FOCAL_ADHESION |
| module 224 |
| BIOCARTA_TGFB_PATHWAY |
| PID_WNT_NONCANONICAL_PATHWAY |
| REACTOME_ANTIGEN_PROCESSING_UBIQUITINATION_PROTEASOME_DEGRADATION |
| REACTOME_APOPTOSIS |
| REACTOME_CELL_CYCLE_MITOTIC |
| KEGG_FOCAL_ADHESION |
| KEGG_TGF_BETA_SIGNALING_PATHWAY |
| module 225 |
| BIOCARTA_TGFB_PATHWAY |
| PID_WNT_NONCANONICAL_PATHWAY |
| REACTOME_ANTIGEN_PROCESSING_UBIQUITINATION_PROTEASOME_DEGRADATION |
| REACTOME_EXTRINSIC_PATHWAY_FOR_APOPTOSIS |
| REACTOME_CELL_CYCLE_MITOTIC |
| REACTOME_SIGNALING_BY_BMP |
| KEGG_FOCAL_ADHESION |
| module 226 |
| BIOCARTA_TGFB_PATHWAY |
| PID_WNT_NONCANONICAL_PATHWAY |
| REACTOME_ANTIGEN_PROCESSING_UBIQUITINATION_PROTEASOME_DEGRADATION |
| REACTOME_EXTRINSIC_PATHWAY_FOR_APOPTOSIS |
| REACTOME_CELL_CYCLE_MITOTIC |
| KEGG_FOCAL_ADHESION |
| KEGG_TGF_BETA_SIGNALING_PATHWAY |
| module 227 |
| BIOCARTA_TGFB_PATHWAY |
| PID_WNT_NONCANONICAL_PATHWAY |
| REACTOME_ANTIGEN_PROCESSING_UBIQUITINATION_PROTEASOME_DEGRADATION |
| REACTOME_CELL_CYCLE_MITOTIC |
| REACTOME_SIGNALING_BY_BMP |
| KEGG_APOPTOSIS |
| KEGG_FOCAL_ADHESION |
| module 228 |
| BIOCARTA_TGFB_PATHWAY |
| PID_WNT_NONCANONICAL_PATHWAY |
| REACTOME_ANTIGEN_PROCESSING_UBIQUITINATION_PROTEASOME_DEGRADATION |
| REACTOME_CELL_CYCLE_MITOTIC |
| PID_ALK2_PATHWAY |
| KEGG_APOPTOSIS |
| KEGG_FOCAL_ADHESION |
| module 229 |
| BIOCARTA_TGFB_PATHWAY |
| PID_WNT_NONCANONICAL_PATHWAY |
| REACTOME_ANTIGEN_PROCESSING_UBIQUITINATION_PROTEASOME_DEGRADATION |
| REACTOME_CELL_CYCLE_MITOTIC |
| KEGG_APOPTOSIS |
| KEGG_FOCAL_ADHESION |
| KEGG_TGF_BETA_SIGNALING_PATHWAY |
| module 230 |
| BIOCARTA_TGFB_PATHWAY |
| PID_WNT_NONCANONICAL_PATHWAY |
| REACTOME_APOPTOSIS |
| REACTOME_CLASS_I_MHC_MEDIATED_ANTIGEN_PROCESSING_PRESENTATION |
| REACTOME_CELL_CYCLE_MITOTIC |
| REACTOME_SIGNALING_BY_BMP |
| KEGG_FOCAL_ADHESION |
| module 231 |
| BIOCARTA_TGFB_PATHWAY |
| PID_WNT_NONCANONICAL_PATHWAY |
| REACTOME_APOPTOSIS |
| REACTOME_CLASS_I_MHC_MEDIATED_ANTIGEN_PROCESSING_PRESENTATION |
| REACTOME_CELL_CYCLE_MITOTIC |
| PID_ALK2_PATHWAY |
| KEGG_FOCAL_ADHESION |
| module 232 |
| BIOCARTA_TGFB_PATHWAY |
| PID_WNT_NONCANONICAL_PATHWAY |
| REACTOME_APOPTOSIS |
| REACTOME_CLASS_I_MHC_MEDIATED_ANTIGEN_PROCESSING_PRESENTATION |
| REACTOME_CELL_CYCLE_MITOTIC |
| KEGG_FOCAL_ADHESION |
| KEGG_TGF_BETA_SIGNALING_PATHWAY |
| module 233 |
| BIOCARTA_TGFB_PATHWAY |
| PID_WNT_NONCANONICAL_PATHWAY |
| REACTOME_CLASS_I_MHC_MEDIATED_ANTIGEN_PROCESSING_PRESENTATION |
| REACTOME_EXTRINSIC_PATHWAY_FOR_APOPTOSIS |
| REACTOME_CELL_CYCLE_MITOTIC |
| REACTOME_SIGNALING_BY_BMP |
| KEGG_FOCAL_ADHESION |
| module 234 |
| BIOCARTA_TGFB_PATHWAY |
| PID_WNT_NONCANONICAL_PATHWAY |
| REACTOME_CLASS_I_MHC_MEDIATED_ANTIGEN_PROCESSING_PRESENTATION |
| REACTOME_EXTRINSIC_PATHWAY_FOR_APOPTOSIS |
| REACTOME_CELL_CYCLE_MITOTIC |
| KEGG_FOCAL_ADHESION |
| KEGG_TGF_BETA_SIGNALING_PATHWAY |
| module 235 |
| BIOCARTA_TGFB_PATHWAY |
| PID_WNT_NONCANONICAL_PATHWAY |
| REACTOME_CLASS_I_MHC_MEDIATED_ANTIGEN_PROCESSING_PRESENTATION |
| REACTOME_CELL_CYCLE_MITOTIC |
| REACTOME_SIGNALING_BY_BMP |
| KEGG_APOPTOSIS |
| KEGG_FOCAL_ADHESION |
| module 236 |
| BIOCARTA_TGFB_PATHWAY |
| PID_WNT_NONCANONICAL_PATHWAY |
| REACTOME_CLASS_I_MHC_MEDIATED_ANTIGEN_PROCESSING_PRESENTATION |
| REACTOME_CELL_CYCLE_MITOTIC |
| PID_ALK2_PATHWAY |
| KEGG_APOPTOSIS |
| KEGG_FOCAL_ADHESION |
| module 237 |
| BIOCARTA_TGFB_PATHWAY |
| PID_WNT_NONCANONICAL_PATHWAY |
| REACTOME_CLASS_I_MHC_MEDIATED_ANTIGEN_PROCESSING_PRESENTATION |
| REACTOME_CELL_CYCLE_MITOTIC |
| KEGG_APOPTOSIS |
| KEGG_FOCAL_ADHESION |
| KEGG_TGF_BETA_SIGNALING_PATHWAY |
| module 238 |
| BIOCARTA_TGFB_PATHWAY |
| REACTOME_ADAPTIVE_IMMUNE_SYSTEM |
| REACTOME_APOPTOSIS |
| REACTOME_CELL_CYCLE_MITOTIC |
| REACTOME_SIGNALING_BY_BMP |
| KEGG_FOCAL_ADHESION |
| KEGG_WNT_SIGNALING_PATHWAY |
| module 239 |
| BIOCARTA_TGFB_PATHWAY |
| REACTOME_ADAPTIVE_IMMUNE_SYSTEM |
| REACTOME_APOPTOSIS |
| REACTOME_CELL_CYCLE_MITOTIC |
| PID_ALK2_PATHWAY |
| KEGG_FOCAL_ADHESION |
| KEGG_WNT_SIGNALING_PATHWAY |
| module 240 |
| BIOCARTA_TGFB_PATHWAY |
| REACTOME_ADAPTIVE_IMMUNE_SYSTEM |
| REACTOME_APOPTOSIS |
| REACTOME_CELL_CYCLE_MITOTIC |
| KEGG_FOCAL_ADHESION |
| KEGG_TGF_BETA_SIGNALING_PATHWAY |
| KEGG_WNT_SIGNALING_PATHWAY |
| module 241 |
| BIOCARTA_TGFB_PATHWAY |
| REACTOME_ADAPTIVE_IMMUNE_SYSTEM |
| REACTOME_EXTRINSIC_PATHWAY_FOR_APOPTOSIS |
| REACTOME_CELL_CYCLE_MITOTIC |
| REACTOME_SIGNALING_BY_BMP |
| KEGG_FOCAL_ADHESION |
| KEGG_WNT_SIGNALING_PATHWAY |
| module 242 |
| BIOCARTA_TGFB_PATHWAY |
| REACTOME_ADAPTIVE_IMMUNE_SYSTEM |
| REACTOME_EXTRINSIC_PATHWAY_FOR_APOPTOSIS |
| REACTOME_CELL_CYCLE_MITOTIC |
| KEGG_FOCAL_ADHESION |
| KEGG_TGF_BETA_SIGNALING_PATHWAY |
| KEGG_WNT_SIGNALING_PATHWAY |
| module 243 |
| BIOCARTA_TGFB_PATHWAY |
| REACTOME_ADAPTIVE_IMMUNE_SYSTEM |
| REACTOME_CELL_CYCLE_MITOTIC |
| REACTOME_SIGNALING_BY_BMP |
| KEGG_APOPTOSIS |
| KEGG_FOCAL_ADHESION |
| KEGG_WNT_SIGNALING_PATHWAY |
| module 244 |
| BIOCARTA_TGFB_PATHWAY |
| REACTOME_ADAPTIVE_IMMUNE_SYSTEM |
| REACTOME_CELL_CYCLE_MITOTIC |
| PID_ALK2_PATHWAY |
| KEGG_APOPTOSIS |
| KEGG_FOCAL_ADHESION |
| KEGG_WNT_SIGNALING_PATHWAY |
| module 245 |
| BIOCARTA_TGFB_PATHWAY |
| REACTOME_ADAPTIVE_IMMUNE_SYSTEM |
| REACTOME_CELL_CYCLE_MITOTIC |
| KEGG_APOPTOSIS |
| KEGG_FOCAL_ADHESION |
| KEGG_TGF_BETA_SIGNALING_PATHWAY |
| KEGG_WNT_SIGNALING_PATHWAY |
| module 246 |
| BIOCARTA_TGFB_PATHWAY |
| REACTOME_ANTIGEN_PROCESSING_UBIQUITINATION_PROTEASOME_DEGRADATION |
| REACTOME_APOPTOSIS |
| REACTOME_CELL_CYCLE_MITOTIC |
| REACTOME_SIGNALING_BY_BMP |
| KEGG_FOCAL_ADHESION |
| KEGG_WNT_SIGNALING_PATHWAY |
| module 247 |
| BIOCARTA_TGFB_PATHWAY |
| REACTOME_ANTIGEN_PROCESSING_UBIQUITINATION_PROTEASOME_DEGRADATION |
| REACTOME_APOPTOSIS |
| REACTOME_CELL_CYCLE_MITOTIC |
| PID_ALK2_PATHWAY |
| KEGG_FOCAL_ADHESION |
| KEGG_WNT_SIGNALING_PATHWAY |
| module 248 |
| BIOCARTA_TGFB_PATHWAY |
| REACTOME_ANTIGEN_PROCESSING_UBIQUITINATION_PROTEASOME_DEGRADATION |
| REACTOME_APOPTOSIS |
| REACTOME_CELL_CYCLE_MITOTIC |
| KEGG_FOCAL_ADHESION |
| KEGG_TGF_BETA_SIGNALING_PATHWAY |
| KEGG_WNT_SIGNALING_PATHWAY |
| module 249 |
| BIOCARTA_TGFB_PATHWAY |
| REACTOME_ANTIGEN_PROCESSING_UBIQUITINATION_PROTEASOME_DEGRADATION |
| REACTOME_EXTRINSIC_PATHWAY_FOR_APOPTOSIS |
| REACTOME_CELL_CYCLE_MITOTIC |
| REACTOME_SIGNALING_BY_BMP |
| KEGG_FOCAL_ADHESION |
| KEGG_WNT_SIGNALING_PATHWAY |
| module 250 |
| BIOCARTA_TGFB_PATHWAY |
| REACTOME_ANTIGEN_PROCESSING_UBIQUITINATION_PROTEASOME_DEGRADATION |
| REACTOME_EXTRINSIC_PATHWAY_FOR_APOPTOSIS |
| REACTOME_CELL_CYCLE_MITOTIC |
| KEGG_FOCAL_ADHESION |
| KEGG_TGF_BETA_SIGNALING_PATHWAY |
| KEGG_WNT_SIGNALING_PATHWAY |
| module 251 |
| BIOCARTA_TGFB_PATHWAY |
| REACTOME_ANTIGEN_PROCESSING_UBIQUITINATION_PROTEASOME_DEGRADATION |
| REACTOME_CELL_CYCLE_MITOTIC |
| REACTOME_SIGNALING_BY_BMP |
| KEGG_APOPTOSIS |
| KEGG_FOCAL_ADHESION |
| KEGG_WNT_SIGNALING_PATHWAY |
| module 252 |
| BIOCARTA_TGFB_PATHWAY |
| REACTOME_ANTIGEN_PROCESSING_UBIQUITINATION_PROTEASOME_DEGRADATION |
| REACTOME_CELL_CYCLE_MITOTIC |
| PID_ALK2_PATHWAY |
| KEGG_APOPTOSIS |
| KEGG_FOCAL_ADHESION |
| KEGG_WNT_SIGNALING_PATHWAY |
| module 253 |
| BIOCARTA_TGFB_PATHWAY |
| REACTOME_ANTIGEN_PROCESSING_UBIQUITINATION_PROTEASOME_DEGRADATION |
| REACTOME_CELL_CYCLE_MITOTIC |
| KEGG_APOPTOSIS |
| KEGG_FOCAL_ADHESION |
| KEGG_TGF_BETA_SIGNALING_PATHWAY |
| KEGG_WNT_SIGNALING_PATHWAY |
| module 254 |
| BIOCARTA_TGFB_PATHWAY |
| REACTOME_APOPTOSIS |
| REACTOME_CLASS_I_MHC_MEDIATED_ANTIGEN_PROCESSING_PRESENTATION |
| REACTOME_CELL_CYCLE_MITOTIC |
| REACTOME_SIGNALING_BY_BMP |
| KEGG_FOCAL_ADHESION |
| KEGG_WNT_SIGNALING_PATHWAY |
| module 255 |
| BIOCARTA_TGFB_PATHWAY |
| REACTOME_APOPTOSIS |
| REACTOME_CLASS_I_MHC_MEDIATED_ANTIGEN_PROCESSING_PRESENTATION |
| REACTOME_CELL_CYCLE_MITOTIC |
| PID_ALK2_PATHWAY |
| KEGG_FOCAL_ADHESION |
| KEGG_WNT_SIGNALING_PATHWAY |
| module 256 |
| BIOCARTA_TGFB_PATHWAY |
| REACTOME_APOPTOSIS |
| REACTOME_CLASS_I_MHC_MEDIATED_ANTIGEN_PROCESSING_PRESENTATION |
| REACTOME_CELL_CYCLE_MITOTIC |
| KEGG_FOCAL_ADHESION |
| KEGG_TGF_BETA_SIGNALING_PATHWAY |
| KEGG_WNT_SIGNALING_PATHWAY |
| module 257 |
| BIOCARTA_TGFB_PATHWAY |
| REACTOME_CLASS_I_MHC_MEDIATED_ANTIGEN_PROCESSING_PRESENTATION |
| REACTOME_EXTRINSIC_PATHWAY_FOR_APOPTOSIS |
| REACTOME_CELL_CYCLE_MITOTIC |
| REACTOME_SIGNALING_BY_BMP |
| KEGG_FOCAL_ADHESION |
| KEGG_WNT_SIGNALING_PATHWAY |
| module 258 |
| BIOCARTA_TGFB_PATHWAY |
| REACTOME_CLASS_I_MHC_MEDIATED_ANTIGEN_PROCESSING_PRESENTATION |
| REACTOME_EXTRINSIC_PATHWAY_FOR_APOPTOSIS |
| REACTOME_CELL_CYCLE_MITOTIC |
| KEGG_FOCAL_ADHESION |
| KEGG_TGF_BETA_SIGNALING_PATHWAY |
| KEGG_WNT_SIGNALING_PATHWAY |
| module 259 |
| BIOCARTA_TGFB_PATHWAY |
| REACTOME_CLASS_I_MHC_MEDIATED_ANTIGEN_PROCESSING_PRESENTATION |
| REACTOME_CELL_CYCLE_MITOTIC |
| REACTOME_SIGNALING_BY_BMP |
| KEGG_APOPTOSIS |
| KEGG_FOCAL_ADHESION |
| KEGG_WNT_SIGNALING_PATHWAY |
| module 260 |
| BIOCARTA_TGFB_PATHWAY |
| REACTOME_CLASS_I_MHC_MEDIATED_ANTIGEN_PROCESSING_PRESENTATION |
| REACTOME_CELL_CYCLE_MITOTIC |
| PID_ALK2_PATHWAY |
| KEGG_APOPTOSIS |
| KEGG_FOCAL_ADHESION |
| KEGG_WNT_SIGNALING_PATHWAY |
| module 261 |
| BIOCARTA_TGFB_PATHWAY |
| REACTOME_CLASS_I_MHC_MEDIATED_ANTIGEN_PROCESSING_PRESENTATION |
| REACTOME_CELL_CYCLE_MITOTIC |
| KEGG_APOPTOSIS |
| KEGG_FOCAL_ADHESION |
| KEGG_TGF_BETA_SIGNALING_PATHWAY |
| KEGG_WNT_SIGNALING_PATHWAY |
| module 262 |
| BIOCARTA_TGFB_PATHWAY |
| PID_WNT_NONCANONICAL_PATHWAY |
| REACTOME_ADAPTIVE_IMMUNE_SYSTEM |
| REACTOME_APOPTOSIS |
| REACTOME_G1_PHASE |
| REACTOME_DNA_REPLICATION |
| PID_ALK2_PATHWAY |
| KEGG_FOCAL_ADHESION |
| module 263 |
| BIOCARTA_TGFB_PATHWAY |
| PID_WNT_NONCANONICAL_PATHWAY |
| REACTOME_ADAPTIVE_IMMUNE_SYSTEM |
| REACTOME_APOPTOSIS |
| PID_ALK2_PATHWAY |
| KEGG_FOCAL_ADHESION |
| KEGG_CELL_CYCLE |
| module 264 |
| BIOCARTA_TGFB_PATHWAY |
| PID_WNT_NONCANONICAL_PATHWAY |
| REACTOME_ADAPTIVE_IMMUNE_SYSTEM |
| REACTOME_G1_PHASE |
| REACTOME_DNA_REPLICATION |
| PID_ALK2_PATHWAY |
| KEGG_APOPTOSIS |
| KEGG_FOCAL_ADHESION |
| module 265 |
| BIOCARTA_TGFB_PATHWAY |
| PID_WNT_NONCANONICAL_PATHWAY |
| REACTOME_ADAPTIVE_IMMUNE_SYSTEM |
| PID_ALK2_PATHWAY |
| KEGG_APOPTOSIS |
| KEGG_FOCAL_ADHESION |
| KEGG_CELL_CYCLE |
| module 266 |
| BIOCARTA_TGFB_PATHWAY |
| PID_WNT_NONCANONICAL_PATHWAY |
| REACTOME_ANTIGEN_PROCESSING_UBIQUITINATION_PROTEASOME_DEGRADATION |
| REACTOME_APOPTOSIS |
| REACTOME_G1_PHASE |
| REACTOME_DNA_REPLICATION |
| PID_ALK2_PATHWAY |
| KEGG_FOCAL_ADHESION |
| module 267 |
| BIOCARTA_TGFB_PATHWAY |
| PID_WNT_NONCANONICAL_PATHWAY |
| REACTOME_ANTIGEN_PROCESSING_UBIQUITINATION_PROTEASOME_DEGRADATION |
| REACTOME_APOPTOSIS |
| PID_ALK2_PATHWAY |
| KEGG_FOCAL_ADHESION |
| KEGG_CELL_CYCLE |
| module 268 |
| BIOCARTA_TGFB_PATHWAY |
| PID_WNT_NONCANONICAL_PATHWAY |
| REACTOME_ANTIGEN_PROCESSING_UBIQUITINATION_PROTEASOME_DEGRADATION |
| REACTOME_G1_PHASE |
| REACTOME_MITOTIC_M_M_G1_PHASES |
| PID_ALK2_PATHWAY |
| KEGG_APOPTOSIS |
| KEGG_FOCAL_ADHESION |
| module 269 |
| BIOCARTA_TGFB_PATHWAY |
| PID_WNT_NONCANONICAL_PATHWAY |
| REACTOME_ANTIGEN_PROCESSING_UBIQUITINATION_PROTEASOME_DEGRADATION |
| REACTOME_G1_PHASE |
| REACTOME_DNA_REPLICATION |
| PID_ALK2_PATHWAY |
| KEGG_APOPTOSIS |
| KEGG_FOCAL_ADHESION |
| module 270 |
| BIOCARTA_TGFB_PATHWAY |
| PID_WNT_NONCANONICAL_PATHWAY |
| REACTOME_ANTIGEN_PROCESSING_UBIQUITINATION_PROTEASOME_DEGRADATION |
| PID_ALK2_PATHWAY |
| KEGG_APOPTOSIS |
| KEGG_FOCAL_ADHESION |
| KEGG_CELL_CYCLE |
| module 271 |
| BIOCARTA_TGFB_PATHWAY |
| PID_WNT_NONCANONICAL_PATHWAY |
| REACTOME_APOPTOSIS |
| REACTOME_CLASS_I_MHC_MEDIATED_ANTIGEN_PROCESSING_PRESENTATION |
| REACTOME_G1_PHASE |
| REACTOME_DNA_REPLICATION |
| PID_ALK2_PATHWAY |
| KEGG_FOCAL_ADHESION |
| module 272 |
| BIOCARTA_TGFB_PATHWAY |
| PID_WNT_NONCANONICAL_PATHWAY |
| REACTOME_APOPTOSIS |
| REACTOME_CLASS_I_MHC_MEDIATED_ANTIGEN_PROCESSING_PRESENTATION |
| PID_ALK2_PATHWAY |
| KEGG_FOCAL_ADHESION |
| KEGG_CELL_CYCLE |
| module 273 |
| BIOCARTA_TGFB_PATHWAY |
| PID_WNT_NONCANONICAL_PATHWAY |
| REACTOME_CLASS_I_MHC_MEDIATED_ANTIGEN_PROCESSING_PRESENTATION |
| REACTOME_G1_PHASE |
| REACTOME_MITOTIC_M_M_G1_PHASES |
| PID_ALK2_PATHWAY |
| KEGG_APOPTOSIS |
| KEGG_FOCAL_ADHESION |
| module 274 |
| BIOCARTA_TGFB_PATHWAY |
| PID_WNT_NONCANONICAL_PATHWAY |
| REACTOME_CLASS_I_MHC_MEDIATED_ANTIGEN_PROCESSING_PRESENTATION |
| REACTOME_G1_PHASE |
| REACTOME_DNA_REPLICATION |
| PID_ALK2_PATHWAY |
| KEGG_APOPTOSIS |
| KEGG_FOCAL_ADHESION |
| module 275 |
| BIOCARTA_TGFB_PATHWAY |
| PID_WNT_NONCANONICAL_PATHWAY |
| REACTOME_CLASS_I_MHC_MEDIATED_ANTIGEN_PROCESSING_PRESENTATION |
| PID_ALK2_PATHWAY |
| KEGG_APOPTOSIS |
| KEGG_FOCAL_ADHESION |
| KEGG_CELL_CYCLE |
| module 276 |
| BIOCARTA_TGFB_PATHWAY |
| REACTOME_ADAPTIVE_IMMUNE_SYSTEM |
| REACTOME_APOPTOSIS |
| REACTOME_G1_PHASE |
| REACTOME_DNA_REPLICATION |
| PID_ALK2_PATHWAY |
| KEGG_FOCAL_ADHESION |
| KEGG_WNT_SIGNALING_PATHWAY |
| module 277 |
| BIOCARTA_TGFB_PATHWAY |
| REACTOME_ADAPTIVE_IMMUNE_SYSTEM |
| REACTOME_APOPTOSIS |
| PID_ALK2_PATHWAY |
| KEGG_FOCAL_ADHESION |
| KEGG_WNT_SIGNALING_PATHWAY |
| KEGG_CELL_CYCLE |
| module 278 |
| BIOCARTA_TGFB_PATHWAY |
| REACTOME_ADAPTIVE_IMMUNE_SYSTEM |
| REACTOME_G1_PHASE |
| REACTOME_DNA_REPLICATION |
| PID_ALK2_PATHWAY |
| KEGG_APOPTOSIS |
| KEGG_FOCAL_ADHESION |
| KEGG_WNT_SIGNALING_PATHWAY |
| module 279 |
| BIOCARTA_TGFB_PATHWAY |
| REACTOME_ADAPTIVE_IMMUNE_SYSTEM |
| PID_ALK2_PATHWAY |
| KEGG_APOPTOSIS |
| KEGG_FOCAL_ADHESION |
| KEGG_WNT_SIGNALING_PATHWAY |
| KEGG_CELL_CYCLE |
| module 280 |
| BIOCARTA_TGFB_PATHWAY |
| REACTOME_ANTIGEN_PROCESSING_UBIQUITINATION_PROTEASOME_DEGRADATION |
| REACTOME_APOPTOSIS |
| REACTOME_G1_PHASE |
| REACTOME_DNA_REPLICATION |
| PID_ALK2_PATHWAY |
| KEGG_FOCAL_ADHESION |
| KEGG_WNT_SIGNALING_PATHWAY |
| module 281 |
| BIOCARTA_TGFB_PATHWAY |
| REACTOME_ANTIGEN_PROCESSING_UBIQUITINATION_PROTEASOME_DEGRADATION |
| REACTOME_APOPTOSIS |
| PID_ALK2_PATHWAY |
| KEGG_FOCAL_ADHESION |
| KEGG_WNT_SIGNALING_PATHWAY |
| KEGG_CELL_CYCLE |
| module 282 |
| BIOCARTA_TGFB_PATHWAY |
| REACTOME_ANTIGEN_PROCESSING_UBIQUITINATION_PROTEASOME_DEGRADATION |
| REACTOME_G1_PHASE |
| REACTOME_MITOTIC_M_M_G1_PHASES |
| PID_ALK2_PATHWAY |
| KEGG_APOPTOSIS |
| KEGG_FOCAL_ADHESION |
| KEGG_WNT_SIGNALING_PATHWAY |
| module 283 |
| BIOCARTA_TGFB_PATHWAY |
| REACTOME_ANTIGEN_PROCESSING_UBIQUITINATION_PROTEASOME_DEGRADATION |
| REACTOME_G1_PHASE |
| REACTOME_DNA_REPLICATION |
| PID_ALK2_PATHWAY |
| KEGG_APOPTOSIS |
| KEGG_FOCAL_ADHESION |
| KEGG_WNT_SIGNALING_PATHWAY |
| module 284 |
| BIOCARTA_TGFB_PATHWAY |
| REACTOME_ANTIGEN_PROCESSING_UBIQUITINATION_PROTEASOME_DEGRADATION |
| PID_ALK2_PATHWAY |
| KEGG_APOPTOSIS |
| KEGG_FOCAL_ADHESION |
| KEGG_WNT_SIGNALING_PATHWAY |
| KEGG_CELL_CYCLE |
| module 285 |
| BIOCARTA_TGFB_PATHWAY |
| REACTOME_APOPTOSIS |
| REACTOME_CLASS_I_MHC_MEDIATED_ANTIGEN_PROCESSING_PRESENTATION |
| REACTOME_G1_PHASE |
| REACTOME_DNA_REPLICATION |
| PID_ALK2_PATHWAY |
| KEGG_FOCAL_ADHESION |
| KEGG_WNT_SIGNALING_PATHWAY |
| module 286 |
| BIOCARTA_TGFB_PATHWAY |
| REACTOME_APOPTOSIS |
| REACTOME_CLASS_I_MHC_MEDIATED_ANTIGEN_PROCESSING_PRESENTATION |
| PID_ALK2_PATHWAY |
| KEGG_FOCAL_ADHESION |
| KEGG_WNT_SIGNALING_PATHWAY |
| KEGG_CELL_CYCLE |
| module 287 |
| BIOCARTA_TGFB_PATHWAY |
| REACTOME_CLASS_I_MHC_MEDIATED_ANTIGEN_PROCESSING_PRESENTATION |
| REACTOME_G1_PHASE |
| REACTOME_MITOTIC_M_M_G1_PHASES |
| PID_ALK2_PATHWAY |
| KEGG_APOPTOSIS |
| KEGG_FOCAL_ADHESION |
| KEGG_WNT_SIGNALING_PATHWAY |
| module 288 |
| BIOCARTA_TGFB_PATHWAY |
| REACTOME_CLASS_I_MHC_MEDIATED_ANTIGEN_PROCESSING_PRESENTATION |
| REACTOME_G1_PHASE |
| REACTOME_DNA_REPLICATION |
| PID_ALK2_PATHWAY |
| KEGG_APOPTOSIS |
| KEGG_FOCAL_ADHESION |
| KEGG_WNT_SIGNALING_PATHWAY |
| module 289 |
| BIOCARTA_TGFB_PATHWAY |
| REACTOME_CLASS_I_MHC_MEDIATED_ANTIGEN_PROCESSING_PRESENTATION |
| PID_ALK2_PATHWAY |
| KEGG_APOPTOSIS |
| KEGG_FOCAL_ADHESION |
| KEGG_WNT_SIGNALING_PATHWAY |
| KEGG_CELL_CYCLE |
| module 290 |
| BIOCARTA_TGFB_PATHWAY |
| PID_ALK1_PATHWAY |
| PID_WNT_NONCANONICAL_PATHWAY |
| REACTOME_ADAPTIVE_IMMUNE_SYSTEM |
| REACTOME_APOPTOSIS |
| KEGG_FOCAL_ADHESION |
| KEGG_CELL_CYCLE |
| module 291 |
| BIOCARTA_TGFB_PATHWAY |
| PID_ALK1_PATHWAY |
| PID_WNT_NONCANONICAL_PATHWAY |
| REACTOME_ADAPTIVE_IMMUNE_SYSTEM |
| REACTOME_EXTRINSIC_PATHWAY_FOR_APOPTOSIS |
| KEGG_FOCAL_ADHESION |
| KEGG_CELL_CYCLE |
| module 292 |
| BIOCARTA_TGFB_PATHWAY |
| PID_ALK1_PATHWAY |
| PID_WNT_NONCANONICAL_PATHWAY |
| REACTOME_ADAPTIVE_IMMUNE_SYSTEM |
| KEGG_APOPTOSIS |
| KEGG_FOCAL_ADHESION |
| KEGG_CELL_CYCLE |
| module 293 |
| BIOCARTA_TGFB_PATHWAY |
| PID_ALK1_PATHWAY |
| PID_WNT_NONCANONICAL_PATHWAY |
| REACTOME_ANTIGEN_PROCESSING_UBIQUITINATION_PROTEASOME_DEGRADATION |
| REACTOME_APOPTOSIS |
| KEGG_FOCAL_ADHESION |
| KEGG_CELL_CYCLE |
| module 294 |
| BIOCARTA_TGFB_PATHWAY |
| PID_ALK1_PATHWAY |
| PID_WNT_NONCANONICAL_PATHWAY |
| REACTOME_ANTIGEN_PROCESSING_UBIQUITINATION_PROTEASOME_DEGRADATION |
| REACTOME_EXTRINSIC_PATHWAY_FOR_APOPTOSIS |
| KEGG_FOCAL_ADHESION |
| KEGG_CELL_CYCLE |
| module 295 |
| BIOCARTA_TGFB_PATHWAY |
| PID_ALK1_PATHWAY |
| PID_WNT_NONCANONICAL_PATHWAY |
| REACTOME_ANTIGEN_PROCESSING_UBIQUITINATION_PROTEASOME_DEGRADATION |
| KEGG_APOPTOSIS |
| KEGG_FOCAL_ADHESION |
| KEGG_CELL_CYCLE |
| module 296 |
| BIOCARTA_TGFB_PATHWAY |
| PID_ALK1_PATHWAY |
| PID_WNT_NONCANONICAL_PATHWAY |
| REACTOME_APOPTOSIS |
| REACTOME_CLASS_I_MHC_MEDIATED_ANTIGEN_PROCESSING_PRESENTATION |
| KEGG_FOCAL_ADHESION |
| KEGG_CELL_CYCLE |
| module 297 |
| BIOCARTA_TGFB_PATHWAY |
| PID_ALK1_PATHWAY |
| PID_WNT_NONCANONICAL_PATHWAY |
| REACTOME_CLASS_I_MHC_MEDIATED_ANTIGEN_PROCESSING_PRESENTATION |
| REACTOME_EXTRINSIC_PATHWAY_FOR_APOPTOSIS |
| KEGG_FOCAL_ADHESION |
| KEGG_CELL_CYCLE |
| module 298 |
| BIOCARTA_TGFB_PATHWAY |
| PID_ALK1_PATHWAY |
| PID_WNT_NONCANONICAL_PATHWAY |
| REACTOME_CLASS_I_MHC_MEDIATED_ANTIGEN_PROCESSING_PRESENTATION |
| KEGG_APOPTOSIS |
| KEGG_FOCAL_ADHESION |
| KEGG_CELL_CYCLE |
| module 299 |
| BIOCARTA_TGFB_PATHWAY |
| PID_ALK1_PATHWAY |
| REACTOME_ADAPTIVE_IMMUNE_SYSTEM |
| REACTOME_APOPTOSIS |
| KEGG_FOCAL_ADHESION |
| KEGG_WNT_SIGNALING_PATHWAY |
| KEGG_CELL_CYCLE |
| module 300 |
| BIOCARTA_TGFB_PATHWAY |
| PID_ALK1_PATHWAY |
| REACTOME_ADAPTIVE_IMMUNE_SYSTEM |
| REACTOME_EXTRINSIC_PATHWAY_FOR_APOPTOSIS |
| KEGG_FOCAL_ADHESION |
| KEGG_WNT_SIGNALING_PATHWAY |
| KEGG_CELL_CYCLE |
| module 301 |
| BIOCARTA_TGFB_PATHWAY |
| PID_ALK1_PATHWAY |
| REACTOME_ADAPTIVE_IMMUNE_SYSTEM |
| KEGG_APOPTOSIS |
| KEGG_FOCAL_ADHESION |
| KEGG_WNT_SIGNALING_PATHWAY |
| KEGG_CELL_CYCLE |
| module 302 |
| BIOCARTA_TGFB_PATHWAY |
| PID_ALK1_PATHWAY |
| REACTOME_ANTIGEN_PROCESSING_UBIQUITINATION_PROTEASOME_DEGRADATION |
| REACTOME_APOPTOSIS |
| KEGG_FOCAL_ADHESION |
| KEGG_WNT_SIGNALING_PATHWAY |
| KEGG_CELL_CYCLE |
| module 303 |
| BIOCARTA_TGFB_PATHWAY |
| PID_ALK1_PATHWAY |
| REACTOME_ANTIGEN_PROCESSING_UBIQUITINATION_PROTEASOME_DEGRADATION |
| REACTOME_EXTRINSIC_PATHWAY_FOR_APOPTOSIS |
| KEGG_FOCAL_ADHESION |
| KEGG_WNT_SIGNALING_PATHWAY |
| KEGG_CELL_CYCLE |
| module 304 |
| BIOCARTA_TGFB_PATHWAY |
| PID_ALK1_PATHWAY |
| REACTOME_ANTIGEN_PROCESSING_UBIQUITINATION_PROTEASOME_DEGRADATION |
| KEGG_APOPTOSIS |
| KEGG_FOCAL_ADHESION |
| KEGG_WNT_SIGNALING_PATHWAY |
| KEGG_CELL_CYCLE |
| module 305 |
| BIOCARTA_TGFB_PATHWAY |
| PID_ALK1_PATHWAY |
| REACTOME_APOPTOSIS |
| REACTOME_CLASS_I_MHC_MEDIATED_ANTIGEN_PROCESSING_PRESENTATION |
| KEGG_FOCAL_ADHESION |
| KEGG_WNT_SIGNALING_PATHWAY |
| KEGG_CELL_CYCLE |
| module 306 |
| BIOCARTA_TGFB_PATHWAY |
| PID_ALK1_PATHWAY |
| REACTOME_CLASS_I_MHC_MEDIATED_ANTIGEN_PROCESSING_PRESENTATION |
| REACTOME_EXTRINSIC_PATHWAY_FOR_APOPTOSIS |
| KEGG_FOCAL_ADHESION |
| KEGG_WNT_SIGNALING_PATHWAY |
| KEGG_CELL_CYCLE |
| module 307 |
| BIOCARTA_TGFB_PATHWAY |
| PID_ALK1_PATHWAY |
| REACTOME_CLASS_I_MHC_MEDIATED_ANTIGEN_PROCESSING_PRESENTATION |
| KEGG_APOPTOSIS |
| KEGG_FOCAL_ADHESION |
| KEGG_WNT_SIGNALING_PATHWAY |
| KEGG_CELL_CYCLE |
| module 308 |
| BIOCARTA_TGFB_PATHWAY |
| PID_WNT_NONCANONICAL_PATHWAY |
| REACTOME_ADAPTIVE_IMMUNE_SYSTEM |
| REACTOME_APOPTOSIS |
| REACTOME_SIGNALING_BY_BMP |
| KEGG_FOCAL_ADHESION |
| KEGG_CELL_CYCLE |
| module 309 |
| BIOCARTA_TGFB_PATHWAY |
| PID_WNT_NONCANONICAL_PATHWAY |
| REACTOME_ADAPTIVE_IMMUNE_SYSTEM |
| REACTOME_APOPTOSIS |
| KEGG_FOCAL_ADHESION |
| KEGG_TGF_BETA_SIGNALING_PATHWAY |
| KEGG_CELL_CYCLE |
| module 310 |
| BIOCARTA_TGFB_PATHWAY |
| PID_WNT_NONCANONICAL_PATHWAY |
| REACTOME_ADAPTIVE_IMMUNE_SYSTEM |
| REACTOME_EXTRINSIC_PATHWAY_FOR_APOPTOSIS |
| REACTOME_SIGNALING_BY_BMP |
| KEGG_FOCAL_ADHESION |
| KEGG_CELL_CYCLE |
| module 311 |
| BIOCARTA_TGFB_PATHWAY |
| PID_WNT_NONCANONICAL_PATHWAY |
| REACTOME_ADAPTIVE_IMMUNE_SYSTEM |
| REACTOME_EXTRINSIC_PATHWAY_FOR_APOPTOSIS |
| KEGG_FOCAL_ADHESION |
| KEGG_TGF_BETA_SIGNALING_PATHWAY |
| KEGG_CELL_CYCLE |
| module 312 |
| BIOCARTA_TGFB_PATHWAY |
| PID_WNT_NONCANONICAL_PATHWAY |
| REACTOME_ADAPTIVE_IMMUNE_SYSTEM |
| REACTOME_SIGNALING_BY_BMP |
| KEGG_APOPTOSIS |
| KEGG_FOCAL_ADHESION |
| KEGG_CELL_CYCLE |
| module 313 |
| BIOCARTA_TGFB_PATHWAY |
| PID_WNT_NONCANONICAL_PATHWAY |
| REACTOME_ADAPTIVE_IMMUNE_SYSTEM |
| KEGG_APOPTOSIS |
| KEGG_FOCAL_ADHESION |
| KEGG_TGF_BETA_SIGNALING_PATHWAY |
| KEGG_CELL_CYCLE |
| module 314 |
| BIOCARTA_TGFB_PATHWAY |
| PID_WNT_NONCANONICAL_PATHWAY |
| REACTOME_ANTIGEN_PROCESSING_UBIQUITINATION_PROTEASOME_DEGRADATION |
| REACTOME_APOPTOSIS |
| REACTOME_SIGNALING_BY_BMP |
| KEGG_FOCAL_ADHESION |
| KEGG_CELL_CYCLE |
| module 315 |
| BIOCARTA_TGFB_PATHWAY |
| PID_WNT_NONCANONICAL_PATHWAY |
| REACTOME_ANTIGEN_PROCESSING_UBIQUITINATION_PROTEASOME_DEGRADATION |
| REACTOME_APOPTOSIS |
| KEGG_FOCAL_ADHESION |
| KEGG_TGF_BETA_SIGNALING_PATHWAY |
| KEGG_CELL_CYCLE |
| module 316 |
| BIOCARTA_TGFB_PATHWAY |
| PID_WNT_NONCANONICAL_PATHWAY |
| REACTOME_ANTIGEN_PROCESSING_UBIQUITINATION_PROTEASOME_DEGRADATION |
| REACTOME_EXTRINSIC_PATHWAY_FOR_APOPTOSIS |
| REACTOME_SIGNALING_BY_BMP |
| KEGG_FOCAL_ADHESION |
| KEGG_CELL_CYCLE |
| module 317 |
| BIOCARTA_TGFB_PATHWAY |
| PID_WNT_NONCANONICAL_PATHWAY |
| REACTOME_ANTIGEN_PROCESSING_UBIQUITINATION_PROTEASOME_DEGRADATION |
| REACTOME_EXTRINSIC_PATHWAY_FOR_APOPTOSIS |
| KEGG_FOCAL_ADHESION |
| KEGG_TGF_BETA_SIGNALING_PATHWAY |
| KEGG_CELL_CYCLE |
| module 318 |
| BIOCARTA_TGFB_PATHWAY |
| PID_WNT_NONCANONICAL_PATHWAY |
| REACTOME_ANTIGEN_PROCESSING_UBIQUITINATION_PROTEASOME_DEGRADATION |
| REACTOME_SIGNALING_BY_BMP |
| KEGG_APOPTOSIS |
| KEGG_FOCAL_ADHESION |
| KEGG_CELL_CYCLE |
| module 319 |
| BIOCARTA_TGFB_PATHWAY |
| PID_WNT_NONCANONICAL_PATHWAY |
| REACTOME_ANTIGEN_PROCESSING_UBIQUITINATION_PROTEASOME_DEGRADATION |
| KEGG_APOPTOSIS |
| KEGG_FOCAL_ADHESION |
| KEGG_TGF_BETA_SIGNALING_PATHWAY |
| KEGG_CELL_CYCLE |
| module 320 |
| BIOCARTA_TGFB_PATHWAY |
| PID_WNT_NONCANONICAL_PATHWAY |
| REACTOME_APOPTOSIS |
| REACTOME_CLASS_I_MHC_MEDIATED_ANTIGEN_PROCESSING_PRESENTATION |
| REACTOME_SIGNALING_BY_BMP |
| KEGG_FOCAL_ADHESION |
| KEGG_CELL_CYCLE |
| module 321 |
| BIOCARTA_TGFB_PATHWAY |
| PID_WNT_NONCANONICAL_PATHWAY |
| REACTOME_APOPTOSIS |
| REACTOME_CLASS_I_MHC_MEDIATED_ANTIGEN_PROCESSING_PRESENTATION |
| KEGG_FOCAL_ADHESION |
| KEGG_TGF_BETA_SIGNALING_PATHWAY |
| KEGG_CELL_CYCLE |
| module 322 |
| BIOCARTA_TGFB_PATHWAY |
| PID_WNT_NONCANONICAL_PATHWAY |
| REACTOME_CLASS_I_MHC_MEDIATED_ANTIGEN_PROCESSING_PRESENTATION |
| REACTOME_EXTRINSIC_PATHWAY_FOR_APOPTOSIS |
| REACTOME_SIGNALING_BY_BMP |
| KEGG_FOCAL_ADHESION |
| KEGG_CELL_CYCLE |
| module 323 |
| BIOCARTA_TGFB_PATHWAY |
| PID_WNT_NONCANONICAL_PATHWAY |
| REACTOME_CLASS_I_MHC_MEDIATED_ANTIGEN_PROCESSING_PRESENTATION |
| REACTOME_EXTRINSIC_PATHWAY_FOR_APOPTOSIS |
| KEGG_FOCAL_ADHESION |
| KEGG_TGF_BETA_SIGNALING_PATHWAY |
| KEGG_CELL_CYCLE |
| module 324 |
| BIOCARTA_TGFB_PATHWAY |
| PID_WNT_NONCANONICAL_PATHWAY |
| REACTOME_CLASS_I_MHC_MEDIATED_ANTIGEN_PROCESSING_PRESENTATION |
| REACTOME_SIGNALING_BY_BMP |
| KEGG_APOPTOSIS |
| KEGG_FOCAL_ADHESION |
| KEGG_CELL_CYCLE |
| module 325 |
| BIOCARTA_TGFB_PATHWAY |
| PID_WNT_NONCANONICAL_PATHWAY |
| REACTOME_CLASS_I_MHC_MEDIATED_ANTIGEN_PROCESSING_PRESENTATION |
| KEGG_APOPTOSIS |
| KEGG_FOCAL_ADHESION |
| KEGG_TGF_BETA_SIGNALING_PATHWAY |
| KEGG_CELL_CYCLE |
| module 326 |
| BIOCARTA_TGFB_PATHWAY |
| REACTOME_ADAPTIVE_IMMUNE_SYSTEM |
| REACTOME_APOPTOSIS |
| REACTOME_SIGNALING_BY_BMP |
| KEGG_FOCAL_ADHESION |
| KEGG_WNT_SIGNALING_PATHWAY |
| KEGG_CELL_CYCLE |
| module 327 |
| BIOCARTA_TGFB_PATHWAY |
| REACTOME_ADAPTIVE_IMMUNE_SYSTEM |
| REACTOME_APOPTOSIS |
| KEGG_FOCAL_ADHESION |
| KEGG_TGF_BETA_SIGNALING_PATHWAY |
| KEGG_WNT_SIGNALING_PATHWAY |
| KEGG_CELL_CYCLE |
| module 328 |
| BIOCARTA_TGFB_PATHWAY |
| REACTOME_ADAPTIVE_IMMUNE_SYSTEM |
| REACTOME_EXTRINSIC_PATHWAY_FOR_APOPTOSIS |
| REACTOME_SIGNALING_BY_BMP |
| KEGG_FOCAL_ADHESION |
| KEGG_WNT_SIGNALING_PATHWAY |
| KEGG_CELL_CYCLE |
| module 329 |
| BIOCARTA_TGFB_PATHWAY |
| REACTOME_ADAPTIVE_IMMUNE_SYSTEM |
| REACTOME_EXTRINSIC_PATHWAY_FOR_APOPTOSIS |
| KEGG_FOCAL_ADHESION |
| KEGG_TGF_BETA_SIGNALING_PATHWAY |
| KEGG_WNT_SIGNALING_PATHWAY |
| KEGG_CELL_CYCLE |
| module 330 |
| BIOCARTA_TGFB_PATHWAY |
| REACTOME_ADAPTIVE_IMMUNE_SYSTEM |
| REACTOME_SIGNALING_BY_BMP |
| KEGG_APOPTOSIS |
| KEGG_FOCAL_ADHESION |
| KEGG_WNT_SIGNALING_PATHWAY |
| KEGG_CELL_CYCLE |
| module 331 |
| BIOCARTA_TGFB_PATHWAY |
| REACTOME_ADAPTIVE_IMMUNE_SYSTEM |
| KEGG_APOPTOSIS |
| KEGG_FOCAL_ADHESION |
| KEGG_TGF_BETA_SIGNALING_PATHWAY |
| KEGG_WNT_SIGNALING_PATHWAY |
| KEGG_CELL_CYCLE |
| module 332 |
| BIOCARTA_TGFB_PATHWAY |
| REACTOME_ANTIGEN_PROCESSING_UBIQUITINATION_PROTEASOME_DEGRADATION |
| REACTOME_APOPTOSIS |
| REACTOME_SIGNALING_BY_BMP |
| KEGG_FOCAL_ADHESION |
| KEGG_WNT_SIGNALING_PATHWAY |
| KEGG_CELL_CYCLE |
| module 333 |
| BIOCARTA_TGFB_PATHWAY |
| REACTOME_ANTIGEN_PROCESSING_UBIQUITINATION_PROTEASOME_DEGRADATION |
| REACTOME_APOPTOSIS |
| KEGG_FOCAL_ADHESION |
| KEGG_TGF_BETA_SIGNALING_PATHWAY |
| KEGG_WNT_SIGNALING_PATHWAY |
| KEGG_CELL_CYCLE |
| module 334 |
| BIOCARTA_TGFB_PATHWAY |
| REACTOME_ANTIGEN_PROCESSING_UBIQUITINATION_PROTEASOME_DEGRADATION |
| REACTOME_EXTRINSIC_PATHWAY_FOR_APOPTOSIS |
| REACTOME_SIGNALING_BY_BMP |
| KEGG_FOCAL_ADHESION |
| KEGG_WNT_SIGNALING_PATHWAY |
| KEGG_CELL_CYCLE |
| module 335 |
| BIOCARTA_TGFB_PATHWAY |
| REACTOME_ANTIGEN_PROCESSING_UBIQUITINATION_PROTEASOME_DEGRADATION |
| REACTOME_EXTRINSIC_PATHWAY_FOR_APOPTOSIS |
| KEGG_FOCAL_ADHESION |
| KEGG_TGF_BETA_SIGNALING_PATHWAY |
| KEGG_WNT_SIGNALING_PATHWAY |
| KEGG_CELL_CYCLE |
| module 336 |
| BIOCARTA_TGFB_PATHWAY |
| REACTOME_ANTIGEN_PROCESSING_UBIQUITINATION_PROTEASOME_DEGRADATION |
| REACTOME_SIGNALING_BY_BMP |
| KEGG_APOPTOSIS |
| KEGG_FOCAL_ADHESION |
| KEGG_WNT_SIGNALING_PATHWAY |
| KEGG_CELL_CYCLE |
| module 337 |
| BIOCARTA_TGFB_PATHWAY |
| REACTOME_ANTIGEN_PROCESSING_UBIQUITINATION_PROTEASOME_DEGRADATION |
| KEGG_APOPTOSIS |
| KEGG_FOCAL_ADHESION |
| KEGG_TGF_BETA_SIGNALING_PATHWAY |
| KEGG_WNT_SIGNALING_PATHWAY |
| KEGG_CELL_CYCLE |
| module 338 |
| BIOCARTA_TGFB_PATHWAY |
| REACTOME_APOPTOSIS |
| REACTOME_CLASS_I_MHC_MEDIATED_ANTIGEN_PROCESSING_PRESENTATION |
| REACTOME_SIGNALING_BY_BMP |
| KEGG_FOCAL_ADHESION |
| KEGG_WNT_SIGNALING_PATHWAY |
| KEGG_CELL_CYCLE |
| module 339 |
| BIOCARTA_TGFB_PATHWAY |
| REACTOME_APOPTOSIS |
| REACTOME_CLASS_I_MHC_MEDIATED_ANTIGEN_PROCESSING_PRESENTATION |
| KEGG_FOCAL_ADHESION |
| KEGG_TGF_BETA_SIGNALING_PATHWAY |
| KEGG_WNT_SIGNALING_PATHWAY |
| KEGG_CELL_CYCLE |
| module 340 |
| BIOCARTA_TGFB_PATHWAY |
| REACTOME_CLASS_I_MHC_MEDIATED_ANTIGEN_PROCESSING_PRESENTATION |
| REACTOME_EXTRINSIC_PATHWAY_FOR_APOPTOSIS |
| REACTOME_SIGNALING_BY_BMP |
| KEGG_FOCAL_ADHESION |
| KEGG_WNT_SIGNALING_PATHWAY |
| KEGG_CELL_CYCLE |
| module 341 |
| BIOCARTA_TGFB_PATHWAY |
| REACTOME_CLASS_I_MHC_MEDIATED_ANTIGEN_PROCESSING_PRESENTATION |
| REACTOME_EXTRINSIC_PATHWAY_FOR_APOPTOSIS |
| KEGG_FOCAL_ADHESION |
| KEGG_TGF_BETA_SIGNALING_PATHWAY |
| KEGG_WNT_SIGNALING_PATHWAY |
| KEGG_CELL_CYCLE |
| module 342 |
| BIOCARTA_TGFB_PATHWAY |
| REACTOME_CLASS_I_MHC_MEDIATED_ANTIGEN_PROCESSING_PRESENTATION |
| REACTOME_SIGNALING_BY_BMP |
| KEGG_APOPTOSIS |
| KEGG_FOCAL_ADHESION |
| KEGG_WNT_SIGNALING_PATHWAY |
| KEGG_CELL_CYCLE |
| module 343 |
| BIOCARTA_TGFB_PATHWAY |
| REACTOME_CLASS_I_MHC_MEDIATED_ANTIGEN_PROCESSING_PRESENTATION |
| KEGG_APOPTOSIS |
| KEGG_FOCAL_ADHESION |
| KEGG_TGF_BETA_SIGNALING_PATHWAY |
| KEGG_WNT_SIGNALING_PATHWAY |
| KEGG_CELL_CYCLE |
| module 344 |
| BIOCARTA_TGFB_PATHWAY |
| PID_ALK1_PATHWAY |
| PID_WNT_NONCANONICAL_PATHWAY |
| REACTOME_ADAPTIVE_IMMUNE_SYSTEM |
| REACTOME_APOPTOSIS |
| REACTOME_G1_PHASE |
| REACTOME_DNA_REPLICATION |
| KEGG_FOCAL_ADHESION |
| module 345 |
| BIOCARTA_TGFB_PATHWAY |
| PID_ALK1_PATHWAY |
| PID_WNT_NONCANONICAL_PATHWAY |
| REACTOME_ADAPTIVE_IMMUNE_SYSTEM |
| REACTOME_APOPTOSIS |
| REACTOME_DNA_REPLICATION |
| REACTOME_G0_AND_EARLY_G1 |
| KEGG_FOCAL_ADHESION |
| module 346 |
| BIOCARTA_TGFB_PATHWAY |
| PID_ALK1_PATHWAY |
| PID_WNT_NONCANONICAL_PATHWAY |
| REACTOME_ADAPTIVE_IMMUNE_SYSTEM |
| REACTOME_EXTRINSIC_PATHWAY_FOR_APOPTOSIS |
| REACTOME_G1_PHASE |
| REACTOME_DNA_REPLICATION |
| KEGG_FOCAL_ADHESION |
| module 347 |
| BIOCARTA_TGFB_PATHWAY |
| PID_ALK1_PATHWAY |
| PID_WNT_NONCANONICAL_PATHWAY |
| REACTOME_ADAPTIVE_IMMUNE_SYSTEM |
| REACTOME_EXTRINSIC_PATHWAY_FOR_APOPTOSIS |
| REACTOME_DNA_REPLICATION |
| REACTOME_G0_AND_EARLY_G1 |
| KEGG_FOCAL_ADHESION |
| module 348 |
| BIOCARTA_TGFB_PATHWAY |
| PID_ALK1_PATHWAY |
| PID_WNT_NONCANONICAL_PATHWAY |
| REACTOME_ADAPTIVE_IMMUNE_SYSTEM |
| REACTOME_G1_PHASE |
| REACTOME_DNA_REPLICATION |
| KEGG_APOPTOSIS |
| KEGG_FOCAL_ADHESION |
| module 349 |
| BIOCARTA_TGFB_PATHWAY |
| PID_ALK1_PATHWAY |
| PID_WNT_NONCANONICAL_PATHWAY |
| REACTOME_ADAPTIVE_IMMUNE_SYSTEM |
| REACTOME_DNA_REPLICATION |
| REACTOME_G0_AND_EARLY_G1 |
| KEGG_APOPTOSIS |
| KEGG_FOCAL_ADHESION |
| module 350 |
| BIOCARTA_TGFB_PATHWAY |
| PID_ALK1_PATHWAY |
| REACTOME_ADAPTIVE_IMMUNE_SYSTEM |
| REACTOME_APOPTOSIS |
| REACTOME_G1_PHASE |
| REACTOME_DNA_REPLICATION |
| KEGG_FOCAL_ADHESION |
| KEGG_WNT_SIGNALING_PATHWAY |
| module 351 |
| BIOCARTA_TGFB_PATHWAY |
| PID_ALK1_PATHWAY |
| REACTOME_ADAPTIVE_IMMUNE_SYSTEM |
| REACTOME_APOPTOSIS |
| REACTOME_DNA_REPLICATION |
| REACTOME_G0_AND_EARLY_G1 |
| KEGG_FOCAL_ADHESION |
| KEGG_WNT_SIGNALING_PATHWAY |
| module 352 |
| BIOCARTA_TGFB_PATHWAY |
| PID_ALK1_PATHWAY |
| REACTOME_ADAPTIVE_IMMUNE_SYSTEM |
| REACTOME_EXTRINSIC_PATHWAY_FOR_APOPTOSIS |
| REACTOME_G1_PHASE |
| REACTOME_DNA_REPLICATION |
| KEGG_FOCAL_ADHESION |
| KEGG_WNT_SIGNALING_PATHWAY |
| module 353 |
| BIOCARTA_TGFB_PATHWAY |
| PID_ALK1_PATHWAY |
| REACTOME_ADAPTIVE_IMMUNE_SYSTEM |
| REACTOME_EXTRINSIC_PATHWAY_FOR_APOPTOSIS |
| REACTOME_DNA_REPLICATION |
| REACTOME_G0_AND_EARLY_G1 |
| KEGG_FOCAL_ADHESION |
| KEGG_WNT_SIGNALING_PATHWAY |
| module 354 |
| BIOCARTA_TGFB_PATHWAY |
| PID_ALK1_PATHWAY |
| REACTOME_ADAPTIVE_IMMUNE_SYSTEM |
| REACTOME_G1_PHASE |
| REACTOME_DNA_REPLICATION |
| KEGG_APOPTOSIS |
| KEGG_FOCAL_ADHESION |
| KEGG_WNT_SIGNALING_PATHWAY |
| module 355 |
| BIOCARTA_TGFB_PATHWAY |
| PID_ALK1_PATHWAY |
| REACTOME_ADAPTIVE_IMMUNE_SYSTEM |
| REACTOME_DNA_REPLICATION |
| REACTOME_G0_AND_EARLY_G1 |
| KEGG_APOPTOSIS |
| KEGG_FOCAL_ADHESION |
| KEGG_WNT_SIGNALING_PATHWAY |
| module 356 |
| BIOCARTA_TGFB_PATHWAY |
| PID_WNT_NONCANONICAL_PATHWAY |
| REACTOME_ADAPTIVE_IMMUNE_SYSTEM |
| REACTOME_APOPTOSIS |
| REACTOME_G1_PHASE |
| REACTOME_DNA_REPLICATION |
| REACTOME_SIGNALING_BY_BMP |
| KEGG_FOCAL_ADHESION |
| module 357 |
| BIOCARTA_TGFB_PATHWAY |
| PID_WNT_NONCANONICAL_PATHWAY |
| REACTOME_ADAPTIVE_IMMUNE_SYSTEM |
| REACTOME_APOPTOSIS |
| REACTOME_G1_PHASE |
| REACTOME_DNA_REPLICATION |
| KEGG_FOCAL_ADHESION |
| KEGG_TGF_BETA_SIGNALING_PATHWAY |
| module 358 |
| BIOCARTA_TGFB_PATHWAY |
| PID_WNT_NONCANONICAL_PATHWAY |
| REACTOME_ADAPTIVE_IMMUNE_SYSTEM |
| REACTOME_APOPTOSIS |
| REACTOME_DNA_REPLICATION |
| REACTOME_G0_AND_EARLY_G1 |
| REACTOME_SIGNALING_BY_BMP |
| KEGG_FOCAL_ADHESION |
| module 359 |
| BIOCARTA_TGFB_PATHWAY |
| PID_WNT_NONCANONICAL_PATHWAY |
| REACTOME_ADAPTIVE_IMMUNE_SYSTEM |
| REACTOME_APOPTOSIS |
| REACTOME_DNA_REPLICATION |
| REACTOME_G0_AND_EARLY_G1 |
| KEGG_FOCAL_ADHESION |
| KEGG_TGF_BETA_SIGNALING_PATHWAY |
| module 360 |
| BIOCARTA_TGFB_PATHWAY |
| PID_WNT_NONCANONICAL_PATHWAY |
| REACTOME_ADAPTIVE_IMMUNE_SYSTEM |
| REACTOME_EXTRINSIC_PATHWAY_FOR_APOPTOSIS |
| REACTOME_G1_PHASE |
| REACTOME_DNA_REPLICATION |
| REACTOME_SIGNALING_BY_BMP |
| KEGG_FOCAL_ADHESION |
| module 361 |
| BIOCARTA_TGFB_PATHWAY |
| PID_WNT_NONCANONICAL_PATHWAY |
| REACTOME_ADAPTIVE_IMMUNE_SYSTEM |
| REACTOME_EXTRINSIC_PATHWAY_FOR_APOPTOSIS |
| REACTOME_G1_PHASE |
| REACTOME_DNA_REPLICATION |
| KEGG_FOCAL_ADHESION |
| KEGG_TGF_BETA_SIGNALING_PATHWAY |
| module 362 |
| BIOCARTA_TGFB_PATHWAY |
| PID_WNT_NONCANONICAL_PATHWAY |
| REACTOME_ADAPTIVE_IMMUNE_SYSTEM |
| REACTOME_EXTRINSIC_PATHWAY_FOR_APOPTOSIS |
| REACTOME_DNA_REPLICATION |
| REACTOME_G0_AND_EARLY_G1 |
| REACTOME_SIGNALING_BY_BMP |
| KEGG_FOCAL_ADHESION |
| module 363 |
| BIOCARTA_TGFB_PATHWAY |
| PID_WNT_NONCANONICAL_PATHWAY |
| REACTOME_ADAPTIVE_IMMUNE_SYSTEM |
| REACTOME_EXTRINSIC_PATHWAY_FOR_APOPTOSIS |
| REACTOME_DNA_REPLICATION |
| REACTOME_G0_AND_EARLY_G1 |
| KEGG_FOCAL_ADHESION |
| KEGG_TGF_BETA_SIGNALING_PATHWAY |
| module 364 |
| BIOCARTA_TGFB_PATHWAY |
| PID_WNT_NONCANONICAL_PATHWAY |
| REACTOME_ADAPTIVE_IMMUNE_SYSTEM |
| REACTOME_G1_PHASE |
| REACTOME_DNA_REPLICATION |
| REACTOME_SIGNALING_BY_BMP |
| KEGG_APOPTOSIS |
| KEGG_FOCAL_ADHESION |
| module 365 |
| BIOCARTA_TGFB_PATHWAY |
| PID_WNT_NONCANONICAL_PATHWAY |
| REACTOME_ADAPTIVE_IMMUNE_SYSTEM |
| REACTOME_G1_PHASE |
| REACTOME_DNA_REPLICATION |
| KEGG_APOPTOSIS |
| KEGG_FOCAL_ADHESION |
| KEGG_TGF_BETA_SIGNALING_PATHWAY |
| module 366 |
| BIOCARTA_TGFB_PATHWAY |
| PID_WNT_NONCANONICAL_PATHWAY |
| REACTOME_ADAPTIVE_IMMUNE_SYSTEM |
| REACTOME_DNA_REPLICATION |
| REACTOME_G0_AND_EARLY_G1 |
| REACTOME_SIGNALING_BY_BMP |
| KEGG_APOPTOSIS |
| KEGG_FOCAL_ADHESION |
| module 367 |
| BIOCARTA_TGFB_PATHWAY |
| PID_WNT_NONCANONICAL_PATHWAY |
| REACTOME_ADAPTIVE_IMMUNE_SYSTEM |
| REACTOME_DNA_REPLICATION |
| REACTOME_G0_AND_EARLY_G1 |
| KEGG_APOPTOSIS |
| KEGG_FOCAL_ADHESION |
| KEGG_TGF_BETA_SIGNALING_PATHWAY |
| module 368 |
| BIOCARTA_TGFB_PATHWAY |
| REACTOME_ADAPTIVE_IMMUNE_SYSTEM |
| REACTOME_APOPTOSIS |
| REACTOME_G1_PHASE |
| REACTOME_DNA_REPLICATION |
| REACTOME_SIGNALING_BY_BMP |
| KEGG_FOCAL_ADHESION |
| KEGG_WNT_SIGNALING_PATHWAY |
| module 369 |
| BIOCARTA_TGFB_PATHWAY |
| REACTOME_ADAPTIVE_IMMUNE_SYSTEM |
| REACTOME_APOPTOSIS |
| REACTOME_G1_PHASE |
| REACTOME_DNA_REPLICATION |
| KEGG_FOCAL_ADHESION |
| KEGG_TGF_BETA_SIGNALING_PATHWAY |
| KEGG_WNT_SIGNALING_PATHWAY |
| module 370 |
| BIOCARTA_TGFB_PATHWAY |
| REACTOME_ADAPTIVE_IMMUNE_SYSTEM |
| REACTOME_APOPTOSIS |
| REACTOME_DNA_REPLICATION |
| REACTOME_G0_AND_EARLY_G1 |
| REACTOME_SIGNALING_BY_BMP |
| KEGG_FOCAL_ADHESION |
| KEGG_WNT_SIGNALING_PATHWAY |
| module 371 |
| BIOCARTA_TGFB_PATHWAY |
| REACTOME_ADAPTIVE_IMMUNE_SYSTEM |
| REACTOME_APOPTOSIS |
| REACTOME_DNA_REPLICATION |
| REACTOME_G0_AND_EARLY_G1 |
| KEGG_FOCAL_ADHESION |
| KEGG_TGF_BETA_SIGNALING_PATHWAY |
| KEGG_WNT_SIGNALING_PATHWAY |
| module 372 |
| BIOCARTA_TGFB_PATHWAY |
| REACTOME_ADAPTIVE_IMMUNE_SYSTEM |
| REACTOME_EXTRINSIC_PATHWAY_FOR_APOPTOSIS |
| REACTOME_G1_PHASE |
| REACTOME_DNA_REPLICATION |
| REACTOME_SIGNALING_BY_BMP |
| KEGG_FOCAL_ADHESION |
| KEGG_WNT_SIGNALING_PATHWAY |
| module 373 |
| BIOCARTA_TGFB_PATHWAY |
| REACTOME_ADAPTIVE_IMMUNE_SYSTEM |
| REACTOME_EXTRINSIC_PATHWAY_FOR_APOPTOSIS |
| REACTOME_G1_PHASE |
| REACTOME_DNA_REPLICATION |
| KEGG_FOCAL_ADHESION |
| KEGG_TGF_BETA_SIGNALING_PATHWAY |
| KEGG_WNT_SIGNALING_PATHWAY |
| module 374 |
| BIOCARTA_TGFB_PATHWAY |
| REACTOME_ADAPTIVE_IMMUNE_SYSTEM |
| REACTOME_EXTRINSIC_PATHWAY_FOR_APOPTOSIS |
| REACTOME_DNA_REPLICATION |
| REACTOME_G0_AND_EARLY_G1 |
| REACTOME_SIGNALING_BY_BMP |
| KEGG_FOCAL_ADHESION |
| KEGG_WNT_SIGNALING_PATHWAY |
| module 375 |
| BIOCARTA_TGFB_PATHWAY |
| REACTOME_ADAPTIVE_IMMUNE_SYSTEM |
| REACTOME_EXTRINSIC_PATHWAY_FOR_APOPTOSIS |
| REACTOME_DNA_REPLICATION |
| REACTOME_G0_AND_EARLY_G1 |
| KEGG_FOCAL_ADHESION |
| KEGG_TGF_BETA_SIGNALING_PATHWAY |
| KEGG_WNT_SIGNALING_PATHWAY |
| module 376 |
| BIOCARTA_TGFB_PATHWAY |
| REACTOME_ADAPTIVE_IMMUNE_SYSTEM |
| REACTOME_G1_PHASE |
| REACTOME_DNA_REPLICATION |
| REACTOME_SIGNALING_BY_BMP |
| KEGG_APOPTOSIS |
| KEGG_FOCAL_ADHESION |
| KEGG_WNT_SIGNALING_PATHWAY |
| module 377 |
| BIOCARTA_TGFB_PATHWAY |
| REACTOME_ADAPTIVE_IMMUNE_SYSTEM |
| REACTOME_G1_PHASE |
| REACTOME_DNA_REPLICATION |
| KEGG_APOPTOSIS |
| KEGG_FOCAL_ADHESION |
| KEGG_TGF_BETA_SIGNALING_PATHWAY |
| KEGG_WNT_SIGNALING_PATHWAY |
| module 378 |
| BIOCARTA_TGFB_PATHWAY |
| REACTOME_ADAPTIVE_IMMUNE_SYSTEM |
| REACTOME_DNA_REPLICATION |
| REACTOME_G0_AND_EARLY_G1 |
| REACTOME_SIGNALING_BY_BMP |
| KEGG_APOPTOSIS |
| KEGG_FOCAL_ADHESION |
| KEGG_WNT_SIGNALING_PATHWAY |
| module 379 |
| BIOCARTA_TGFB_PATHWAY |
| REACTOME_ADAPTIVE_IMMUNE_SYSTEM |
| REACTOME_DNA_REPLICATION |
| REACTOME_G0_AND_EARLY_G1 |
| KEGG_APOPTOSIS |
| KEGG_FOCAL_ADHESION |
| KEGG_TGF_BETA_SIGNALING_PATHWAY |
| KEGG_WNT_SIGNALING_PATHWAY |
| module 380 |
| BIOCARTA_TGFB_PATHWAY |
| PID_ALK1_PATHWAY |
| PID_WNT_NONCANONICAL_PATHWAY |
| REACTOME_ANTIGEN_PROCESSING_UBIQUITINATION_PROTEASOME_DEGRADATION |
| REACTOME_EXTRINSIC_PATHWAY_FOR_APOPTOSIS |
| REACTOME_MITOTIC_M_M_G1_PHASES |
| REACTOME_G0_AND_EARLY_G1 |
| KEGG_FOCAL_ADHESION |
| module 381 |
| BIOCARTA_TGFB_PATHWAY |
| PID_ALK1_PATHWAY |
| PID_WNT_NONCANONICAL_PATHWAY |
| REACTOME_ANTIGEN_PROCESSING_UBIQUITINATION_PROTEASOME_DEGRADATION |
| REACTOME_MITOTIC_M_M_G1_PHASES |
| REACTOME_G0_AND_EARLY_G1 |
| KEGG_APOPTOSIS |
| KEGG_FOCAL_ADHESION |
| module 382 |
| BIOCARTA_TGFB_PATHWAY |
| PID_ALK1_PATHWAY |
| PID_WNT_NONCANONICAL_PATHWAY |
| REACTOME_CLASS_I_MHC_MEDIATED_ANTIGEN_PROCESSING_PRESENTATION |
| REACTOME_EXTRINSIC_PATHWAY_FOR_APOPTOSIS |
| REACTOME_MITOTIC_M_M_G1_PHASES |
| REACTOME_G0_AND_EARLY_G1 |
| KEGG_FOCAL_ADHESION |
| module 383 |
| BIOCARTA_TGFB_PATHWAY |
| PID_ALK1_PATHWAY |
| PID_WNT_NONCANONICAL_PATHWAY |
| REACTOME_CLASS_I_MHC_MEDIATED_ANTIGEN_PROCESSING_PRESENTATION |
| REACTOME_MITOTIC_M_M_G1_PHASES |
| REACTOME_G0_AND_EARLY_G1 |
| KEGG_APOPTOSIS |
| KEGG_FOCAL_ADHESION |
| module 384 |
| BIOCARTA_TGFB_PATHWAY |
| PID_ALK1_PATHWAY |
| REACTOME_ANTIGEN_PROCESSING_UBIQUITINATION_PROTEASOME_DEGRADATION |
| REACTOME_EXTRINSIC_PATHWAY_FOR_APOPTOSIS |
| REACTOME_MITOTIC_M_M_G1_PHASES |
| REACTOME_G0_AND_EARLY_G1 |
| KEGG_FOCAL_ADHESION |
| KEGG_WNT_SIGNALING_PATHWAY |
| module 385 |
| BIOCARTA_TGFB_PATHWAY |
| PID_ALK1_PATHWAY |
| REACTOME_ANTIGEN_PROCESSING_UBIQUITINATION_PROTEASOME_DEGRADATION |
| REACTOME_MITOTIC_M_M_G1_PHASES |
| REACTOME_G0_AND_EARLY_G1 |
| KEGG_APOPTOSIS |
| KEGG_FOCAL_ADHESION |
| KEGG_WNT_SIGNALING_PATHWAY |
| module 386 |
| BIOCARTA_TGFB_PATHWAY |
| PID_ALK1_PATHWAY |
| REACTOME_CLASS_I_MHC_MEDIATED_ANTIGEN_PROCESSING_PRESENTATION |
| REACTOME_EXTRINSIC_PATHWAY_FOR_APOPTOSIS |
| REACTOME_MITOTIC_M_M_G1_PHASES |
| REACTOME_G0_AND_EARLY_G1 |
| KEGG_FOCAL_ADHESION |
| KEGG_WNT_SIGNALING_PATHWAY |
| module 387 |
| BIOCARTA_TGFB_PATHWAY |
| PID_ALK1_PATHWAY |
| REACTOME_CLASS_I_MHC_MEDIATED_ANTIGEN_PROCESSING_PRESENTATION |
| REACTOME_MITOTIC_M_M_G1_PHASES |
| REACTOME_G0_AND_EARLY_G1 |
| KEGG_APOPTOSIS |
| KEGG_FOCAL_ADHESION |
| KEGG_WNT_SIGNALING_PATHWAY |
| module 388 |
| BIOCARTA_TGFB_PATHWAY |
| PID_WNT_NONCANONICAL_PATHWAY |
| REACTOME_ANTIGEN_PROCESSING_UBIQUITINATION_PROTEASOME_DEGRADATION |
| REACTOME_EXTRINSIC_PATHWAY_FOR_APOPTOSIS |
| REACTOME_G1_PHASE |
| REACTOME_MITOTIC_M_M_G1_PHASES |
| REACTOME_SIGNALING_BY_BMP |
| KEGG_FOCAL_ADHESION |
| module 389 |
| BIOCARTA_TGFB_PATHWAY |
| PID_WNT_NONCANONICAL_PATHWAY |
| REACTOME_ANTIGEN_PROCESSING_UBIQUITINATION_PROTEASOME_DEGRADATION |
| REACTOME_EXTRINSIC_PATHWAY_FOR_APOPTOSIS |
| REACTOME_MITOTIC_M_M_G1_PHASES |
| REACTOME_G0_AND_EARLY_G1 |
| REACTOME_SIGNALING_BY_BMP |
| KEGG_FOCAL_ADHESION |
| module 390 |
| BIOCARTA_TGFB_PATHWAY |
| PID_WNT_NONCANONICAL_PATHWAY |
| REACTOME_ANTIGEN_PROCESSING_UBIQUITINATION_PROTEASOME_DEGRADATION |
| REACTOME_EXTRINSIC_PATHWAY_FOR_APOPTOSIS |
| REACTOME_MITOTIC_M_M_G1_PHASES |
| REACTOME_G0_AND_EARLY_G1 |
| KEGG_FOCAL_ADHESION |
| KEGG_TGF_BETA_SIGNALING_PATHWAY |
| module 391 |
| BIOCARTA_TGFB_PATHWAY |
| PID_WNT_NONCANONICAL_PATHWAY |
| REACTOME_ANTIGEN_PROCESSING_UBIQUITINATION_PROTEASOME_DEGRADATION |
| REACTOME_G1_PHASE |
| REACTOME_MITOTIC_M_M_G1_PHASES |
| REACTOME_SIGNALING_BY_BMP |
| KEGG_APOPTOSIS |
| KEGG_FOCAL_ADHESION |
| module 392 |
| BIOCARTA_TGFB_PATHWAY |
| PID_WNT_NONCANONICAL_PATHWAY |
| REACTOME_ANTIGEN_PROCESSING_UBIQUITINATION_PROTEASOME_DEGRADATION |
| REACTOME_MITOTIC_M_M_G1_PHASES |
| REACTOME_G0_AND_EARLY_G1 |
| REACTOME_SIGNALING_BY_BMP |
| KEGG_APOPTOSIS |
| KEGG_FOCAL_ADHESION |
| module 393 |
| BIOCARTA_TGFB_PATHWAY |
| PID_WNT_NONCANONICAL_PATHWAY |
| REACTOME_ANTIGEN_PROCESSING_UBIQUITINATION_PROTEASOME_DEGRADATION |
| REACTOME_MITOTIC_M_M_G1_PHASES |
| REACTOME_G0_AND_EARLY_G1 |
| KEGG_APOPTOSIS |
| KEGG_FOCAL_ADHESION |
| KEGG_TGF_BETA_SIGNALING_PATHWAY |
| module 394 |
| BIOCARTA_TGFB_PATHWAY |
| PID_WNT_NONCANONICAL_PATHWAY |
| REACTOME_CLASS_I_MHC_MEDIATED_ANTIGEN_PROCESSING_PRESENTATION |
| REACTOME_EXTRINSIC_PATHWAY_FOR_APOPTOSIS |
| REACTOME_G1_PHASE |
| REACTOME_MITOTIC_M_M_G1_PHASES |
| REACTOME_SIGNALING_BY_BMP |
| KEGG_FOCAL_ADHESION |
| module 395 |
| BIOCARTA_TGFB_PATHWAY |
| PID_WNT_NONCANONICAL_PATHWAY |
| REACTOME_CLASS_I_MHC_MEDIATED_ANTIGEN_PROCESSING_PRESENTATION |
| REACTOME_EXTRINSIC_PATHWAY_FOR_APOPTOSIS |
| REACTOME_MITOTIC_M_M_G1_PHASES |
| REACTOME_G0_AND_EARLY_G1 |
| REACTOME_SIGNALING_BY_BMP |
| KEGG_FOCAL_ADHESION |
| module 396 |
| BIOCARTA_TGFB_PATHWAY |
| PID_WNT_NONCANONICAL_PATHWAY |
| REACTOME_CLASS_I_MHC_MEDIATED_ANTIGEN_PROCESSING_PRESENTATION |
| REACTOME_EXTRINSIC_PATHWAY_FOR_APOPTOSIS |
| REACTOME_MITOTIC_M_M_G1_PHASES |
| REACTOME_G0_AND_EARLY_G1 |
| KEGG_FOCAL_ADHESION |
| KEGG_TGF_BETA_SIGNALING_PATHWAY |
| module 397 |
| BIOCARTA_TGFB_PATHWAY |
| PID_WNT_NONCANONICAL_PATHWAY |
| REACTOME_CLASS_I_MHC_MEDIATED_ANTIGEN_PROCESSING_PRESENTATION |
| REACTOME_G1_PHASE |
| REACTOME_MITOTIC_M_M_G1_PHASES |
| REACTOME_SIGNALING_BY_BMP |
| KEGG_APOPTOSIS |
| KEGG_FOCAL_ADHESION |
| module 398 |
| BIOCARTA_TGFB_PATHWAY |
| PID_WNT_NONCANONICAL_PATHWAY |
| REACTOME_CLASS_I_MHC_MEDIATED_ANTIGEN_PROCESSING_PRESENTATION |
| REACTOME_MITOTIC_M_M_G1_PHASES |
| REACTOME_G0_AND_EARLY_G1 |
| REACTOME_SIGNALING_BY_BMP |
| KEGG_APOPTOSIS |
| KEGG_FOCAL_ADHESION |
| module 399 |
| BIOCARTA_TGFB_PATHWAY |
| PID_WNT_NONCANONICAL_PATHWAY |
| REACTOME_CLASS_I_MHC_MEDIATED_ANTIGEN_PROCESSING_PRESENTATION |
| REACTOME_MITOTIC_M_M_G1_PHASES |
| REACTOME_G0_AND_EARLY_G1 |
| KEGG_APOPTOSIS |
| KEGG_FOCAL_ADHESION |
| KEGG_TGF_BETA_SIGNALING_PATHWAY |
| module 400 |
| BIOCARTA_TGFB_PATHWAY |
| REACTOME_ANTIGEN_PROCESSING_UBIQUITINATION_PROTEASOME_DEGRADATION |
| REACTOME_EXTRINSIC_PATHWAY_FOR_APOPTOSIS |
| REACTOME_G1_PHASE |
| REACTOME_MITOTIC_M_M_G1_PHASES |
| REACTOME_SIGNALING_BY_BMP |
| KEGG_FOCAL_ADHESION |
| KEGG_WNT_SIGNALING_PATHWAY |
| module 401 |
| BIOCARTA_TGFB_PATHWAY |
| REACTOME_ANTIGEN_PROCESSING_UBIQUITINATION_PROTEASOME_DEGRADATION |
| REACTOME_EXTRINSIC_PATHWAY_FOR_APOPTOSIS |
| REACTOME_MITOTIC_M_M_G1_PHASES |
| REACTOME_G0_AND_EARLY_G1 |
| REACTOME_SIGNALING_BY_BMP |
| KEGG_FOCAL_ADHESION |
| KEGG_WNT_SIGNALING_PATHWAY |
| module 402 |
| BIOCARTA_TGFB_PATHWAY |
| REACTOME_ANTIGEN_PROCESSING_UBIQUITINATION_PROTEASOME_DEGRADATION |
| REACTOME_EXTRINSIC_PATHWAY_FOR_APOPTOSIS |
| REACTOME_MITOTIC_M_M_G1_PHASES |
| REACTOME_G0_AND_EARLY_G1 |
| KEGG_FOCAL_ADHESION |
| KEGG_TGF_BETA_SIGNALING_PATHWAY |
| KEGG_WNT_SIGNALING_PATHWAY |
| module 403 |
| BIOCARTA_TGFB_PATHWAY |
| REACTOME_ANTIGEN_PROCESSING_UBIQUITINATION_PROTEASOME_DEGRADATION |
| REACTOME_G1_PHASE |
| REACTOME_MITOTIC_M_M_G1_PHASES |
| REACTOME_SIGNALING_BY_BMP |
| KEGG_APOPTOSIS |
| KEGG_FOCAL_ADHESION |
| KEGG_WNT_SIGNALING_PATHWAY |
| module 404 |
| BIOCARTA_TGFB_PATHWAY |
| REACTOME_ANTIGEN_PROCESSING_UBIQUITINATION_PROTEASOME_DEGRADATION |
| REACTOME_MITOTIC_M_M_G1_PHASES |
| REACTOME_G0_AND_EARLY_G1 |
| REACTOME_SIGNALING_BY_BMP |
| KEGG_APOPTOSIS |
| KEGG_FOCAL_ADHESION |
| KEGG_WNT_SIGNALING_PATHWAY |
| module 405 |
| BIOCARTA_TGFB_PATHWAY |
| REACTOME_ANTIGEN_PROCESSING_UBIQUITINATION_PROTEASOME_DEGRADATION |
| REACTOME_MITOTIC_M_M_G1_PHASES |
| REACTOME_G0_AND_EARLY_G1 |
| KEGG_APOPTOSIS |
| KEGG_FOCAL_ADHESION |
| KEGG_TGF_BETA_SIGNALING_PATHWAY |
| KEGG_WNT_SIGNALING_PATHWAY |
| module 406 |
| BIOCARTA_TGFB_PATHWAY |
| REACTOME_CLASS_I_MHC_MEDIATED_ANTIGEN_PROCESSING_PRESENTATION |
| REACTOME_EXTRINSIC_PATHWAY_FOR_APOPTOSIS |
| REACTOME_G1_PHASE |
| REACTOME_MITOTIC_M_M_G1_PHASES |
| REACTOME_SIGNALING_BY_BMP |
| KEGG_FOCAL_ADHESION |
| KEGG_WNT_SIGNALING_PATHWAY |
| module 407 |
| BIOCARTA_TGFB_PATHWAY |
| REACTOME_CLASS_I_MHC_MEDIATED_ANTIGEN_PROCESSING_PRESENTATION |
| REACTOME_EXTRINSIC_PATHWAY_FOR_APOPTOSIS |
| REACTOME_MITOTIC_M_M_G1_PHASES |
| REACTOME_G0_AND_EARLY_G1 |
| REACTOME_SIGNALING_BY_BMP |
| KEGG_FOCAL_ADHESION |
| KEGG_WNT_SIGNALING_PATHWAY |
| module 408 |
| BIOCARTA_TGFB_PATHWAY |
| REACTOME_CLASS_I_MHC_MEDIATED_ANTIGEN_PROCESSING_PRESENTATION |
| REACTOME_EXTRINSIC_PATHWAY_FOR_APOPTOSIS |
| REACTOME_MITOTIC_M_M_G1_PHASES |
| REACTOME_G0_AND_EARLY_G1 |
| KEGG_FOCAL_ADHESION |
| KEGG_TGF_BETA_SIGNALING_PATHWAY |
| KEGG_WNT_SIGNALING_PATHWAY |
| module 409 |
| BIOCARTA_TGFB_PATHWAY |
| REACTOME_CLASS_I_MHC_MEDIATED_ANTIGEN_PROCESSING_PRESENTATION |
| REACTOME_G1_PHASE |
| REACTOME_MITOTIC_M_M_G1_PHASES |
| REACTOME_SIGNALING_BY_BMP |
| KEGG_APOPTOSIS |
| KEGG_FOCAL_ADHESION |
| KEGG_WNT_SIGNALING_PATHWAY |
| module 410 |
| BIOCARTA_TGFB_PATHWAY |
| REACTOME_CLASS_I_MHC_MEDIATED_ANTIGEN_PROCESSING_PRESENTATION |
| REACTOME_MITOTIC_M_M_G1_PHASES |
| REACTOME_G0_AND_EARLY_G1 |
| REACTOME_SIGNALING_BY_BMP |
| KEGG_APOPTOSIS |
| KEGG_FOCAL_ADHESION |
| KEGG_WNT_SIGNALING_PATHWAY |
| module 411 |
| BIOCARTA_TGFB_PATHWAY |
| REACTOME_CLASS_I_MHC_MEDIATED_ANTIGEN_PROCESSING_PRESENTATION |
| REACTOME_MITOTIC_M_M_G1_PHASES |
| REACTOME_G0_AND_EARLY_G1 |
| KEGG_APOPTOSIS |
| KEGG_FOCAL_ADHESION |
| KEGG_TGF_BETA_SIGNALING_PATHWAY |
| KEGG_WNT_SIGNALING_PATHWAY |
| module 412 |
| BIOCARTA_TGFB_PATHWAY |
| PID_ALK1_PATHWAY |
| PID_WNT_NONCANONICAL_PATHWAY |
| REACTOME_ANTIGEN_PROCESSING_UBIQUITINATION_PROTEASOME_DEGRADATION |
| REACTOME_APOPTOSIS |
| REACTOME_DNA_REPLICATION |
| REACTOME_G0_AND_EARLY_G1 |
| KEGG_FOCAL_ADHESION |
| module 413 |
| BIOCARTA_TGFB_PATHWAY |
| PID_ALK1_PATHWAY |
| PID_WNT_NONCANONICAL_PATHWAY |
| REACTOME_ANTIGEN_PROCESSING_UBIQUITINATION_PROTEASOME_DEGRADATION |
| REACTOME_EXTRINSIC_PATHWAY_FOR_APOPTOSIS |
| REACTOME_DNA_REPLICATION |
| REACTOME_G0_AND_EARLY_G1 |
| KEGG_FOCAL_ADHESION |
| module 414 |
| BIOCARTA_TGFB_PATHWAY |
| PID_ALK1_PATHWAY |
| PID_WNT_NONCANONICAL_PATHWAY |
| REACTOME_ANTIGEN_PROCESSING_UBIQUITINATION_PROTEASOME_DEGRADATION |
| REACTOME_DNA_REPLICATION |
| REACTOME_G0_AND_EARLY_G1 |
| KEGG_APOPTOSIS |
| KEGG_FOCAL_ADHESION |
| module 415 |
| BIOCARTA_TGFB_PATHWAY |
| PID_ALK1_PATHWAY |
| PID_WNT_NONCANONICAL_PATHWAY |
| REACTOME_APOPTOSIS |
| REACTOME_CLASS_I_MHC_MEDIATED_ANTIGEN_PROCESSING_PRESENTATION |
| REACTOME_DNA_REPLICATION |
| REACTOME_G0_AND_EARLY_G1 |
| KEGG_FOCAL_ADHESION |
| module 416 |
| BIOCARTA_TGFB_PATHWAY |
| PID_ALK1_PATHWAY |
| PID_WNT_NONCANONICAL_PATHWAY |
| REACTOME_CLASS_I_MHC_MEDIATED_ANTIGEN_PROCESSING_PRESENTATION |
| REACTOME_EXTRINSIC_PATHWAY_FOR_APOPTOSIS |
| REACTOME_DNA_REPLICATION |
| REACTOME_G0_AND_EARLY_G1 |
| KEGG_FOCAL_ADHESION |
| module 417 |
| BIOCARTA_TGFB_PATHWAY |
| PID_ALK1_PATHWAY |
| PID_WNT_NONCANONICAL_PATHWAY |
| REACTOME_CLASS_I_MHC_MEDIATED_ANTIGEN_PROCESSING_PRESENTATION |
| REACTOME_DNA_REPLICATION |
| REACTOME_G0_AND_EARLY_G1 |
| KEGG_APOPTOSIS |
| KEGG_FOCAL_ADHESION |
| module 418 |
| BIOCARTA_TGFB_PATHWAY |
| PID_ALK1_PATHWAY |
| REACTOME_ANTIGEN_PROCESSING_UBIQUITINATION_PROTEASOME_DEGRADATION |
| REACTOME_APOPTOSIS |
| REACTOME_DNA_REPLICATION |
| REACTOME_G0_AND_EARLY_G1 |
| KEGG_FOCAL_ADHESION |
| KEGG_WNT_SIGNALING_PATHWAY |
| module 419 |
| BIOCARTA_TGFB_PATHWAY |
| PID_ALK1_PATHWAY |
| REACTOME_ANTIGEN_PROCESSING_UBIQUITINATION_PROTEASOME_DEGRADATION |
| REACTOME_EXTRINSIC_PATHWAY_FOR_APOPTOSIS |
| REACTOME_DNA_REPLICATION |
| REACTOME_G0_AND_EARLY_G1 |
| KEGG_FOCAL_ADHESION |
| KEGG_WNT_SIGNALING_PATHWAY |
| module 420 |
| BIOCARTA_TGFB_PATHWAY |
| PID_ALK1_PATHWAY |
| REACTOME_ANTIGEN_PROCESSING_UBIQUITINATION_PROTEASOME_DEGRADATION |
| REACTOME_DNA_REPLICATION |
| REACTOME_G0_AND_EARLY_G1 |
| KEGG_APOPTOSIS |
| KEGG_FOCAL_ADHESION |
| KEGG_WNT_SIGNALING_PATHWAY |
| module 421 |
| BIOCARTA_TGFB_PATHWAY |
| PID_ALK1_PATHWAY |
| REACTOME_APOPTOSIS |
| REACTOME_CLASS_I_MHC_MEDIATED_ANTIGEN_PROCESSING_PRESENTATION |
| REACTOME_DNA_REPLICATION |
| REACTOME_G0_AND_EARLY_G1 |
| KEGG_FOCAL_ADHESION |
| KEGG_WNT_SIGNALING_PATHWAY |
| module 422 |
| BIOCARTA_TGFB_PATHWAY |
| PID_ALK1_PATHWAY |
| REACTOME_CLASS_I_MHC_MEDIATED_ANTIGEN_PROCESSING_PRESENTATION |
| REACTOME_EXTRINSIC_PATHWAY_FOR_APOPTOSIS |
| REACTOME_DNA_REPLICATION |
| REACTOME_G0_AND_EARLY_G1 |
| KEGG_FOCAL_ADHESION |
| KEGG_WNT_SIGNALING_PATHWAY |
| module 423 |
| BIOCARTA_TGFB_PATHWAY |
| PID_ALK1_PATHWAY |
| REACTOME_CLASS_I_MHC_MEDIATED_ANTIGEN_PROCESSING_PRESENTATION |
| REACTOME_DNA_REPLICATION |
| REACTOME_G0_AND_EARLY_G1 |
| KEGG_APOPTOSIS |
| KEGG_FOCAL_ADHESION |
| KEGG_WNT_SIGNALING_PATHWAY |
| module 424 |
| BIOCARTA_TGFB_PATHWAY |
| PID_WNT_NONCANONICAL_PATHWAY |
| REACTOME_ANTIGEN_PROCESSING_UBIQUITINATION_PROTEASOME_DEGRADATION |
| REACTOME_APOPTOSIS |
| REACTOME_DNA_REPLICATION |
| REACTOME_G0_AND_EARLY_G1 |
| REACTOME_SIGNALING_BY_BMP |
| KEGG_FOCAL_ADHESION |
| module 425 |
| BIOCARTA_TGFB_PATHWAY |
| PID_WNT_NONCANONICAL_PATHWAY |
| REACTOME_ANTIGEN_PROCESSING_UBIQUITINATION_PROTEASOME_DEGRADATION |
| REACTOME_APOPTOSIS |
| REACTOME_DNA_REPLICATION |
| REACTOME_G0_AND_EARLY_G1 |
| KEGG_FOCAL_ADHESION |
| KEGG_TGF_BETA_SIGNALING_PATHWAY |
| module 426 |
| BIOCARTA_TGFB_PATHWAY |
| PID_WNT_NONCANONICAL_PATHWAY |
| REACTOME_ANTIGEN_PROCESSING_UBIQUITINATION_PROTEASOME_DEGRADATION |
| REACTOME_EXTRINSIC_PATHWAY_FOR_APOPTOSIS |
| REACTOME_DNA_REPLICATION |
| REACTOME_G0_AND_EARLY_G1 |
| REACTOME_SIGNALING_BY_BMP |
| KEGG_FOCAL_ADHESION |
| module 427 |
| BIOCARTA_TGFB_PATHWAY |
| PID_WNT_NONCANONICAL_PATHWAY |
| REACTOME_ANTIGEN_PROCESSING_UBIQUITINATION_PROTEASOME_DEGRADATION |
| REACTOME_EXTRINSIC_PATHWAY_FOR_APOPTOSIS |
| REACTOME_DNA_REPLICATION |
| REACTOME_G0_AND_EARLY_G1 |
| KEGG_FOCAL_ADHESION |
| KEGG_TGF_BETA_SIGNALING_PATHWAY |
| module 428 |
| BIOCARTA_TGFB_PATHWAY |
| PID_WNT_NONCANONICAL_PATHWAY |
| REACTOME_ANTIGEN_PROCESSING_UBIQUITINATION_PROTEASOME_DEGRADATION |
| REACTOME_DNA_REPLICATION |
| REACTOME_G0_AND_EARLY_G1 |
| REACTOME_SIGNALING_BY_BMP |
| KEGG_APOPTOSIS |
| KEGG_FOCAL_ADHESION |
| module 429 |
| BIOCARTA_TGFB_PATHWAY |
| PID_WNT_NONCANONICAL_PATHWAY |
| REACTOME_ANTIGEN_PROCESSING_UBIQUITINATION_PROTEASOME_DEGRADATION |
| REACTOME_DNA_REPLICATION |
| REACTOME_G0_AND_EARLY_G1 |
| KEGG_APOPTOSIS |
| KEGG_FOCAL_ADHESION |
| KEGG_TGF_BETA_SIGNALING_PATHWAY |
| module 430 |
| BIOCARTA_TGFB_PATHWAY |
| PID_WNT_NONCANONICAL_PATHWAY |
| REACTOME_APOPTOSIS |
| REACTOME_CLASS_I_MHC_MEDIATED_ANTIGEN_PROCESSING_PRESENTATION |
| REACTOME_DNA_REPLICATION |
| REACTOME_G0_AND_EARLY_G1 |
| REACTOME_SIGNALING_BY_BMP |
| KEGG_FOCAL_ADHESION |
| module 431 |
| BIOCARTA_TGFB_PATHWAY |
| PID_WNT_NONCANONICAL_PATHWAY |
| REACTOME_APOPTOSIS |
| REACTOME_CLASS_I_MHC_MEDIATED_ANTIGEN_PROCESSING_PRESENTATION |
| REACTOME_DNA_REPLICATION |
| REACTOME_G0_AND_EARLY_G1 |
| KEGG_FOCAL_ADHESION |
| KEGG_TGF_BETA_SIGNALING_PATHWAY |
| module 432 |
| BIOCARTA_TGFB_PATHWAY |
| PID_WNT_NONCANONICAL_PATHWAY |
| REACTOME_CLASS_I_MHC_MEDIATED_ANTIGEN_PROCESSING_PRESENTATION |
| REACTOME_EXTRINSIC_PATHWAY_FOR_APOPTOSIS |
| REACTOME_DNA_REPLICATION |
| REACTOME_G0_AND_EARLY_G1 |
| REACTOME_SIGNALING_BY_BMP |
| KEGG_FOCAL_ADHESION |
| module 433 |
| BIOCARTA_TGFB_PATHWAY |
| PID_WNT_NONCANONICAL_PATHWAY |
| REACTOME_CLASS_I_MHC_MEDIATED_ANTIGEN_PROCESSING_PRESENTATION |
| REACTOME_EXTRINSIC_PATHWAY_FOR_APOPTOSIS |
| REACTOME_DNA_REPLICATION |
| REACTOME_G0_AND_EARLY_G1 |
| KEGG_FOCAL_ADHESION |
| KEGG_TGF_BETA_SIGNALING_PATHWAY |
| module 434 |
| BIOCARTA_TGFB_PATHWAY |
| PID_WNT_NONCANONICAL_PATHWAY |
| REACTOME_CLASS_I_MHC_MEDIATED_ANTIGEN_PROCESSING_PRESENTATION |
| REACTOME_DNA_REPLICATION |
| REACTOME_G0_AND_EARLY_G1 |
| REACTOME_SIGNALING_BY_BMP |
| KEGG_APOPTOSIS |
| KEGG_FOCAL_ADHESION |
| module 435 |
| BIOCARTA_TGFB_PATHWAY |
| PID_WNT_NONCANONICAL_PATHWAY |
| REACTOME_CLASS_I_MHC_MEDIATED_ANTIGEN_PROCESSING_PRESENTATION |
| REACTOME_DNA_REPLICATION |
| REACTOME_G0_AND_EARLY_G1 |
| KEGG_APOPTOSIS |
| KEGG_FOCAL_ADHESION |
| KEGG_TGF_BETA_SIGNALING_PATHWAY |
| module 436 |
| BIOCARTA_TGFB_PATHWAY |
| REACTOME_ANTIGEN_PROCESSING_UBIQUITINATION_PROTEASOME_DEGRADATION |
| REACTOME_APOPTOSIS |
| REACTOME_DNA_REPLICATION |
| REACTOME_G0_AND_EARLY_G1 |
| REACTOME_SIGNALING_BY_BMP |
| KEGG_FOCAL_ADHESION |
| KEGG_WNT_SIGNALING_PATHWAY |
| module 437 |
| BIOCARTA_TGFB_PATHWAY |
| REACTOME_ANTIGEN_PROCESSING_UBIQUITINATION_PROTEASOME_DEGRADATION |
| REACTOME_APOPTOSIS |
| REACTOME_DNA_REPLICATION |
| REACTOME_G0_AND_EARLY_G1 |
| KEGG_FOCAL_ADHESION |
| KEGG_TGF_BETA_SIGNALING_PATHWAY |
| KEGG_WNT_SIGNALING_PATHWAY |
| module 438 |
| BIOCARTA_TGFB_PATHWAY |
| REACTOME_ANTIGEN_PROCESSING_UBIQUITINATION_PROTEASOME_DEGRADATION |
| REACTOME_EXTRINSIC_PATHWAY_FOR_APOPTOSIS |
| REACTOME_DNA_REPLICATION |
| REACTOME_G0_AND_EARLY_G1 |
| REACTOME_SIGNALING_BY_BMP |
| KEGG_FOCAL_ADHESION |
| KEGG_WNT_SIGNALING_PATHWAY |
| module 439 |
| BIOCARTA_TGFB_PATHWAY |
| REACTOME_ANTIGEN_PROCESSING_UBIQUITINATION_PROTEASOME_DEGRADATION |
| REACTOME_EXTRINSIC_PATHWAY_FOR_APOPTOSIS |
| REACTOME_DNA_REPLICATION |
| REACTOME_G0_AND_EARLY_G1 |
| KEGG_FOCAL_ADHESION |
| KEGG_TGF_BETA_SIGNALING_PATHWAY |
| KEGG_WNT_SIGNALING_PATHWAY |
| module 440 |
| BIOCARTA_TGFB_PATHWAY |
| REACTOME_ANTIGEN_PROCESSING_UBIQUITINATION_PROTEASOME_DEGRADATION |
| REACTOME_DNA_REPLICATION |
| REACTOME_G0_AND_EARLY_G1 |
| REACTOME_SIGNALING_BY_BMP |
| KEGG_APOPTOSIS |
| KEGG_FOCAL_ADHESION |
| KEGG_WNT_SIGNALING_PATHWAY |
| module 441 |
| BIOCARTA_TGFB_PATHWAY |
| REACTOME_ANTIGEN_PROCESSING_UBIQUITINATION_PROTEASOME_DEGRADATION |
| REACTOME_DNA_REPLICATION |
| REACTOME_G0_AND_EARLY_G1 |
| KEGG_APOPTOSIS |
| KEGG_FOCAL_ADHESION |
| KEGG_TGF_BETA_SIGNALING_PATHWAY |
| KEGG_WNT_SIGNALING_PATHWAY |
| module 442 |
| BIOCARTA_TGFB_PATHWAY |
| REACTOME_APOPTOSIS |
| REACTOME_CLASS_I_MHC_MEDIATED_ANTIGEN_PROCESSING_PRESENTATION |
| REACTOME_DNA_REPLICATION |
| REACTOME_G0_AND_EARLY_G1 |
| REACTOME_SIGNALING_BY_BMP |
| KEGG_FOCAL_ADHESION |
| KEGG_WNT_SIGNALING_PATHWAY |
| module 443 |
| BIOCARTA_TGFB_PATHWAY |
| REACTOME_APOPTOSIS |
| REACTOME_CLASS_I_MHC_MEDIATED_ANTIGEN_PROCESSING_PRESENTATION |
| REACTOME_DNA_REPLICATION |
| REACTOME_G0_AND_EARLY_G1 |
| KEGG_FOCAL_ADHESION |
| KEGG_TGF_BETA_SIGNALING_PATHWAY |
| KEGG_WNT_SIGNALING_PATHWAY |
| module 444 |
| BIOCARTA_TGFB_PATHWAY |
| REACTOME_CLASS_I_MHC_MEDIATED_ANTIGEN_PROCESSING_PRESENTATION |
| REACTOME_EXTRINSIC_PATHWAY_FOR_APOPTOSIS |
| REACTOME_DNA_REPLICATION |
| REACTOME_G0_AND_EARLY_G1 |
| REACTOME_SIGNALING_BY_BMP |
| KEGG_FOCAL_ADHESION |
| KEGG_WNT_SIGNALING_PATHWAY |
| module 445 |
| BIOCARTA_TGFB_PATHWAY |
| REACTOME_CLASS_I_MHC_MEDIATED_ANTIGEN_PROCESSING_PRESENTATION |
| REACTOME_EXTRINSIC_PATHWAY_FOR_APOPTOSIS |
| REACTOME_DNA_REPLICATION |
| REACTOME_G0_AND_EARLY_G1 |
| KEGG_FOCAL_ADHESION |
| KEGG_TGF_BETA_SIGNALING_PATHWAY |
| KEGG_WNT_SIGNALING_PATHWAY |
| module 446 |
| BIOCARTA_TGFB_PATHWAY |
| REACTOME_CLASS_I_MHC_MEDIATED_ANTIGEN_PROCESSING_PRESENTATION |
| REACTOME_DNA_REPLICATION |
| REACTOME_G0_AND_EARLY_G1 |
| REACTOME_SIGNALING_BY_BMP |
| KEGG_APOPTOSIS |
| KEGG_FOCAL_ADHESION |
| KEGG_WNT_SIGNALING_PATHWAY |
| module 447 |
| BIOCARTA_TGFB_PATHWAY |
| REACTOME_CLASS_I_MHC_MEDIATED_ANTIGEN_PROCESSING_PRESENTATION |
| REACTOME_DNA_REPLICATION |
| REACTOME_G0_AND_EARLY_G1 |
| KEGG_APOPTOSIS |
| KEGG_FOCAL_ADHESION |
| KEGG_TGF_BETA_SIGNALING_PATHWAY |
| KEGG_WNT_SIGNALING_PATHWAY |
| module 448 |
| BIOCARTA_TGFB_PATHWAY |
| PID_ALK1_PATHWAY |
| PID_WNT_NONCANONICAL_PATHWAY |
| REACTOME_ANTIGEN_PROCESSING_UBIQUITINATION_PROTEASOME_DEGRADATION |
| REACTOME_APOPTOSIS |
| REACTOME_G1_PHASE |
| REACTOME_DNA_REPLICATION |
| KEGG_FOCAL_ADHESION |
| module 449 |
| BIOCARTA_TGFB_PATHWAY |
| PID_ALK1_PATHWAY |
| PID_WNT_NONCANONICAL_PATHWAY |
| REACTOME_ANTIGEN_PROCESSING_UBIQUITINATION_PROTEASOME_DEGRADATION |
| REACTOME_EXTRINSIC_PATHWAY_FOR_APOPTOSIS |
| REACTOME_G1_PHASE |
| REACTOME_DNA_REPLICATION |
| KEGG_FOCAL_ADHESION |
| module 450 |
| BIOCARTA_TGFB_PATHWAY |
| PID_ALK1_PATHWAY |
| PID_WNT_NONCANONICAL_PATHWAY |
| REACTOME_ANTIGEN_PROCESSING_UBIQUITINATION_PROTEASOME_DEGRADATION |
| REACTOME_G1_PHASE |
| REACTOME_DNA_REPLICATION |
| KEGG_APOPTOSIS |
| KEGG_FOCAL_ADHESION |
| module 451 |
| BIOCARTA_TGFB_PATHWAY |
| PID_ALK1_PATHWAY |
| PID_WNT_NONCANONICAL_PATHWAY |
| REACTOME_APOPTOSIS |
| REACTOME_CLASS_I_MHC_MEDIATED_ANTIGEN_PROCESSING_PRESENTATION |
| REACTOME_G1_PHASE |
| REACTOME_DNA_REPLICATION |
| KEGG_FOCAL_ADHESION |
| module 452 |
| BIOCARTA_TGFB_PATHWAY |
| PID_ALK1_PATHWAY |
| PID_WNT_NONCANONICAL_PATHWAY |
| REACTOME_CLASS_I_MHC_MEDIATED_ANTIGEN_PROCESSING_PRESENTATION |
| REACTOME_EXTRINSIC_PATHWAY_FOR_APOPTOSIS |
| REACTOME_G1_PHASE |
| REACTOME_DNA_REPLICATION |
| KEGG_FOCAL_ADHESION |
| module 453 |
| BIOCARTA_TGFB_PATHWAY |
| PID_ALK1_PATHWAY |
| PID_WNT_NONCANONICAL_PATHWAY |
| REACTOME_CLASS_I_MHC_MEDIATED_ANTIGEN_PROCESSING_PRESENTATION |
| REACTOME_G1_PHASE |
| REACTOME_DNA_REPLICATION |
| KEGG_APOPTOSIS |
| KEGG_FOCAL_ADHESION |
| module 454 |
| BIOCARTA_TGFB_PATHWAY |
| PID_ALK1_PATHWAY |
| REACTOME_ANTIGEN_PROCESSING_UBIQUITINATION_PROTEASOME_DEGRADATION |
| REACTOME_APOPTOSIS |
| REACTOME_G1_PHASE |
| REACTOME_DNA_REPLICATION |
| KEGG_FOCAL_ADHESION |
| KEGG_WNT_SIGNALING_PATHWAY |
| module 455 |
| BIOCARTA_TGFB_PATHWAY |
| PID_ALK1_PATHWAY |
| REACTOME_ANTIGEN_PROCESSING_UBIQUITINATION_PROTEASOME_DEGRADATION |
| REACTOME_EXTRINSIC_PATHWAY_FOR_APOPTOSIS |
| REACTOME_G1_PHASE |
| REACTOME_DNA_REPLICATION |
| KEGG_FOCAL_ADHESION |
| KEGG_WNT_SIGNALING_PATHWAY |
| module 456 |
| BIOCARTA_TGFB_PATHWAY |
| PID_ALK1_PATHWAY |
| REACTOME_ANTIGEN_PROCESSING_UBIQUITINATION_PROTEASOME_DEGRADATION |
| REACTOME_G1_PHASE |
| REACTOME_DNA_REPLICATION |
| KEGG_APOPTOSIS |
| KEGG_FOCAL_ADHESION |
| KEGG_WNT_SIGNALING_PATHWAY |
| module 457 |
| BIOCARTA_TGFB_PATHWAY |
| PID_ALK1_PATHWAY |
| REACTOME_APOPTOSIS |
| REACTOME_CLASS_I_MHC_MEDIATED_ANTIGEN_PROCESSING_PRESENTATION |
| REACTOME_G1_PHASE |
| REACTOME_DNA_REPLICATION |
| KEGG_FOCAL_ADHESION |
| KEGG_WNT_SIGNALING_PATHWAY |
| module 458 |
| BIOCARTA_TGFB_PATHWAY |
| PID_ALK1_PATHWAY |
| REACTOME_CLASS_I_MHC_MEDIATED_ANTIGEN_PROCESSING_PRESENTATION |
| REACTOME_EXTRINSIC_PATHWAY_FOR_APOPTOSIS |
| REACTOME_G1_PHASE |
| REACTOME_DNA_REPLICATION |
| KEGG_FOCAL_ADHESION |
| KEGG_WNT_SIGNALING_PATHWAY |
| module 459 |
| BIOCARTA_TGFB_PATHWAY |
| PID_ALK1_PATHWAY |
| REACTOME_CLASS_I_MHC_MEDIATED_ANTIGEN_PROCESSING_PRESENTATION |
| REACTOME_G1_PHASE |
| REACTOME_DNA_REPLICATION |
| KEGG_APOPTOSIS |
| KEGG_FOCAL_ADHESION |
| KEGG_WNT_SIGNALING_PATHWAY |
| module 460 |
| BIOCARTA_TGFB_PATHWAY |
| PID_WNT_NONCANONICAL_PATHWAY |
| REACTOME_ANTIGEN_PROCESSING_UBIQUITINATION_PROTEASOME_DEGRADATION |
| REACTOME_APOPTOSIS |
| REACTOME_G1_PHASE |
| REACTOME_DNA_REPLICATION |
| REACTOME_SIGNALING_BY_BMP |
| KEGG_FOCAL_ADHESION |
| module 461 |
| BIOCARTA_TGFB_PATHWAY |
| PID_WNT_NONCANONICAL_PATHWAY |
| REACTOME_ANTIGEN_PROCESSING_UBIQUITINATION_PROTEASOME_DEGRADATION |
| REACTOME_APOPTOSIS |
| REACTOME_G1_PHASE |
| REACTOME_DNA_REPLICATION |
| KEGG_FOCAL_ADHESION |
| KEGG_TGF_BETA_SIGNALING_PATHWAY |
| module 462 |
| BIOCARTA_TGFB_PATHWAY |
| PID_WNT_NONCANONICAL_PATHWAY |
| REACTOME_ANTIGEN_PROCESSING_UBIQUITINATION_PROTEASOME_DEGRADATION |
| REACTOME_EXTRINSIC_PATHWAY_FOR_APOPTOSIS |
| REACTOME_G1_PHASE |
| REACTOME_DNA_REPLICATION |
| REACTOME_SIGNALING_BY_BMP |
| KEGG_FOCAL_ADHESION |
| module 463 |
| BIOCARTA_TGFB_PATHWAY |
| PID_WNT_NONCANONICAL_PATHWAY |
| REACTOME_ANTIGEN_PROCESSING_UBIQUITINATION_PROTEASOME_DEGRADATION |
| REACTOME_EXTRINSIC_PATHWAY_FOR_APOPTOSIS |
| REACTOME_G1_PHASE |
| REACTOME_DNA_REPLICATION |
| KEGG_FOCAL_ADHESION |
| KEGG_TGF_BETA_SIGNALING_PATHWAY |
| module 464 |
| BIOCARTA_TGFB_PATHWAY |
| PID_WNT_NONCANONICAL_PATHWAY |
| REACTOME_ANTIGEN_PROCESSING_UBIQUITINATION_PROTEASOME_DEGRADATION |
| REACTOME_G1_PHASE |
| REACTOME_DNA_REPLICATION |
| REACTOME_SIGNALING_BY_BMP |
| KEGG_APOPTOSIS |
| KEGG_FOCAL_ADHESION |
| module 465 |
| BIOCARTA_TGFB_PATHWAY |
| PID_WNT_NONCANONICAL_PATHWAY |
| REACTOME_ANTIGEN_PROCESSING_UBIQUITINATION_PROTEASOME_DEGRADATION |
| REACTOME_G1_PHASE |
| REACTOME_DNA_REPLICATION |
| KEGG_APOPTOSIS |
| KEGG_FOCAL_ADHESION |
| KEGG_TGF_BETA_SIGNALING_PATHWAY |
| module 466 |
| BIOCARTA_TGFB_PATHWAY |
| PID_WNT_NONCANONICAL_PATHWAY |
| REACTOME_APOPTOSIS |
| REACTOME_CLASS_I_MHC_MEDIATED_ANTIGEN_PROCESSING_PRESENTATION |
| REACTOME_G1_PHASE |
| REACTOME_DNA_REPLICATION |
| REACTOME_SIGNALING_BY_BMP |
| KEGG_FOCAL_ADHESION |
| module 467 |
| BIOCARTA_TGFB_PATHWAY |
| PID_WNT_NONCANONICAL_PATHWAY |
| REACTOME_APOPTOSIS |
| REACTOME_CLASS_I_MHC_MEDIATED_ANTIGEN_PROCESSING_PRESENTATION |
| REACTOME_G1_PHASE |
| REACTOME_DNA_REPLICATION |
| KEGG_FOCAL_ADHESION |
| KEGG_TGF_BETA_SIGNALING_PATHWAY |
| module 468 |
| BIOCARTA_TGFB_PATHWAY |
| PID_WNT_NONCANONICAL_PATHWAY |
| REACTOME_CLASS_I_MHC_MEDIATED_ANTIGEN_PROCESSING_PRESENTATION |
| REACTOME_EXTRINSIC_PATHWAY_FOR_APOPTOSIS |
| REACTOME_G1_PHASE |
| REACTOME_DNA_REPLICATION |
| REACTOME_SIGNALING_BY_BMP |
| KEGG_FOCAL_ADHESION |
| module 469 |
| BIOCARTA_TGFB_PATHWAY |
| PID_WNT_NONCANONICAL_PATHWAY |
| REACTOME_CLASS_I_MHC_MEDIATED_ANTIGEN_PROCESSING_PRESENTATION |
| REACTOME_EXTRINSIC_PATHWAY_FOR_APOPTOSIS |
| REACTOME_G1_PHASE |
| REACTOME_DNA_REPLICATION |
| KEGG_FOCAL_ADHESION |
| KEGG_TGF_BETA_SIGNALING_PATHWAY |
| module 470 |
| BIOCARTA_TGFB_PATHWAY |
| PID_WNT_NONCANONICAL_PATHWAY |
| REACTOME_CLASS_I_MHC_MEDIATED_ANTIGEN_PROCESSING_PRESENTATION |
| REACTOME_G1_PHASE |
| REACTOME_DNA_REPLICATION |
| REACTOME_SIGNALING_BY_BMP |
| KEGG_APOPTOSIS |
| KEGG_FOCAL_ADHESION |
| module 471 |
| BIOCARTA_TGFB_PATHWAY |
| PID_WNT_NONCANONICAL_PATHWAY |
| REACTOME_CLASS_I_MHC_MEDIATED_ANTIGEN_PROCESSING_PRESENTATION |
| REACTOME_G1_PHASE |
| REACTOME_DNA_REPLICATION |
| KEGG_APOPTOSIS |
| KEGG_FOCAL_ADHESION |
| KEGG_TGF_BETA_SIGNALING_PATHWAY |
| module 472 |
| BIOCARTA_TGFB_PATHWAY |
| REACTOME_ANTIGEN_PROCESSING_UBIQUITINATION_PROTEASOME_DEGRADATION |
| REACTOME_APOPTOSIS |
| REACTOME_G1_PHASE |
| REACTOME_DNA_REPLICATION |
| REACTOME_SIGNALING_BY_BMP |
| KEGG_FOCAL_ADHESION |
| KEGG_WNT_SIGNALING_PATHWAY |
| module 473 |
| BIOCARTA_TGFB_PATHWAY |
| REACTOME_ANTIGEN_PROCESSING_UBIQUITINATION_PROTEASOME_DEGRADATION |
| REACTOME_APOPTOSIS |
| REACTOME_G1_PHASE |
| REACTOME_DNA_REPLICATION |
| KEGG_FOCAL_ADHESION |
| KEGG_TGF_BETA_SIGNALING_PATHWAY |
| KEGG_WNT_SIGNALING_PATHWAY |
| module 474 |
| BIOCARTA_TGFB_PATHWAY |
| REACTOME_ANTIGEN_PROCESSING_UBIQUITINATION_PROTEASOME_DEGRADATION |
| REACTOME_EXTRINSIC_PATHWAY_FOR_APOPTOSIS |
| REACTOME_G1_PHASE |
| REACTOME_DNA_REPLICATION |
| REACTOME_SIGNALING_BY_BMP |
| KEGG_FOCAL_ADHESION |
| KEGG_WNT_SIGNALING_PATHWAY |
| module 475 |
| BIOCARTA_TGFB_PATHWAY |
| REACTOME_ANTIGEN_PROCESSING_UBIQUITINATION_PROTEASOME_DEGRADATION |
| REACTOME_EXTRINSIC_PATHWAY_FOR_APOPTOSIS |
| REACTOME_G1_PHASE |
| REACTOME_DNA_REPLICATION |
| KEGG_FOCAL_ADHESION |
| KEGG_TGF_BETA_SIGNALING_PATHWAY |
| KEGG_WNT_SIGNALING_PATHWAY |
| module 476 |
| BIOCARTA_TGFB_PATHWAY |
| REACTOME_ANTIGEN_PROCESSING_UBIQUITINATION_PROTEASOME_DEGRADATION |
| REACTOME_G1_PHASE |
| REACTOME_DNA_REPLICATION |
| REACTOME_SIGNALING_BY_BMP |
| KEGG_APOPTOSIS |
| KEGG_FOCAL_ADHESION |
| KEGG_WNT_SIGNALING_PATHWAY |
| module 477 |
| BIOCARTA_TGFB_PATHWAY |
| REACTOME_ANTIGEN_PROCESSING_UBIQUITINATION_PROTEASOME_DEGRADATION |
| REACTOME_G1_PHASE |
| REACTOME_DNA_REPLICATION |
| KEGG_APOPTOSIS |
| KEGG_FOCAL_ADHESION |
| KEGG_TGF_BETA_SIGNALING_PATHWAY |
| KEGG_WNT_SIGNALING_PATHWAY |
| module 478 |
| BIOCARTA_TGFB_PATHWAY |
| REACTOME_APOPTOSIS |
| REACTOME_CLASS_I_MHC_MEDIATED_ANTIGEN_PROCESSING_PRESENTATION |
| REACTOME_G1_PHASE |
| REACTOME_DNA_REPLICATION |
| REACTOME_SIGNALING_BY_BMP |
| KEGG_FOCAL_ADHESION |
| KEGG_WNT_SIGNALING_PATHWAY |
| module 479 |
| BIOCARTA_TGFB_PATHWAY |
| REACTOME_APOPTOSIS |
| REACTOME_CLASS_I_MHC_MEDIATED_ANTIGEN_PROCESSING_PRESENTATION |
| REACTOME_G1_PHASE |
| REACTOME_DNA_REPLICATION |
| KEGG_FOCAL_ADHESION |
| KEGG_TGF_BETA_SIGNALING_PATHWAY |
| KEGG_WNT_SIGNALING_PATHWAY |
| module 480 |
| BIOCARTA_TGFB_PATHWAY |
| REACTOME_CLASS_I_MHC_MEDIATED_ANTIGEN_PROCESSING_PRESENTATION |
| REACTOME_EXTRINSIC_PATHWAY_FOR_APOPTOSIS |
| REACTOME_G1_PHASE |
| REACTOME_DNA_REPLICATION |
| REACTOME_SIGNALING_BY_BMP |
| KEGG_FOCAL_ADHESION |
| KEGG_WNT_SIGNALING_PATHWAY |
| module 481 |
| BIOCARTA_TGFB_PATHWAY |
| REACTOME_CLASS_I_MHC_MEDIATED_ANTIGEN_PROCESSING_PRESENTATION |
| REACTOME_EXTRINSIC_PATHWAY_FOR_APOPTOSIS |
| REACTOME_G1_PHASE |
| REACTOME_DNA_REPLICATION |
| KEGG_FOCAL_ADHESION |
| KEGG_TGF_BETA_SIGNALING_PATHWAY |
| KEGG_WNT_SIGNALING_PATHWAY |
| module 482 |
| BIOCARTA_TGFB_PATHWAY |
| REACTOME_CLASS_I_MHC_MEDIATED_ANTIGEN_PROCESSING_PRESENTATION |
| REACTOME_G1_PHASE |
| REACTOME_DNA_REPLICATION |
| REACTOME_SIGNALING_BY_BMP |
| KEGG_APOPTOSIS |
| KEGG_FOCAL_ADHESION |
| KEGG_WNT_SIGNALING_PATHWAY |
| module 483 |
| BIOCARTA_TGFB_PATHWAY |
| REACTOME_CLASS_I_MHC_MEDIATED_ANTIGEN_PROCESSING_PRESENTATION |
| REACTOME_G1_PHASE |
| REACTOME_DNA_REPLICATION |
| KEGG_APOPTOSIS |
| KEGG_FOCAL_ADHESION |
| KEGG_TGF_BETA_SIGNALING_PATHWAY |
| KEGG_WNT_SIGNALING_PATHWAY |

**Supplementary Table S3: Results of random walk algorithm**. In total, 23 pathways were identified as CVD related pathways.

| Pathway name | pvalue |
| --- | --- |
| BIOCARTA_FAS_PATHWAY | 0 |
| REACTOME_INTERFERON_SIGNALING | 0 |
| REACTOME_SLC_MEDIATED_TRANSMEMBRANE_TRANSPORT | 6.00E-04 |
| REACTOME_PRE_NOTCH_TRANSCRIPTION_AND_TRANSLATION | 6.00E-04 |
| REACTOME_PRE_NOTCH_EXPRESSION_AND_PROCESSING | 6.00E-04 |
| REACTOME_REGULATORY_RNA_PATHWAYS | 6.00E-04 |
| REACTOME_GLYCOSAMINOGLYCAN_METABOLISM | 0.0028 |
| PID_TELOMERASE_PATHWAY | 0.0138 |
| REACTOME_LIPID_DIGESTION_MOBILIZATION_AND_TRANSPORT | 0.0142 |
| PID_CMYB_PATHWAY | 0.0142 |
| PID_E2F_PATHWAY | 0.0144 |
| REACTOME_TRANSPORT_OF_INORGANIC_CATIONS_ANIONS_AND_AMINO_ACIDS_OLIGOPEPTIDES | 0.0144 |
| PID_HDAC_CLASSI_PATHWAY | 0.0144 |
| REACTOME_TRANS_GOLGI_NETWORK_VESICLE_BUDDING | 0.0144 |
| PID_P75_NTR_PATHWAY | 0.0144 |
| REACTOME_MITOTIC_PROMETAPHASE | 0.0144 |
| PID_RHOA_PATHWAY | 0.0146 |
| REACTOME_PURINE_METABOLISM | 0.0146 |
| BIOCARTA_MAPK_PATHWAY | 0.0146 |
| REACTOME_METABOLISM_OF_NUCLEOTIDES | 0.0146 |
| REACTOME_METABOLISM_OF_CARBOHYDRATES | 0.0146 |
| REACTOME_INTERFERON_GAMMA_SIGNALING | 0.0146 |
| REACTOME_SIGNALING_BY_ERBB4 | 0.0146 |
