# Supplementary material for: Characterization and Validation of ceRNA-Mediated Pathway–Pathway Crosstalk Networks Across Eight Major Cardiovascular Diseases
Source: Front Cell Dev Biol. 2022 Apr 1;10:762129. doi: 10.3389/fcell.2022.762129 (PMC9010821; doi:10.3389/fcell.2022.762129)
Supplement: Supplementary file 4 [file Table1.DOC]

| **Disease** | **Disease name** | **GSE number** | **Platform** | **Samples** | **ceRNA pairs** | **Number of edges** | **Number of nodes** |
| --- | --- | --- | --- | --- | --- | --- | --- |
| **CAD** | Coronary artery disease | GSE40231 | GPL570 | 278 | 236986 | 45276 | 1171 |
|  |  | GSE12288 | GPL96 | 222 | 25491 | 10775 | 782 |
| **HCM** | Hypertrophic cardiomyopathy | GSE36961 | GPL15389 | 145 | 50302 | 11823 | 901 |
| **HF** | Heart failure | GSE1145 | GPL570 | 90 | 52279 | 7600 | 844 |
|  |  | GSE2656 | GPL2041 | 49 | 17940 | 2623 | 491 |
|  |  | GSE5406 | GPL96 | 210 | 57256 | 32657 | 1028 |
|  |  | GSE57338 | GPL11532 | 313 | 95542 | 22652 | 1065 |
| **DCM** | Dilated cardiomyopathy | GSE3586 | GPL3050 | 28 | 8761 | 623 | 267 |
|  |  | GSE17800 | GPL570 | 48 | 40547 | 5706 | 747 |
| **MI** | Myocardial infarction | GSE48060 | GPL570 | 52 | 101896 | 23762 | 1043 |
|  |  | GSE60993 | GPL6884 | 33 | 32806 | 6499 | 695 |
|  |  | GSE62646 | GPL6244 | 98 | 91534 | 23512 | 1029 |
|  |  | GSE66360 | GPL570 | 99 | 134936 | 39066 | 1090 |
|  |  | GSE28454 | GPL6883 | 76 | 27271 | 7893 | 739 |
|  |  | GSE29111 | GPL570 | 52 | 40189 | 7238 | 800 |
|  |  | GSE34198 | GPL6102 | 97 | 356446 | 56226 | 1208 |
|  |  | GSE59867 | GPL6244 | 436 | 136969 | 32379 | 1097 |
|  |  | GSE49925 | GPL10558 | 338 | 52116 | 17062 | 949 |
| **PAH** | Pulmonary hypertension | GSE33463 | GPL6947 | 140 | 236397 | 58384 | 1158 |
| **ICM** | Ischemic cardiomyopathy | GSE1869 | GPL96 | 37 | 57922 | 32725 | 979 |
| **CHD** | Congenital Heart Disease | GSE34457 | GPL6102 | 43 | 332679 | 56510 | 1204 |

Supplementary Table 1
